# Family Bereavement Support Interventions in Specialist Adult Palliative Care: A Rapid Mixed‐Methods Systematic Review

**DOI:** 10.1111/jan.70193

**Published:** 2025-09-05

**Authors:** Torsten Schwalbach, Marco Riguzzi, Myrta Kohler, Rahel Naef

**Affiliations:** ^1^ Institute for Implementation Science in Health Care Faculty of Medicine, University of Zurich Zurich Switzerland; ^2^ Center of Clinical Nursing Science University Hospital Zurich Zurich Switzerland

**Keywords:** bereavement, death and dying, end‐of‐life, evidence synthesis, family support intervention, grief support, mixed‐methods review, nursing, palliative care, systematic reviews and meta‐analyses

## Abstract

**Aim:**

To synthesise evidence on the impact of pre‐ and post‐loss family support interventions on bereavement outcomes and families' perceptions of their usefulness and benefits in specialist palliative care.

**Design:**

A rapid mixed‐methods systematic review drawing on JBI and Cochrane guidance. Study quality was appraised using the Mixed‐Methods Appraisal Tool. Qualitative and quantitative data were analysed using a meta‐aggregation and narrative analysis approach combined with narrative synthesis.

**Data Source:**

We searched Medline, CINAHL, PsycINFO, Embase and Cochrane Library and included articles published between 2004 and 2024 that evaluated pre‐ and post‐loss family support in specialist adult palliative care and assessed bereavement outcomes.

**Results:**

The search yielded 3682 records. We included thirty‐nine mostly moderate to high‐quality studies (57% quantitative). Results suggest that pre‐loss support, like family‐focused interventions and communication during dying, may mitigate post‐loss anxiety, depression and grief. Individual and group post‐loss support interventions may reduce anxiety, distress and grief while improving well‐being. Families desire individualised and comprehensive pre‐ and post‐loss support, with few not needing or accepting it. Stigma associated with bereavement, support and barriers can hinder access.

**Conclusion:**

Included studies demonstrated mixed effects of pre‐ and post‐loss family support interventions, suggesting they are beneficial when accessible and tailored to family needs. High‐quality intervention research assessing a broader range of family bereavement outcomes is needed.

**Impact:**

Palliative care nurses and other health professionals should tailor their care to family needs, start family support before patient death and ensure equitable access to bereavement services. Our results may guide palliative care professionals in designing effective, personalised and accessible services and policymakers in allocating resources for bereavement care. Findings highlight research needs, including investigating barriers to care and accessibility of services. High‐quality research is needed to understand who benefits the most from health‐promoting bereavement support and why.

**Reporting Method:**

We adhered to the PRISMA guideline.

**Patient and Public Contribution:**

No Patient and Public Contribution.

**Protocol Registration:**

Open Science Framework https://osf.io/36jeu

**Summary**

- Contribution of this paper:
  ○ This review comprehensively compares and structures qualitative and quantitative research evidence on pre‐ and post‐loss family support interventions targeting bereavement outcomes in specialist palliative care, including interventions during end‐of‐life and dying, individual follow‐up and group bereavement support, and families' experiences with care.
  ○ Most pre‐ and post‐loss interventions seem to prevent or improve adverse mental health outcomes in bereaved families, like prolonged grief, depression and anxiety, and alleviate grief symptoms, whereas the impact on quality of life and well‐being is less well‐researched and yields mixed results. Families experience such bereavement support as helpful, but there exists a stigma concerning bereavement and support services.
  ○ Nurses and other palliative care professionals should provide family‐focused support services that begin during the last phase of a person's life and continue to follow family members into the bereavement phase. These services should include ongoing needs assessments, comprehensive communication and tailored, accessible follow‐up care supported by organisations and policymakers.

## 1 Introduction

Caregiving for a close person with a life‐limiting illness, followed by loss, is a stressful experience for family members (Breen et al. 2018; Holtslander et al. 2017). It significantly impacts their quality of life and physical and mental health, both during their close person's end‐of‐life and in their subsequent bereavement (Breen et al. 2020; Nielsen et al. 2017; Wen et al. 2019). The prevalence of complicated grief in bereaved family members ranges from 5% to 33%, and up to 88% experience symptoms of anxiety and/or depression after losing a close person due to a serious illness (Garrouste‐Orgeas et al. 2023; Hamano et al. 2021; Kustanti et al. 2022; Wu et al. 2022). Almost half report low resilience (Wu et al. 2022) and low emotional functioning (Ham et al. 2022). Despite such a high burden, many cope with their loss through support within their own family, their social network and the care they receive from health professionals in the course of their close person's palliative treatment, while only a small proportion receive structured support (Lichtenthal et al. 2024).

An evidence‐informed, three‐tiered, risk‐ and needs‐oriented model has been proposed to meet varying types and degrees of bereavement support needs (Aoun et al. 2015; Killikelly et al. 2021; National Institute for Clinical Excellence 2004). Tier one, or *general* interventions, should be offered to all bereaved persons and include information about bereavement and relevant support. Tier two, or *selective* interventions, targets families at risk for challenging bereavement experiences and medium needs and includes non‐specialist support by trained volunteers or health professionals, self‐help groups, or follow‐up contact. Tier three, or *indicated* interventions, are specialist mental health interventions and are recommended for high‐risk groups with adverse mental health and high levels of need.

Family bereavement support interventions are defined as “all services provided to support family members during palliative care and after the death” (Keegan et al. 2021), starting before the death of the close person and stretching into bereavement (National Institute for Clinical Excellence 2004; Hudson et al. 2018). As such, the term *bereavement support* refers to a range of different types of interventions that are offered in end‐of‐life care, during and after the dying of a close person, intending to increase the quality of care and bereavement outcomes for family members (National Institute for Clinical Excellence 2004; Hudson et al. 2018). Recent reviews found that bereavement support is beneficial for family members (Finucane et al. 2025) bereaved through advanced illness (Harrop, Morgan, et al. 2020; Kustanti et al. 2021; Johannsen et al. 2019) and for those whose close person died in an acute hospital setting (Boven et al. 2022; Green et al. 2022). Bereavement support interventions improve mental health, quality of life and coping with bereavement (Finucane et al. 2025; Kustanti et al. 2021; Johannsen et al. 2019), help to process loss and grief, provide a sense of mastery and social support and enable moving on (Harrop, Morgan, et al. 2020). *Indicated* interventions (e. g. mental health interventions such as psychotherapy, grief counselling) (Johannsen et al. 2019; Waller et al. 2016) have shown effectiveness in alleviating grief symptoms. These reviews provide an important synthesis of the current evidence on bereavement support across settings and populations. However, most focus on either *selective* and/or *indicated* support (Harrop, Morgan, et al. 2020; Johannsen et al. 2019; Waller et al. 2016) or lack a clear definition of bereavement support or differentiation between tiers. Additionally, they are not specific to specialist palliative care.

Bereavement support for families has been identified as a crucial element of specialist palliative care to alleviate the distress caused by unmet needs during the end‐of‐life and bereavement periods while minimising the risk of adverse health outcomes (National Institute for Clinical Excellence 2004; Hudson et al. 2018; Akechi et al. 2022). Palliative care nurses have been called to play a pivotal role in providing support to family members before and during the death of their close person and in the subsequent period of bereavement (Hudson et al. 2018, 2021; Alam et al. 2020; Radbruch et al. 2020). However, bereavement support within palliative care is frequently inadequate (Boven et al. 2022; Takeuchi et al. 2022; Breen and Moullin 2022; Breen et al. 2014), and palliative care nurses and other professionals express uncertainty about how to best support families, particularly in bereavement (Takeuchi et al. 2022). Scarce resources challenge them to decide carefully about tailoring support to specific family needs (Boven et al. 2022). A comprehensive analysis of the evidence on family bereavement support in specialist palliative care is essential to inform accessible evidence‐based, targeted interventions (Keegan et al. 2021).

## 2 The Review

### 2.1 Aims

This systematic review focuses on family bereavement support interventions that reflect *general* and *selective* bereavement support (Aoun et al. 2015; Killikelly et al. 2021; National Institute for Clinical Excellence 2004), which are an integral part of specialist palliative care services that nurses are called to offer (Hudson et al. 2018; Akechi et al. 2022). It aims to synthesise qualitative and quantitative evidence on pre‐ and post‐loss family support interventions' impact on bereavement outcomes such as coping and well‐being (Harrop, Scott, et al. 2020) and families' perceptions of their usefulness and benefits. The research questions were: (1) What is the impact of bereavement support interventions on family health and coping with death and loss in specialist adult palliative care? (2) How do families appraise the usefulness/benefits of such interventions for their health, well‐being and coping in specialist adult palliative care?

### 2.2 Design

We undertook a rapid mixed‐methods systematic review to gain a comprehensive understanding of the current evidence on the impact of *general* and *selective* bereavement support interventions offered in specialist palliative care on bereavement outcomes. This review was conducted in preparation for an implementation research project (BEST for Family), which adapts, implements and evaluates an evidence‐based family bereavement support pathway in specialist palliative care (Kohler et al. 2025). To ensure a comprehensive synthesis of different types of research evidence, we employed a convergent segregated review design, drawing on JBI mixed‐methods systematic review guidance. We used a rapid approach to provide timely and high‐quality evidence for our project, drawing on the Cochrane Rapid Reviews guidance (Garritty et al. 2021). Rapid reviews are an efficient tool for generating evidence promptly, providing a basis for decision‐making in healthcare (Garritty et al. 2021; Lizarondo et al. 2020). Therefore, we combined JBI and Cochrane recommendations to generate robust evidence in a timely manner. Covidence (www.covidence.org 2023) was used to streamline the review process and the PRISMA guideline for reporting it (Page et al. 2021). The protocol was pre‐registered on OSF (Schwalbach et al., n.d.).

### 2.3 Search Methods

Embase (Elsevier), Medline (via EBSCO), CINAHL with full text (via EBSCO), PsycINFO (via EBSCO) and Cochrane Library (Cochrane Interface) were searched using key terms in different variations and synonyms, combined with Boolean operators, wildcards and thesaurus keywords: Family, bereavement or grief, bereavement support, palliative care (see Table 1 and File S1). The research team and a librarian developed a sensitive search strategy, which was initially tested and subsequently refined. Where appropriate, specified search terms and language restrictions were applied. The librarian searched the databases on March 31, 2023, and the first author updated the search on February 28, 2024.

**TABLE 1:** Example search string (Medline).

#	Query	Limiters/Expander
S7	S6 NOT (TI (‘pediatric’ OR ‘childhood’ OR ‘children’ OR ‘neonatal’ OR ‘perinatal’) NOT TI (‘adult children*’ OR ‘adult child*’))	Limiters—Language: English, French, German
S6	S5 NOT ((MH “infant+” OR MH “child+” OR MH adolescent) NOT MH “adult+”)	
S5	S4 NOT (MH “animals+” NOT MH “humans”)	
S4	S1 AND S2 AND S3	
S3	(MH “Hospice and Palliative Care Nursing”) OR (MH “Hospice Care”) OR (MH “Hospices”) OR (MH “Palliative Care”) OR (MH “Palliative Medicine”) OR (MH “Terminal Care”) OR TI (palliation OR ((palliative OR ‘end of life’ OR eol OR hospice* OR terminal) N3 (treatment* OR nursing* OR therap* OR consultation* OR service* OR care OR unit* OR medic* OR situation*))) OR AB (palliation OR ((palliative OR ‘end of life’ OR eol OR hospice* OR terminal) N3 (treatment* OR nursing* OR therap* OR consultation* OR service* OR care OR unit* OR medic* OR situation*)))	
S2	((MH “Bereavement+”) AND ((MH “Social Support+”) OR (MH “Psychosocial Intervention”) OR (MH “Counseling”) OR TI (support* OR intervention* OR service* OR program* OR counsel*) OR AB (support* OR intervention* OR service* OR program* OR counsel*))) OR TI (‘bereavement care*’ OR ‘bereavement practi*’ OR ‘bereavement follow‐up*’ OR ((bereavement OR death OR ‘end of life’ OR eol OR conclusion OR loss OR grief OR postdeath) N3 (support* OR intervention* OR service* OR program* OR counsel*))) OR AB (‘bereavement care*’ OR ‘bereavement practi*’ OR ‘bereavement follow‐up*’ OR ((bereavement OR death OR ‘end of life’ OR eol OR conclusion OR loss OR grief OR postdeath) N3 (support* OR intervention* OR service* OR program* OR counsel*)))	
S1	((MH “Grief+”) AND (TI (people OR person* OR famil* OR families OR familial OR ‘family member*’ OR ‘family members’ OR carer* OR caregiv* OR relative* OR relation OR relations OR spouse* OR partner* OR husband* OR widower* OR wife OR wives OR widow* OR volunteer* OR ‘close other*’ OR ‘next of kin*’) OR AB (people OR person* OR famil* OR families OR familial OR ‘family member*’ OR ‘family members’ OR carer* OR caregiv* OR relative* OR relation OR relations OR spouse* OR partner* OR husband* OR widower* OR wife OR wives OR widow* OR volunteer* OR ‘close other*’ OR ‘next of kin*’))) OR (MH “Family+”) OR (MH “Caregivers”) OR TI (bereaved OR ‘bereaved person*’ OR ((bereaving OR grief OR grieving OR mourning OR mournful OR coping) N3 (people OR person* OR family OR families OR familial OR carer* OR caregiv* OR relative* OR relation OR relations OR spouse* OR partner* OR husband* OR widower* OR wife OR wives OR widow* OR volunteer*))) OR AB (bereaved OR ‘bereaved person*’ OR ((bereaving OR grief OR grieving OR mourning OR mournful OR coping) N3 (people OR person* OR family OR families OR familial OR carer* OR caregiv* OR relative* OR relation OR relations OR spouse* OR partner* OR husband* OR widower* OR wife OR wives OR widow* OR volunteer*)))	

### 2.4 Inclusion and Exclusion Criteria

Eligible studies had (1) to include family members or close persons of dying or deceased patients (population), (2) take place in specialist adult palliative care services in acute, hospice, or community settings, (3) evaluate pre‐ and post‐loss family support interventions, services or programs provided or facilitated by palliative care specialists of any profession and (4) address core family bereavement outcomes (health, well‐being, coping) (Harrop, Scott, et al. 2020) or families' appraisal of usefulness or benefits of bereavement support. Family members were defined as close persons from the patient's perspective. Legal or blood relations were not required. We defined specialist palliative care as all services provided by palliative care specialists of any profession, regardless of the healthcare setting in which they are delivered. Pre‐loss family support interventions are those offered during the last three to 6 months before death, and post‐loss interventions were defined to be delivered after the death of the patient, with no time limit thereafter. Studies evaluating *indicated* support were excluded because they are usually outside the scope of palliative care staff, and their effectiveness has already been well investigated (Johannsen et al. 2019; Waller et al. 2016). Quantitative, qualitative and mixed‐methods studies published in English, French or German in peer‐reviewed journals between 2004 and 2024 were included. We chose this time limit because the seminal publication on the three‐tiered model of bereavement care was released in 2004 (National Institute for Clinical Excellence 2004).

### 2.5 Search Outcome

Citations from the librarian's search were uploaded to EndNote (The EndNote Team 2013), and duplicates were removed. The first author used keywords reflecting the exclusion criteria in different variations to identify irrelevant studies using EndNote's built‐in search tool, namely paediatric, child, intensive care unit, critical care nurse, adolescent, emergency department, emergency room, violence, accident, disaster, psychotherapy, grief therapy, interpersonal therapy, cognitive behaviour therapy, review, meta‐analysis, synthesis and conference (File S2). The first author then uploaded all remaining citations into Covidence. Titles and abstracts were screened by the first author for eligibility. Studies that met the inclusion criteria were retrieved in full text, and their details were imported into Covidence. Next, the full texts were assessed in detail against the inclusion criteria independently by the first and last author. Interrater agreement before discussion was moderate (Cohen's *Ƙ* = 0.439, 95% CI from 0.307 to 0.570). Any disagreements that arose between the reviewers were resolved through discussion and consultation with a third reviewer.

### 2.6 Quality Appraisal

The Mixed‐Methods Appraisal Tool (MMAT) Version 2018 was used for quality assessment (Hong et al. 2018). The MMAT is a validated tool applicable to qualitative, quantitative and mixed‐methods studies with five items per study design (answered with *yes*/*can't tell*/*no*), resulting in a score ranging from 0% to 100%. For every *no* or *can't tell*, 20 percentage points are subtracted. The overall mixed‐methods quality score cannot exceed the score of its weakest component and is therefore determined by the lowest score. For multi‐method studies, the overall score is the mean of the sums of qualitative and quantitative ratings. A 100%–80% rating is considered high, 80%–40% moderate and 40%–0% low quality. The first author appraised all included studies. Two other researchers independently assessed a random sample of 20% for quality control. All three researchers resolved disagreements through discussion. We did not exclude studies based on their quality score.

### 2.7 Data Abstraction

The JBI qualitative data (Lockwood et al. 2020) and a modified Cochrane Collaboration quantitative data extraction form (The Cochrane Collaboration 2023) were imported into Covidence and used for data extraction (File S3). Study aims, design, sample characteristics, intervention/phenomenon of interest, study endpoints, measurements, analysis methods and results, including effect measures, if provided, were extracted. Qualitative findings were extracted as verbatim statements with a corresponding illustration (participant voice) and rated for credibility (Lockwood et al. 2020). A verbatim statement was defined as unequivocal if accompanied by an illustration that is beyond reasonable doubt and not open to challenge; as credible if it was accompanied by an illustration lacking clear association with it, thereby open to challenge; or as unsupported if the findings were not substantiated with data (Lockwood et al. 2020). We included only data rated as unequivocal or credible. The extracted data were then exported from Covidence into a Microsoft 365 Excel spreadsheet. Two other researchers independently extracted a random sample of 20% for quality control purposes. The authors compared, discussed and refined the extracted data if necessary.

### 2.8 Data Synthesis

Following the convergent segregated methodology (Lizarondo et al. 2020), we analysed the qualitative and quantitative data separately before integrating and synthesising them narratively. To become familiar with the data, we first employed an inductive mapping process to display qualitative and quantitative findings. Then, findings statements were sorted separately for quantitative and qualitative data along the dying trajectory (pre‐loss, dying, post‐loss) and intervention format (individual or group delivery). Data not referring to a specific intervention but to bereavement support in general were clustered separately. The first and last authors then reviewed and refined these clusters through a systematic sorting process and regular data analysis meetings. Per quantitative clusters, data were compared and contrasted and analysed according to intervention type, characteristics and outcomes. We critically reviewed them for consistency and divergences in effectiveness and synthesised the findings narratively. Qualitative findings were transferred into a Microsoft 365 Excel spreadsheet using the clusters as an organising structure. Employing a meta‐aggregation approach (Lockwood et al. 2015, 2020), we combined extracted findings into different categories, which required at least two qualitative findings per category. Finally, the results of the quantitative and qualitative analyses were integrated by comparing and contrasting them in a table, examining their commonalities and differences to achieve an overall analysis and interpretation of the data. Additionally, the methodological quality of the studies was taken into account throughout the analysis and interpretation process. The data integration results were then synthesised in narrative form and presented in six inductively developed categories.

## 3 Results

### 3.1 Study Characteristics

**FIGURE 1:**
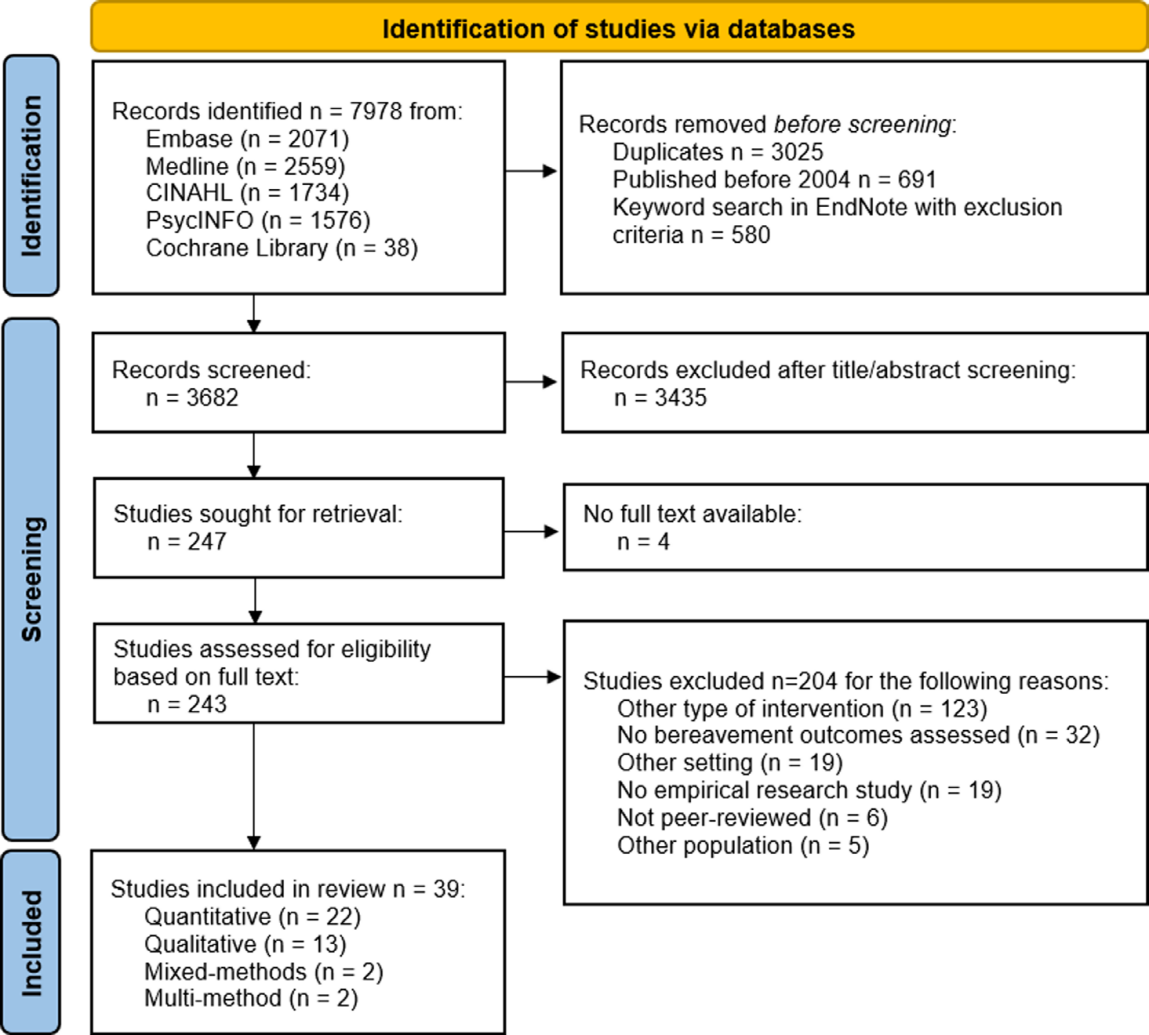
PRISMA flow chart.

We screened 3682 records for eligibility, of which 243 were assessed as full texts. A total of 39 studies (Wu et al. 2022; Agnew et al. 2008; Aoun, Breen, et al. 2018; Aoun, Ewing, et al. 2018; Aoun et al. 2017; Cronfalk et al. 2009, 2010; Davis et al. 2020; Dionne‐Odom et al. 2016; Goebel et al. 2017; Grande et al. 2017; Hudson et al. 2005, 2015; Hudson 2006; Kirby et al. 2018; Levesque et al. 2023; Lundberg et al. 2013; Magill 2009a, 2009b; Makarem et al. 2018; Maze et al. 2022; McGinley and Waldrop 2020; McGrath et al. 2010; Mooney et al. 2024; Muta et al. 2014; Nappa and Bjorkman‐Randstrom 2020; Nappa et al. 2016; Olsson et al. 2017; Petursdottir et al. 2020; Ramstad et al. 2023; Reblin et al. 2019; Roberts and McGilloway 2008; Supiano et al. 2020; Tabler et al. 2015; Veerbeek et al. 2008; von Heymann‐Horan et al. 2018; Walsh et al. 2007; Wittenberg‐Lyles et al. 2015; Yamaguchi et al. 2017) were included (Figure 1). Records that did not meet the inclusion criteria were excluded for the following main reasons: other type of intervention (*n* = 123), no bereavement outcomes assessed (*n* = 32), other setting (*n* = 19) (see Figure 1 and File S4 for details of all excluded records). Most included studies had been conducted in Australia (*n* = 9), the United States of America (*n* = 8), Sweden (*n* = 6) and Canada (*n* = 5), either in hospice (*n* = 11), acute in‐ or outpatient (*n* = 10), community (*n* = 4), home (*n* = 7) or across settings (*n* = 7).

### 3.2 Study Design and Quality

Over half of the studies used quantitative (*n* = 22, 57%) (Aoun, Breen, et al. 2018; Aoun, Ewing, et al. 2018; Aoun et al. 2017; Davis et al. 2020; Dionne‐Odom et al. 2016; Goebel et al. 2017; Grande et al. 2017; Hudson et al. 2005, 2015; Levesque et al. 2023; Mooney et al. 2024; Nappa et al. 2016; Olsson et al. 2017; Petursdottir et al. 2020; Ramstad et al. 2023; Reblin et al. 2019; Roberts and McGilloway 2008; Supiano et al. 2020; Veerbeek et al. 2008; von Heymann‐Horan et al. 2018; Walsh et al. 2007; Yamaguchi et al. 2017), one‐third qualitative (*n* = 13, 33%) (Agnew et al. 2008; Cronfalk et al. 2009; Cronfalk et al. 2010; Kirby et al. 2018; Lundberg et al. 2013; Magill 2009a, 2009b; Makarem et al. 2018; Maze et al. 2022; McGrath et al. 2010; Muta et al. 2014; Nappa and Bjorkman‐Randstrom 2020; Tabler et al. 2015) and 10% mixed or multi‐method designs (*n* = 4) (Wu et al. 2022; Hudson 2006; McGinley and Waldrop 2020; Wittenberg‐Lyles et al. 2015) (Table 2). Quantitative studies employed mostly observational designs (*n* = 11, 50%), followed by experimental controlled (*n* = 8, 36%) and quasi‐experimental designs (*n* = 3, 14%). Qualitative studies used mostly descriptive exploratory approaches. Thematic (*n* = 4, 31%) and content analyses (*n* = 4, 31%) were the most frequent analysis methods. Two studies (Olsson et al. 2017; Walsh et al. 2007) did not report their analysis method. Of the multi‐ or mixed‐methods studies (Wu et al. 2022; Hudson 2006; Wittenberg‐Lyles et al. 2015), three of four did not use a data integration approach.

**TABLE 2:** Overview of included studies.

Author, Year, Country	Aim	Design	Setting	Sample^a^	Intervention (I), Comparator (C),^b^ Phenomenon of Interest (PoI)	Data collection	Outcomes/phenomenon	Key findings	Quality score^c^ (%)
Agnew et al. (2008) Ireland	Retrospectively explore certain pre‐ and post‐bereavement experiences of partners bereaved through cancer	Qualitative descriptive, retrospective, cross‐sectional, thematic content analysis	Community	Partners or spouses of deceased service users (*n* = 10)	PoI: Bereavement experiences	Semi‐structured interviews, bereavement	Pre‐ and post‐bereavement experiences	Four themes: cancer journey; impact of bereavement; process of adjustment and change; experience of support services	100
Aoun et al. (2017) Australia	Identify patterns of bereavement support in palliative care services based on the experience of bereaved	Quantitative, observational, inferential statistical	Across settings	Bereaved family members (*n* = 506), age mean 77 (SD 13.9)	I: Bereavement support	Questionnaire, 6–24 months after bereavement	Experiences with bereavement support	Mixed experiences with follow‐up from 3 weeks up to 6 months in qualitatively analysed open‐ended survey questions; some found it helpful, others did not.	80
Aoun, Breen, et al. (2018) Australia	Assess nature and extent of the sources of bereavement support and their perceived helpful or unhelpfulness; identify nature and extent of the functional types of bereavement support	Quantitative, observational, descriptive statistical	Across settings	Bereaved family members (*n* = 678), age mean 62.4 (SD 12.2)	I: Bereavement support	Questionnaire, 6–24 months after bereavement	Perceived helpfulness (SDQ)	Professional sources were the least used and had the highest proportions of perceived unhelpfulness	80
Aoun, Ewing, et al. (2018) Australia	Investigate the extent to which using the CSNAT‐Intervention during the caregiving period has affected bereaved family caregivers' perceptions of adequacy of support, their grief and well‐being and achievement of their preferred place of death	Quantitative, observational, inferential statistical	Community palliative care	Bereaved family caregivers (*n* = 212), age mean 64.6 (SD 11.8)	I: CSNAT C: Ø	Questionnaire, 4–6 months after bereavement	Perceived adequacy of support provided for the caregiver during end‐of‐life care (CSNAT, primary outcome), level of grief (TRIG), mental and physical well‐being (SF12v2)	Intervention group perceived that their pre‐bereavement support needs had been adequately met to a significantly greater extent than the control group (Cohen's *d* = 0.43, *p* < 0.001) No impact on grief or well‐being proven.	100
Cronfalk et al. (2010) Sweden	Explore relatives' experiences of receiving soft tissue massage while caring for a dying family member in the home	Qualitative descriptive, retrospective, cross‐sectional, content analysis	Palliative home care	Relatives of dying family members (*n* = 19)	I: Soft tissue massage C: Ø	Single interviews, one to 2 weeks after intervention	Experiences of receiving soft tissue massage	Soft tissue massage gave the relatives feelings of ‘being cared for’, ‘body vitality’ and ‘peace of mind’	80
Cronfalk et al. (2010) Sweden	Explore how bereaved relatives experienced early intervention with soft tissue massage during the first 4 months after the death of a family member	Qualitative descriptive, retrospective, cross‐sectional, content analysis	Acute palliative care	Relatives of family members, who died in a large palliative care unit (*n* = 18)	I: Soft tissue massage C: Ø	Single interviews, 1 week after intervention	Experiences of early intervention with soft tissue massage during the first 4 months after the death of a family member	Soft tissue massage proved to be helpful and generated feelings of consolation in the first 4 months of grieving	80
Davis et al. (2020) Australia	Assess feasibility of the intervention for carers of patients in palliative care, evaluate intervention effectiveness	Quantitative, experimental, controlled parallel randomised, inferential statistical	Acute palliative care	Carers of patients with life‐limiting illness (*n* = 55), age mean 58 (SD 12)	I: Treatment as usual plus skill‐based booklet and telephone support C: Usual care	Questionnaire, baseline, 1‐month follow‐up, 6‐month follow‐up	Experiential avoidance (AAQ‐II), valued living (VLQ), grief (PG‐13), psychological distress (HADS), anticipatory grief (PG‐12)	Preliminary effectiveness analyses showed a small, non‐significant^d^ effect on grief (1‐month follow‐up: *d* = 0.14, *p* > 0.05, 6‐month post‐loss follow‐up: *d* = 0.26, *p* > 0.05) and psychological distress (1‐month follow‐up: *d* < 0.01, *p* > 0.05, 6‐month post‐loss follow‐up: *d* = 0.52, *p* > 0.05)	40
Dionne‐Odom et al. (2016) USA	Determine the effect of early versus delayed initiation of a palliative care intervention and assess after‐death depressive symptoms and grief scores in both groups	Quantitative, experimental, controlled parallel randomised, inferential statistical	Medical center	Caregivers of cancer patients (*n* = 122), age mean 61 (SD 10)	I: Early group palliative care (within 60 days of diagnosis) C: Delayed group palliative care	Questionnaire, 8 and 12 weeks after death	Depressive symptoms (CESD‐D Scale), grief (PG13), quality of life (CG QOL‐C), caregiver burden (MBCB)	CGs' depressive symptoms and complicated grief scores 8 and 12 weeks after care recipients' deaths were not significantly^d^ different based on the timing of EPC support (*p* = 0.88 and *p* = 0.51, respectively)	20
Goebel et al. (2017) Germany	Inquiries about recipient's reactions and opinions on receiving a bereavement anniversary card	Quantitative, observational, descriptive statistical	Acute palliative care	First contact person of deceased patients (*n* = 24), age (45 to over 75)	I: Bereavement anniversary card C: Ø	Questionnaire, at least 1 year after patient death	Reactions on receiving a bereavement anniversary card	Almost all recipients felt pleased, grateful and consoled receiving the card, appropriate to send a card exactly 1 year after patient's death, important to design card personally, card can cause sadness	40
Grande et al. (2017) UK	To test the impact on family carers of a CSNAT‐Intervention	Quantitative, experimental, controlled stepped wedge, cluster part‐randomised, inferential statistical	Palliative home care	Next‐of‐kin of patients who died during trial period (*n* = 681)	I: CSNAT C: Usual care	Questionnaire, 4–5 months post bereavement	Distress (Distress thermometer), early grief (TRIG), physical health (SF12v2, general practitioner contacts post bereavement), psychological health (SF12v2), needs met (CSNAT)	Significantly^d^ lower levels of early grief (TRIG‐1: −1.96, *p* = 0.038), better psychological (SF‐12 mental: 2.58, *p* = 0.049) and physical health (SF‐12 physical: 3.09, *p* = 0.011)	60
Hudson et al. (2005) Australia	To examine the effectiveness of a psychoeducational intervention to enhance the support and guidance	Quantitative, experimental, controlled parallel randomised, inferential statistical	Home‐based palliative care	Primary family caregivers (*n* = 106), age mean 60 (SD 14)	I: Usual care and psychoeducational intervention C: Usual care	Questionnaire, 6 weeks post‐loss	Anxiety (HADS), reward (Rewards of caregiving scale, 4 items excluded)	No significant^d^ intervention effects on anxiety and reward (*p* > 0.05)	20
Hudson et al. (2015) Australia	To evaluate a one‐on‐one psychoeducational intervention for caregivers of patients with advanced cancer receiving home‐based palliative care	Quantitative, experimental controlled parallel randomised, inferential statistical	Home‐based palliative care	Primary family caregivers of advanced cancer patients (*n* = 298), age mean 59 (SD 14)	I: Psychoeducational intervention plus standard care (Intervention 1: 1 visit, intervention 2: 2 visits) C: Usual care	Questionnaire, bereavement 8 weeks post‐loss	Anxiety and distress (GHQ)	Significant^d^ decrease in distress at 8 weeks post death compared to baseline (*p* = 0.044) with Cohen's *d* = 0.58 in the one‐visit intervention group compared to control group; no significant^d^ difference between two‐visit intervention and control group	40
Hudson (2006) Australia	Explore caregiver perceptions of their relative's death and how well they were coping since the death	Multi‐method: quantitative observational, descriptive statistical; qualitative descriptive, content analysis	Across settings	Caregivers of patients involved in an RCT, where patients have died 6 weeks before (*n* = 45), age mean 59, SD 14 (22–88)	PoI: Caregivers' coping and their perceptions of their relative's death	Structured interviews, 2 months after death	Caregiver perceptions of their relative's death and how well they were coping since the death	Caregivers noted the significant benefits of receiving comprehensive information to prepare them for the future and expressed appreciation for the support	0
Kirby et al. (2018) Australia	Examine the experiences of bereaved family caregivers and their impressions of and interactions with bereavement support	Qualitative descriptive, retrospective, cross‐sectional, thematic analysis	Specialist palliative care	Bereaved family caregivers of patients cared for at a specialist palliative care (*n* = 15), age mean 84	I: Bereavement support	Semi‐structured interviews, 3–9 months after a first pre‐bereavement interview	Experiences of bereaved family caregivers and their impressions of and interactions with bereavement support	Four prevalent themes: (1) sociocultural constructions of bereavement support as for the incapable or socially isolated; (2) perceptions of bereavement support services as narrow in scope; (3) the “personal” character of bereavement and subsequent incompatibility with formalised support; (4) issues around timing and style of approaches to being offered support	100
Levesque et al. (2023) USA	Evaluation of Grief Coach Program	Quantitative, observational, descriptive statistical	Hospice	Caregivers of deceased palliative care patients (*n* = 350), age mean 54 (SD 15)	I: Grief Coach (text‐based program) C: Ø	Questionnaire 13 months after bereavement	SDQ	Grief Coach rated very helpful and supportive for grief (almost 75% of subscribers); particularly helpful for longer enrolled subscribers (enrollment > 6 months: Cohen's *h* = 0.20, fisher's exact probability test *p* = 0.711)	100
Lundberg et al. (2013) Sweden	Explore the supportive interactions that family members experience in palliative care and the emotions that they associate with these interactions	Qualitative descriptive, retrospective, cross‐sectional, content analysis	Palliative care service	Family members over 18 years of hospice patients participating in the hospice support program (*n* = 25)	PoI: Family members' experiences of supportive interactions in palliative care and associated emotions	Semi‐structured interviews, about half a year after death	Experiences of supportive interactions in the palliative care unit and on the family members' reactions	Five categories of supportive interactions linked with emotional consequences: (1) informational support, (2) supportive encounters, (3) professional focus of staff, (4) supportive environment, (5) bereavement support	100
Magill (2009a) Canada	Learn how music therapy sessions, held before the death of a loved one, impact spirituality in surviving caregivers of advanced cancer patients	Qualitative descriptive, retrospective, cross‐sectional, coding and grouping	Home‐based hospice	Caregivers, who were present in music therapy sessions with their loved ones before death (*n* = 7)	I: Music therapy C: Ø	Single interviews, bereavement	Spiritual meaning of music therapy experienced before the death of a loved one	Pre‐loss music therapy can potentially assist caregivers during times of bereavement, as they retain memories of joy and empowerment, rather than memories of pain and distress, and find meaning through transcendence	60
Magill (2009b) Canada	Examine the role of pre‐loss music therapy in palliative care for bereaved caregivers	Qualitative descriptive, retrospective, cross‐sectional, coding and grouping	Home‐based hospice	Adult bereaved caregivers of deceased patients who received palliative care (*n* = 7)	I: Music therapy C: Ø	Single interviews, bereavement	Significance of the role of music	4 themes: (1) music as a conduit, (2) music gets inside us, (3) live music makes a difference, (4) music is love	40
Makarem et al. (2018) Canada	Describe the experiences and opinions of caregivers regarding bereavement contact from healthcare providers	Qualitative grounded theory, retrospective, thematic analysis	Outpatient palliative care clinic	Bereaved caregivers of advanced cancer patients (*n* = 60), age median 60 (29–85)	I: Bereavement contact C: Ø	Semi‐structured interviews, one to 5 years after death	Experiences and opinions of caregivers regarding bereavement contact from healthcare providers	6 themes: (1) contact reflects caring, (2) contact offers support, (3) contact facilitates closure, (4) contact is a courtesy, (5) contact is not always necessary, (6) caregiver‐initiated contact	100
Maze et al. (2022) Canada	Better understand how bereaved family members perceive the care nurses provide in an inpatient hospice palliative care unit	Qualitative descriptive, retrospective, cross‐sectional, iterative coding approach	Hospice palliative care unit	Family members or close friends who had a significant other die in a palliative care unit (*n* = 10), age (60–89)	PoI: Family members' perception of nursing care in hospice palliative care unit	Semi‐structured interviews, 6 weeks to 6 months after bereavement	Perception of nursing care in the unit, and how it influences family members' bereavement experiences	3 themes: (1) creating a therapeutic environment, (2) creating a sense of ease, (3) creating a sense of meaning	100
McGinley and Waldrop (2020) USA	Explore how communication and care in the later stages of an advanced illness influence family caregivers' well‐being in bereavement	Mixed‐methods, concurrent triangulation: quantitative observational, descriptive statistical; qualitative descriptive, open, systematic, axial coding	Across settings	Bereaved caregivers over 18 (*n* = 108)	PoI: Influence of communication and care in advanced illness on caregiver's well‐being in bereavement	Interview, 4 months after death	Communication and care in the later stages of an advanced illness and influence on well‐being in bereavement	Families are more likely to feel emotionally prepared for loss and grief when healthcare providers are available to communicate in a concise, consistent and compassionate manner	0
McGrath et al. (2010) Australia	Explore bereavement survivors' perspectives on what support factors are most helpful to them	Qualitative descriptive, retrospective, cross‐sectional, thematic analysis	Hospice	Caregiver spouses (*n* = 77), age (38–91)	I: Bereavement support	Telephone survey, bereavement	Most helpful support factors for bereavement survivors	Helpful factors: hospice bereavement assistance before, during and following bereavement; having a support network, including informal support networks and formal support from general practitioners; and keeping active and busy	60
Mooney et al. (2024) United States	To determine the efficacy of Symptom Care at Home in decreasing daily Caregiver Burden in home hospice	Quantitative, experimental, controlled parallel randomised, inferential statistical	Hospice home care	Family caregivers of cancer patients enrolled in home hospice (*n* = 365)	I: Symptom Care at Home intervention (automated, digital support intervention with daily symptom assessment and automated, tailored coaching messages) plus usual care C: Usual Care	6 months after patient death	Bereavement adjustment (PSCaDLCS, IoDWL, TRIG, GDS, HSS, FMS)	Better six‐month bereavement adjustment in intervention group compared to control group partial *η* ^2^ = 0.245, *p* = 0.007)	60
Muta et al. (2014) Japan	Evaluate bereavement services provided by hospice/palliative care units and clarify demands for bereavement care	Qualitative descriptive, retrospective, cross‐sectional, content analysis	Palliative care unit	Primary caregivers of patients with cancer (*n* = 44), age (21–79)	I: Bereavement support	Semi‐structured interviews, bereavement	Experiences with bereavement services	Memorial services positively evaluated; bereaved family members have diverse needs and preferences for bereavement care, and personalised and supportive services are essential for meeting these needs	100
Nappa et al. (2016) Sweden	To investigate measurable effects on grief, anxiety and depression in those participating in bereavement groups compared to non‐participants	Quantitative, quasi‐experimental, inferential statistical	Palliative home care	Significant others of deceased persons, (*n* = 124), age median 64 (39–86)	I: Bereavement group with three to eleven people C: Non‐participants	Questionnaire, bereavement	Grief (TRIG), anxiety (HADS), depression (HADS)	No significant^d^ effect on grief, anxiety, depression	60
Nappa and Bjorkman‐Randstrom (2020) Sweden	To describe significant others' experiences of participation in bereavement groups	Qualitative descriptive, longitudinal, content analysis	Palliative home care	Significant others of deceased persons, (*n* = 46), age median 65 (43–75)	I: Bereavement group with three to eleven people C: Ø	Questionnaire, bereavement	Significant others' experiences of participation in bereavement groups	Eased grief close to death, but not for all; bereavement groups could enhance the self and offer relief from grief	80
Olsson et al. (2017) Sweden	To explore the psychosocial well‐being of young people who participated in support groups	Quantitative, observational, inferential statistical	Acute palliative care	Young, bereaved people (*n* = 29), age median 23 (16–28)	I: Support group following the loss of a parent to cancer C: Ø	Questionnaire, bereavement	Psychological and overall well‐being (SDQ)	Positive impact on psychosocial well‐being (mean difference: +1 on a 1–7 scale, *p* < 0.05)	60
Petursdottir et al. (2020) Iceland	To evaluate the impact of a nursing intervention offered to bereaved family cancer caregivers	Quantitative, quasi‐experimental, inferential statistical	Hospital‐based specialist palliative home care unit	Cancer bereaved family caregivers (*n* = 51), age between 31 and 61 and older	I: Family Strengths‐Oriented Therapeutic Conversation Intervention C: Ø	Questionnaire, bereavement 3, 5, 6 months post‐loss	Anxiety (DASS), depression, (DASS), distress (DASS)	Significantly^d^ decreased anxiety symptoms (group *p* = 0.048; time *p* = 0.0045), no improvement in depressive symptoms	80
Ramstad et al. (2023) Denmark	To investigate association between bereaved caregivers' self‐reported missing contact with healthcare professionals and symptoms of PGD and depressive symptoms 3 years after the loss.	Quantitative, observational, inferential statistical	Across settings	Caregivers of terminally ill patients (*n* = 3635), age mean 61	I: Bereavement contact C: Ø	6 months after bereavement	Missing contact with healthcare professionals (self‐developed item), depressive symptoms (BDI‐II), symptoms of PGD (PG‐13)	Experience of missing contact with palliative team 6 months after bereavement significantly associated with symptoms indicative of PGD (adjusted OR: 3.2, 95% CI from 1.5 to 6.9) and depression (adjusted OR: 2.0, 95% CI from 1.3 to 3.2) after 3 years	80
Reblin et al. (2019) USA	To identify the effects of hospice nurse supportive communication as well as caregiver‐nurse exchange of positive emotions on family caregiver depression during bereavement	Quantitative, observational, inferential statistical	Hospice	Family caregivers of home hospice cancer patients (*n* = 101), age mean 66 (SD 10)	I: Hospice nurse supportive communication and caregiver‐nurse exchange of positive emotions C: Ø	Questionnaire, bereavement 2,6,12 months post‐loss	Depression (GDS‐SF)	Hospice nurse supportive communication and positive emotion exchange were associated with caregivers' lower levels of depressive symptoms (β = −0.11, *p* = 0.048) in bereavement	60
Roberts and McGilloway (2008) Ireland	To evaluate a hospice‐based bereavement support service	Quantitative, observational, inferential statistical	Hospice	Bereaved clients who attended hospice services (*n* = 243)	I: Bereavement support	Questionnaire, bereavement	Bereavement follow‐up contact helpfulness (BSQ)	In qualitatively analysed open‐ended survey questions: bereavement follow‐up contact: considered helpful; monthly memorial ceremony: over one quarter would have liked the option of attending non‐religious service; bereavement information evening: most attendees satisfied	60
Supiano et al. (2020) USA	To develop, implement and evaluate a distance‐technology delivered grief support group program	Quantitative, quasi‐experimental, inferential statistical	Hospice	Bereaved family members (*n* = 28), age mean 57 (SD 13)	I: Telehealth grief support group C: Ø	Questionnaire, bereavement	Grief (BGQ), complicated grief (ICG‐r), grief severity and improvement (CGI‐I)	Significant^d^ decrease in grief score (pretest [+]/mean rank vs. post‐test (−)/mean rank: 4 [6.75] vs. 16 [11.44], *p* < 0.0034) and complicated grief score (pretest [+]/mean rank vs. post‐test (−)/mean rank: 4 [10.50] vs. 19 [12.32], *p* < 0.0026); grief severity declined (β = −0.290, SE = 0.031, *p* < 0.001) per week and grief improvement increased (β = 0.351, SE = 0.081, *p* < 0.000)	80
Tabler et al. (2015) United States	Explore the ways that bereavement needs of caregivers, either pre‐death or post‐death of their spouse/partner, were addressed	Qualitative phenomenological, retrospective, cross‐sectional, thematic analysis	Hospice	Bereaved family caregivers (*n* = 19), age 45 and older	PoI: Addressing of pre‐ and post‐death bereavement needs	Retrospective phone interviews, 8–32 months after death	Experiences with transition from hospice to bereavement	4 themes: (1) family versus patient focus, (2) caregiver preparation for loss, (3) addressing caregiver bereavement, (4) continuity of care	40
Veerbeek et al. (2008) Netherlands	To investigate the effects of using the Liverpool Care Pathway on communication on the level of bereavement in relatives after the patient's death	Quantitative, observational, inferential statistical	Various healthcare facilities	Relatives of deceased persons (*n* = 220), age mean 58 (SD 14)	I: Liverpool Care Pathway C: Ø	Questionnaire, 3 months post‐loss	Detachment (LDS), communication (VOICES)	Significantly^d^ lower detachment levels in intervention period (mean ± SD: 11 ± 5 vs. 9 ± 5, *p* = 0.01), when compared with baseline period; no enhanced communication quality	40
von Heymann‐Horan et al. (2018) Denmark	To evaluate effects of specialist palliative care and dyadic psychological intervention on caregiver anxiety and depression	Quantitative, experimental, controlled parallel randomised, inferential statistical	Hospital oncology departement	Caregiver of cancer patients (*n* = 258), age mean intervention 61 (SD 12), control 62 (SD 13)	I: Domus Intervention (Accelerated transition from hospital‐based oncological treatment to specialist palliative care at home) C: Usual care	Questionnaire, end‐of‐life and post‐loss week 2, months 2, 7, 13, 19	Anxiety (Symptom Checklist‐92‐ Anxiety subscale), depression (Symptom Checklist‐92 – Depression subscale)	Decreased anxiety symptoms overall (estimated difference, −0.12 compared to baseline mean ± SD: 1.00 ± 0.66; 95% CI: −0.22 to −0.01, *p* = 0.0266, Cohen's *d* = −0.19) and significantly^d^ decreased depression symptoms at 2 weeks (−0.28; 95% CI: −0.52 to −0.03; *p* = 0.0295, Cohen's *d* = −0.42) and 2 months post‐loss (−0.24; 95% CI: −0.48 to −0.01; *p* = 0.0448, Cohen's *d* = −0.37)	60
Walsh et al. (2007) UK	To evaluate the effectiveness of increased support for distressed, informal carers of patients receiving palliative care	Quantitative, experimental, controlled parallel randomised, inferential statistical	Specialist community palliative care	Informal caregivers (*n* = 271), intervention (*n* = 137), control (*n* = 134), age mean 56 (SD 14)	I: The carer advisor intervention (comprehensive assessment of domains of needs; discussing past, present and future issues; provision of advice, information, and emotional support) C: Usual care	Questionnaire, end‐of‐life and 4 months after death	Bereavement (Core Bereavement Items), carer strain (Carer Strain Index), psychological distress (GHQ‐28), quality of life (CG‐QOL‐C)	No significant^d^ effects on psychological distress, quality of life, or bereavement; qualitative benefit: additional emotional support, added information, advice, or practical or financial help	60
Wittenberg‐Lyles et al. (2015) USA	To assess the potential of a secret Facebook group for bereaved hospice caregivers	Mixed‐methods: quantitative observational, descriptive statistical; qualitative descriptive, content analysis	Hospice	Bereaved caregivers (*n* = 16), age mean 49 (SD 16)	I: Secret Facebook support group C: Ø	Questionnaire, bereavement	Anxiety (GAD‐7), depression (PHQ‐9)	Group participants reported lower anxiety and depression mean scores at study completion compared to first measure	20
Wu et al. (2022) Canada	Determine the prevalence of prolonged grief disorder (PGD) and self‐reported resilience among bereaved caregivers within a palliative care program	Multi‐method: quantitative observational, descriptive statistical; qualitative descriptive, thematic analysis	Community palliative care	Caregivers of palliative care patients (*n* = 427), aged between 35 and 75 or older	PoI: Prevalence of PGD and resilience among bereaved caregivers	Questionnaire, 3 months after death	Experiences with bereavement support services and activities the participants found helpful in coping with the loss	Healthcare providers and support groups helped cope with the loss; counselling with someone who “gets grief” was important; being with others facing similar situations helped them to feel comfortable in expressing their grief	0
Yamaguchi et al. (2017) Japan	To explore associations between end‐of‐life discussions and bereaved families' depression and complicated grief and the quality of patient death and end‐of‐life care	Quantitative, observational, inferential statistical	Specialist palliative care	Bereaved family members of deceased cancer patients (*n* = 9123), age under/equal 50 to over/equal 71	I: End‐of‐life discussions C: Ø	Questionnaire, bereavement	Depression (BGQ), complicated grief (PHQ‐9)	Significantly^d^ less frequently reporting of depression (17.3% vs. 21.6%; *p* < 0.001) and complicated grief (13.7% vs. 15.9%; *p* = 0.03); bereaved family members who participated in end‐of‐life discussions were less likely to develop depression or complicated grief; direct association between end‐of‐life discussions and reduction of depression and complicated grief in bereaved family members	80

Abbreviations: AAQ‐II, Acceptance and Action Questionnaire; BDI‐II, Beck Depression Inventory II; BGQ, Brief Grief Questionnaire; BSQ, Bereavement Service Questionnaire; CESD‐D, Centers for Epidemiological Studies‐Depression Scale; CGI‐I, Clinical Global Impressions‐Improvement Scale; CG‐QOL‐C, Caregiver Quality of Life Index (Cancer); CSNAT, Carer Support Needs Assessment Tool; DASS, Depression Anxiety Stress Scale; EPC, Early Palliative Care; FMS, Finding Meaning Scale; GAD‐7, Generalised Anxiety Disorder Screening Tool; GDS, Geriatric Depression Scale; GDS‐SF, Geriatric Depression Scale‐Short Form; GHQ, General Health Questionnaire; GHQ‐28, General Health Questionnaire; HADS, Hospital Anxiety and Depression Scale; HSS, Hope‐State Scale; ICG‐r, Inventory of Complicated Grief‐revised; IoDWL, Inventory of Daily Widowed Life; LDS, Leiden Detachment Scale; MBCB, Montgomery–Borgatta Caregiver Burden; PG‐12, Prolonged‐Loss Grief‐12; PG‐13, Prigerson Inventory of Complicated Grief; PGD, Prolonged grief disorder; PHQ‐9, Patient Health Questionnaire; PSCaDLCS, Perceived Self‐Care and Daily Living Competencies Scale; RCT, Randomised Controlled Study; SD, Standard Deviation; SDQ, Self‐Developed Questionnaire; SF12v2, Short Form Health Survey; TRIG, Texas Revised Inventory of Grief; VLQ, Valued Life Questionnaire; VOICES, Views of Informal Carers—Evaluation of Services.

^a^ Age range in parentheses.

^b^ Studies without a comparator (C) evaluated bereavement or palliative care services in general, not one specific intervention.

^c^ Mixed‐Methods Appraisal Tool.

^d^ Statistically significant.

The quality of the studies included was overall heterogeneous. Of the quantitative studies, nine were rated as high (41%), 11 as moderate (50%) and two as low‐quality (9%) (File S5). Low‐ or medium‐quality ratings were due to insufficient reporting, blinding, or unclear participant adherence to the study intervention. In addition, quantitative descriptive designs often had a high risk of non‐response bias, and the sample's representativeness of the target population was not always given. Nine (69%) qualitative studies were rated as high quality, and four (31%) as moderate quality. Based on the information provided, it was often unclear whether the findings were adequately derived from the data for qualitative studies. Multi‐ or mixed‐methods studies were rated as moderate (Wu et al. 2022; Hudson 2006), or low‐quality (McGinley and Waldrop 2020; Wittenberg‐Lyles et al. 2015) due to insufficient data integration.

### 3.3 Investigated Interventions and Outcomes

The broad definition of bereavement support led to the inclusion of a wide range of different interventions. Details are given in Table 3. Sixteen studies reported on pre‐loss interventions (Aoun, Ewing, et al. 2018; Cronfalk et al. 2009, 2010; Davis et al. 2020; Dionne‐Odom et al. 2016; Grande et al. 2017; Hudson et al. 2005, 2015; Magill 2009a, 2009b; Mooney et al. 2024; Reblin et al. 2019; Veerbeek et al. 2008; von Heymann‐Horan et al. 2018; Walsh et al. 2007; Yamaguchi et al. 2017), whereas 12 studies investigated post‐loss interventions delivered in bereavement (Aoun et al. 2017; Goebel et al. 2017; Levesque et al. 2023; Makarem et al. 2018; Nappa and Bjorkman‐Randstrom 2020; Nappa et al. 2016; Olsson et al. 2017; Petursdottir et al. 2020; Ramstad et al. 2023; Roberts and McGilloway 2008; Supiano et al. 2020; Wittenberg‐Lyles et al. 2015). A total of 8 studies provided evidence on general experiences with and perceptions of bereavement support services (Agnew et al. 2008; Hudson 2006; Kirby et al. 2018; Lundberg et al. 2013; Maze et al. 2022; McGinley and Waldrop 2020; McGrath et al. 2010; Tabler et al. 2015), and three studies entailed data on post‐loss interventions and general experiences (Wu et al. 2022; Aoun, Breen, et al. 2018; Muta et al. 2014).

**TABLE 3:** Overview of interventions.

Intervention	Intervention description	Duration and timing	Evidence synthesis category	Tier^a^
Practitioner‐facilitated but caregiver‐led needs and support assessment using the Carer Support Needs Assessment Tool (CSNAT‐Intervention) (Aoun, Ewing, et al. 2018)	Practitioner‐facilitated but carer‐led needs and support assessment with the 14 domains CSNAT (representing broad domains for which carers require support during caregiving at the end of life; two distinct groups: support that enables the family caregiver to care for the care recipient at home [seven domains] and direct support for the family caregiver in their caring role [seven domains]). Family caregivers identified domains where they needed more support by CSNAT self‐completion or jointly with the nurse. Followed by a conversation to determine individual needs, the caregiver's priorities were discussed with the nurse to agree on actions/solutions and a shared action plan.	At least two nurse visits and conversations 2 to 3 weeks apart in caregiving period before death.	Family‐focused interventions (single‐component)	n/a
Practitioner‐facilitated but caregiver‐led needs and support assessment using the Carer Support Needs Assessment Tool (CSNAT‐Intervention) (Grande et al. 2017)	Practitioner‐facilitated but carer‐led needs and support assessment with the 14 domains CSNAT (representing broad domains for which carers require support during caregiving at end of life; two distinct groups: support that enables the family caregiver to care for the care recipient at home [seven domains] and direct support for the family caregiver in their caring role [seven domains]), followed by discussion on ways to address the identified needs.	One discussion in caregiving period before death.	Family‐focused interventions (single‐component)	n/a
Soft tissue massage for caregivers (Cronfalk et al. 2009, 2010)	Hand or foot massage was carried out with slow strokes, light pressure and circling movements using a light‐scented vegetable oil.	2 weeks in end‐of‐life phase, first week 4 times, second week 5 times, each session approx. 25 min.	Family‐focused interventions (single‐component)	n/a
Music therapy during dying (Magill 2009a, 2009b)	Music therapy sessions for caregivers before the death of the patient.	End‐of‐life phase.	Family‐focused interventions (single‐component)	n/a
Symptom Care at Home intervention plus usual care during end‐of‐life (Mooney et al. 2024)	Daily evaluation of caregiver self‐reported symptoms using a telephone interactive voice response system, automated coaching messages (based on algorithms) tailored to the severity and pattern of the caregiving symptoms reported, automated information to hospice nurse, when symptoms over threshold.	From study enrollment (within first week of hospice) until 6 months or patient death.	Family‐focused interventions (single‐component)	n/a
Treatment as usual plus skill‐based booklet and telephone support during end‐of‐life (Davis et al. 2020)	Self‐help booklet with accompanying CD (psychoeducation and experiential mindfulness exercises) and one phone call after receiving the booklet to support understanding the material and personal application.	End‐of‐life phase. Duration overall 1–4 weeks. Booklet at inclusion in study (end‐of‐life phase), phone call 1–2 weeks after receiving the booklet (if carer became bereaved within this timeframe, call was delayed an additional 2 weeks).	Family‐focused interventions (single‐component)	1
Early palliative care support close to cancer diagnosis (Dionne‐Odom et al. 2016)	Early palliative care support for family caregivers (onset within 60 days of cancer diagnosis), guided by “Charting Your Course‐Caregiver Guidebook”. Session 1: Taking care role of caregiver, definition of palliative and supportive care, introduction to problem‐solving using the COPE (creativity, optimism, planning, expert information) framework. Session 2: Self‐care (e.g., healthy eating, exercise, relaxation) and effective partnering in patient symptom assessment and management. Session 3: Building a support team, decision‐making, decision support and advance care planning. If patient died during study: Bereavement call.	Start within 60 days after cancer diagnosis until patient's death or study end. Weekly calls for 3 weeks, at least monthly follow‐up calls until end of study or patient death (minimum follow‐up time: 24 weeks). Bereavement call: 2–4 weeks after patient death.	Family‐focused interventions (multi‐component)	n/a
Usual care and psychoeducational intervention during end‐of‐life (Hudson et al. 2005)	Nurse‐delivered: two home visits and a follow‐up phone call between the two visits. Caregiver guidebook and audiotape to provide caregivers with easy access to information related to typical aspects of caring for a dying person. Audiotape featured reflections from carers and incorporated self‐care strategies and structured relaxation exercises. Visit 1 (prepared with phone call): preparation for caregiving; caregivers were asked in advance to read sections 1 (preparation for caregiver role) and 2 (key aspects of care provision) of the guidebook and note questions before visit. Phone call: evaluation of plan developed in visit 1, reiterating importance of self‐care (section 3 of guidebook), identifying new issues, moving on to next section. Visit 2: evaluation of previous strategies, identifying new issues, preparation for dying phase (final section of guidebook).	End‐of‐life phase. First visit arranged 4 days after random assignment to intervention (end‐of‐life phase); phone call within 6 days after first visit; second visit arranged 6–8 days after phone call.	Family‐focused interventions (multi‐component)	n/a
Psychoeducational intervention plus standard care for caregivers during end‐of‐life (Hudson et al. 2015)	Tailored information and resources given to family caregivers to promote psychological well‐being. The primary written resource for caregivers was a guidebook, developed and tested in the pilot work that focused on preparation and management of the caregiver role with a strong focus on promoting psychological well‐being: (1) preparing caregivers for participation in the intervention, (2) assessing caregiver needs and preparing a care plan, (3) re‐assessing needs and evaluating the care plan and (4) assisting the family caregiver to prepare for their relative's death and to prepare for bereavement.	End‐of‐life phase. Duration of 4 weeks. One visit group: one face‐to‐face visit and three phone calls. Two visit group: two visits and two phone calls. First and final contact face‐to‐face.	Family‐focused interventions (multi‐component)	n/a
The Carer Advisor Intervention (Walsh et al. 2007)	Comprehensive assessment of domains of needs; discussing past, present and future issues; provision of advice, information and emotional support (personal visits or sometimes telephone calls) Focus on giving advice and support rather than acting on behalf of carers.	Before patient death. Six visits over 6 weeks.	Family‐focused interventions (multi‐component)	n/a
Transitional care (Domus intervention) (von Heymann‐Horan et al. 2018)	Accelerated transition from hospital‐based oncological treatment to specialised palliative care at home. (1) Home conference with palliative care team, municipal nursing service, general practitioner, project psychologist (within 5 working days of randomisation), (2) continuing national guideline‐ and needs‐based care for patients, (3) dyadic, manualised psychological intervention, beginning after home conference (two sessions within 1 month of randomisation, followed by monthly needs‐assessment and/or needs‐based sessions). One or two psychological closing sessions after patient's death.	Start within 5 working days of randomisation until patient death; home conference within 5 working days; patient care from randomisation until death; psychological intervention: two sessions within 1 month of randomisation, followed by monthly needs assessment and/or needs‐based sessions. One or two possible closing sessions for caregiver after patient's death.	Family‐focused interventions (multi‐component)	n/a
Liverpool Care Pathway (Veerbeek et al. 2008)	Multiprofessional document that incorporates evidence‐based practice and guidelines. Promoting adequate communication and support and giving relatives a brochure for bereavement Three sections: (1) Initial assessment and care, (2) Ongoing care, (3) Care after death.	Dying, last 3 days of life.	Communication interventions during dying	1
End‐of‐life discussions (Yamaguchi et al. 2017)	Discussions with physicians about the preferred place of care, resuscitation use of specialist palliative care services, transfer to another facility, any other topic.	End‐of‐life phase.	Communication interventions during dying	1
Communication of emotions (Reblin et al. 2019)	Hospice nurses supportive communication and exchange of positive emotions.	End‐of‐life phase.	Communication interventions during dying	1
Grief Coach (Text‐based grief support intervention) (Levesque et al. 2023)	Delivers text support, education, tips and reminders to grieving people and their friends and family who want to support them. Supporters' messages include practical tips on how to help the bereaved. 5 Strategies: (1) Psychoeducation, (2) acknowledgement and validation, (3) emotional and instrumental support, (4) coping skills, (5) grief work.	Bereavement phase. For 13 months following death, two messages per week.	Individual bereavement support interventions	n/a
Family Strengths‐Oriented Therapeutic Conversation (FSOTC) (Petursdottir et al. 2020)	Focuses on supporting the cognitive, affective and behavioural domains of the family member's illness experience: (1) Eliciting narratives about the pre‐loss and post‐loss experience; (2) asking therapeutic/interventive questions, emphasising the most pressing concerns and using therapeutic listening; (3) validating/acknowledging emotional responses; (4) assessing the need for specific information and recommendations regarding bereavement; and (5) the use of commendation/focusing on the strengths of the bereaved caregiver.	Bereavement phase. One session (60–90 min) and one possible follow‐up at 6 months.	Individual bereavement support interventions	n/a
Bereavement anniversary card (Goebel et al. 2017)	The bereavement card is sent out to the person listed as first contact exactly 1 year after the patient's death. Designed as a folded card with a nature motif, it consists of a handwritten salutation followed by a printed text denoting the recipient's specific relationship to the deceased and expressing condolences and sympathy.	Bereavement phase. 1 year after patient's death	Individual bereavement support interventions	n/a
Bereavement support services (Roberts and McGilloway 2008)	(1) Bereavement follow‐up contact by hospice staff made to a family member shortly after death; (2) Monthly Memorial Ceremony (MMC); (3) a Bereavement Information Evening (BIE), which entails a presentation by the hospice social worker on the bereavement process and the hospice bereavement services: and (4) a Volunteer Bereavement Support Service (VBSS), which comprises a listening service provided by trained volunteers under the supervision of the hospice social work team.	Bereavement phase.	Individual bereavement support interventions	1, 2
Bereavement contact (Makarem et al. 2018; Ramstad et al. 2023)	Bereavement contact from healthcare professionals.	Bereavement phase.	Individual bereavement support interventions	2
Face‐to‐face bereavement group (Nappa and Bjorkman‐Randstrom 2020; Nappa et al. 2016)	Bereavement group with three to eleven people. Each group meeting had a predefined theme: (1) Presentation of the methodology and introduction of the members in the group; (2) The time of the illness until death, (3) The time of death; (4) The time after death and the funeral; and (5) Depicting a metaphorical picture of the deceased and the significant other's life together.	Bereavement phase. One weekly session of 2 h for 5 weeks.	Group bereavement support interventions	2
Face‐to‐face bereavement support group following the loss of a parent to cancer (Olsson et al. 2017)	(1) Preintervention meeting with one professional group leader to inform about group format, meet leader and assess appropriateness of group support, (2) support group meeting with 5–10 participants for 2 h (group built on dual process model of grief, addressing loss oriented as well as restoration‐oriented issues).	Bereavement phase. Offered two to 8 weeks after death of parent. One weekly session for 10 weeks.	Group bereavement support interventions	2
Telehealth grief support group (Supiano et al. 2020)	Psychoeducation on grief, individual grief journeys, dealing with thoughts and emotions, coping strategies, communication strategies, being present with grief and planning for the life ahead.	Bereavement phase. One session per week for 8 weeks.	Group bereavement support interventions	2
Secret Facebook grief support group (Wittenberg‐Lyles et al. 2015)	Weekly posted educational link or material related to coping with grief, followed by a discussion. Twelve topics were chosen before the initiation of the group, based on common concerns with grieving caregivers. Hereafter, topics were selected based on the observation of the discussions by the moderators. Two social workers reviewed postings daily and responded when appropriate. Involvement by the social workers was triggered by notifications.	Bereavement phase. Group participation over 9 months.	Group bereavement support interventions	2

^a^ Public health model of bereavement support: Tier 1 = general level (e.g., leaflets, information about grief and bereavement, self‐help basic guidance); Tier 2 = selective level (e.g., non mental health specialist support in form of community groups, trained volunteers, clergy, chaplains); Tier 3 = indicated level (e. g. specialist mental health interventions) (Aoun et al. 2015; Killikelly et al. 2021; National Institute for Clinical Excellence 2004).

Grief response was the most frequently investigated quantitative bereavement outcome (*n* = 11, 27%), followed by anxiety (*n* = 9, 22%) and well‐being (*n* = 3, 7%). Details on quantitative outcomes are given in Table 4. Qualitative studies mainly explored the experiences of bereaved family members with specific interventions, such as support groups, bereavement contact or general bereavement support (*n* = 8, 67%).

**TABLE 4:** Overview of quantitative bereavement outcomes.

Core dimension (Harrop, Scott, et al. 2020)	Outcome	Measures	Results^a^
Negative mental & emotional state (*n* = 30)	Grief response (*n* = 11)^b^	TRIG (*n* = 3), PG‐13 (*n* = 2), BGQ (*n* = 2), AAG (*n* = 1), CBI (*n* = 1), ICG‐r (*n* = 1), PG‐12 (*n* = 1)	Significant effect (*n* = 3) (Grande et al. 2017; Supiano et al. 2020; Yamaguchi et al. 2017) No significant effect (*n* = 6) (Aoun, Ewing, et al. 2018; Davis et al. 2020; Dionne‐Odom et al. 2016; Nappa et al. 2016; Petursdottir et al. 2020; Walsh et al. 2007)
	Anxiety (*n* = 9)	HADS (*n* = 3), DASS (*n* = 1), DT (*n* = 1), GAD‐7 (*n* = 1), GHQ (*n* = 1), GHQ‐28 (*n* = 1), SC‐AS (*n* = 1)	Significant effect(*n* = 3) (Hudson et al. 2015; Petursdottir et al. 2020; von Heymann‐Horan et al. 2018) No significant effect (*n* = 6) (Davis et al. 2020; Grande et al. 2017; Hudson et al. 2005; Nappa et al. 2016; Walsh et al. 2007; Wittenberg‐Lyles et al. 2015)
	Depression (*n* = 7)	PHQ‐9 (*n* = 2), CES‐D (*n* = 1), DASS (*n* = 1), GDS‐SF (*n* = 1), HADS (*n* = 1), SC‐DS (*n* = 1)	Significant effect (*n* = 3) (Reblin et al. 2019; von Heymann‐Horan et al. 2018; Yamaguchi et al. 2017) No significant effect (*n* = 4) (Dionne‐Odom et al. 2016; Nappa et al. 2016; Petursdottir et al. 2020; Wittenberg‐Lyles et al. 2015)
	Detachment (*n* = 1)	Leiden Detachment Scale (*n* = 1)	Significant effect (*n* = 1) (Veerbeek et al. 2008)
	Distress (*n* = 1)	Depression Anxiety Scale (*n* = 1)	Significant effect (*n* = 1) (Walsh et al. 2007)
	Strain (*n* = 1)	Carer Strain Index (*n* = 1)	No significant effect (*n* = 1) (Petursdottir et al. 2020)
Quality of life and mental well‐being (*n* = 6)	Well‐being (*n* = 3)^c^	Short Form Health Survey (*n* = 2), self‐developed (*n* = 1)	Significant effect on psychological and physical well‐being (*n* = 1) (Grande et al. 2017) Significant effect on psychosocial well‐being (*n* = 1) (Olsson et al. 2017) No significant effect on physical and mental well‐being (*n* = 1) (Aoun, Ewing, et al. 2018) No significant effect on overall well‐being (*n* = 1) (Olsson et al. 2017)
	Bereavement adjustment (*n* = 1)	Various instruments (*n* = 1)	Significant effect (*n* = 1) (Mooney et al. 2024)
	Quality of life (*n* = 1)	Caregiver Quality of Life Index (Cancer) (*n* = 1)	No significant effect (*n* = 1) (Walsh et al. 2007)
	Reward (*n* = 1)	Rewards of Caregiving Scale (*n* = 1)	No significant effect on reward (*n* = 1) (Hudson et al. 2005)
Other (*n* = 7)	Service evaluation (*n* = 4)	Carer Support Needs Assessment tool (*n* = 1), self‐developed (*n* = 3)	Positive evaluation (*n* = 3) of bereavement card (Goebel et al. 2017), grief coach (Levesque et al. 2023), caregiver support during end‐of‐life (Aoun, Ewing, et al. 2018) Professionals had the highest proportions of perceived unhelpfulness (*n* = 1) (Aoun, Breen, et al. 2018)

	Bereavement contact (*n* = 2)	Bereavement Service Questionnaire (*n* = 1), self‐developed (*n* = 1)	Significant association between missing contact and depression and prolonged grief disorder (*n* = 1) (Ramstad et al. 2023) Considered helpful (Roberts and McGilloway 2008)
	Feeling of needs met or their support needs probed or listened to (*n* = 2)	Carer Support Needs Assessment tool (*n* = 2)	Significant effect (*n* = 1) (Aoun, Ewing, et al. 2018) No significant effect (*n* = 1) (Grande et al. 2017)
Communication evaluation (comprehensiveness of information) (*n* = 1)	Views of Informal Carers—Evaluation of Services (*n* = 1)	No significant effect (*n* = 1) (Veerbeek et al. 2008)

Abbreviations: AAG, Adult Attitude to Grief Scale; BGQ, Brief Grief Questionnaire; CBI, Core Bereavement Items; CES‐D, Centers for Epidemiological Studies – Depression Scale; DASS, Depression Anxiety Stress Scale; DT, Distress Thermometer; GAD‐7, Generalised Anxiety Disorder Screening Tool; GDS‐SF, Geriatric Depression Scale—Short Form; GHQ, General Health Questionnaire; GHQ‐28, General Health Questionnaire; HADS, Hospital Anxiety and Depression Scale; ICG‐r, Inventory of Complicated Grief—Revised; PG‐12, Pre‐loss Grief; PG‐13, Prolonged Grief Disorder; PHQ‐9, Patient Health Questionnaire; SC‐AS, Symptom Checklist‐92 Anxiety Subscale; SC‐DS, Symptom Checklist‐92 Depression Subscale; TRIG, Texas Revised Inventory of Grief.

^a^ All reported effects are in the direction of the favourable outcome.

^b^ Two studies used two instruments each to measure grief (Davis et al. 2020; Supiano et al. 2020).

^c^ One study provided an analysis for overall and psychosocial, physical, mental well‐being of the same instrument (Olsson et al. 2017).

### 3.4 Evidence Synthesis Results

**FIGURE 2:**
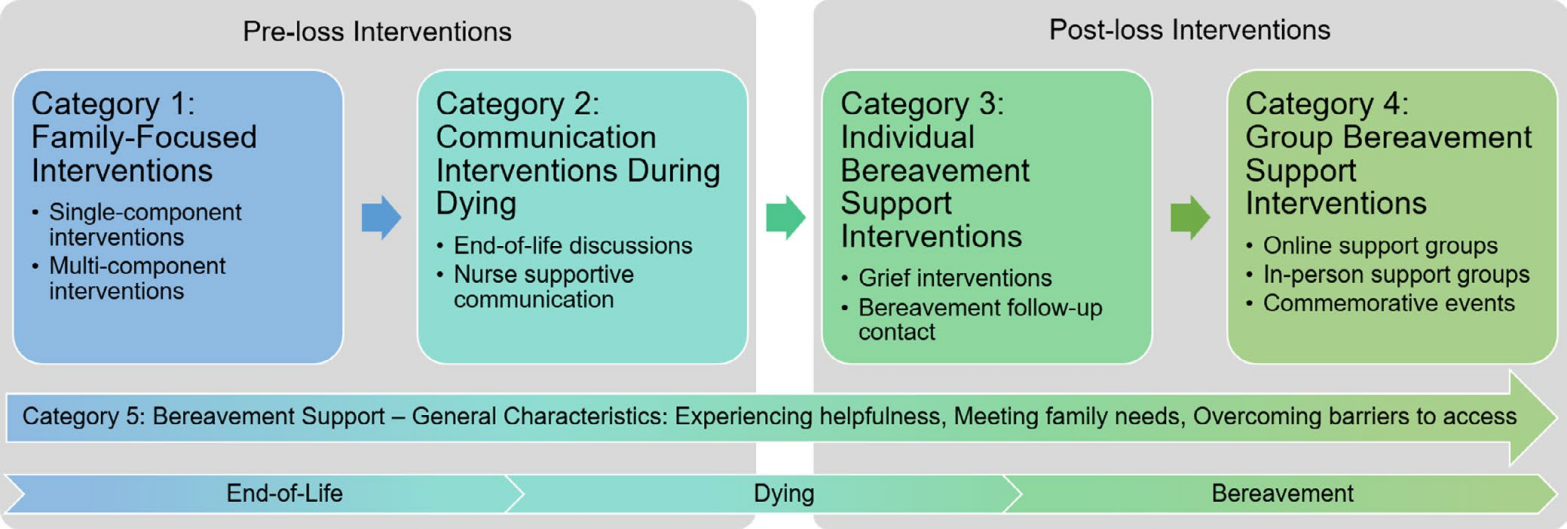
Evidence synthesis results.

Narrative evidence synthesis is organised into five *categories* (Figure 2): *family‐focused interventions and communication interventions* during dying (pre‐loss), *individual bereavement support interventions* and *group bereavement support interventions* (post‐loss). The final *category* emphasises the *general characteristics* of bereavement support.

#### 3.4.1 Pre‐Loss Interventions

##### 3.4.1.1 Family‐Focused Interventions

Thirteen studies investigated single‐ (Aoun, Ewing, et al. 2018; Cronfalk et al. 2009, 2010; Davis et al. 2020; Grande et al. 2017; Magill 2009a, 2009b; Mooney et al. 2024) or multi‐component (Dionne‐Odom et al. 2016; Hudson et al. 2005, 2015; von Heymann‐Horan et al. 2018; Walsh et al. 2007) family‐focused interventions during end‐of‐life in acute, community and home care, using mostly randomised controlled trials (RCTs) and qualitative research.

###### 3.4.1.1.1 Single‐Component Interventions

Eight moderate‐ to high‐quality studies assessed single‐component interventions (Aoun, Ewing, et al. 2018; Cronfalk et al. 2009, 2010; Davis et al. 2020; Grande et al. 2017; Magill 2009a, 2009b; Mooney et al. 2024), some of which showed a beneficial impact. For example, the Carer Support Needs Assessment Tool (CSNAT)‐Intervention is a practitioner‐facilitated but caregiver‐led comprehensive needs and support assessment. The CSNAT‐Intervention was investigated with a partly randomised stepped‐wedge trial (Grande et al. 2017), which resulted in reduced grief immediately after death (*p* = 0.038), and improved physical (*p* = 0.011) and psychological (*p* = 0.049) health 4 to 5 months post‐loss. A two‐group, pre‐post study of CSNAT (Aoun, Ewing, et al. 2018) reported no significant differences in early versus later grief levels and physical and mental well‐being post‐bereavement compared to pre‐bereavement. These CSNAT studies also differed concerning needs fulfilment. The stepped‐wedge trial did not find an intervention effect in contrast to the pre‐post study, where participants who received the CSNAT‐Intervention reported significantly higher needs fulfilment compared to those who did not (Cohen's *d* = 0.43, *p* < 0.001).

Other single‐component interventions showed promise. An RCT of a Symptom Care at Home Intervention found better 6‐month bereavement adjustment in the intervention group compared to the control group (partial *η* ^2^ = 0.245, *p* = 0.007) (Mooney et al. 2024). Music therapy (Magill 2009a, 2009b) and soft tissue massage for family members (Cronfalk et al. 2009, 2010) were perceived to improve well‐being during the close persons' end‐of‐life and in bereavement, promoting positive emotions and physical strength in qualitative studies.

###### 3.4.1.1.2 Multi‐Component Interventions

Five RCTs investigated multi‐component family‐focused interventions (Dionne‐Odom et al. 2016; Hudson et al. 2005, 2015; von Heymann‐Horan et al. 2018; Walsh et al. 2007) combining telephone support, home visits, and conferences, written and oral information, emotional or needs‐ and guideline‐based support, self‐care strategies, a dyadic manualised psychological intervention and relaxation exercises.

Except for two studies (Hudson et al. 2015; von Heymann‐Horan et al. 2018), none of these low‐ to moderate‐quality trials found an impact on depression, anxiety, grief, caregiver reward or strain, quality of life, or distress. The RCT by Hudson and colleagues (Hudson et al. 2015) identified a significantly stronger decrease in distress at 8 weeks post death compared to the baseline in the intervention group compared to the control group (Cohen's *d* = 0.58, *p* = 0.044). A transitional care intervention showed significantly decreased anxiety symptoms in the intervention group (Cohen's *d* = −0.19, *p* = 0.027) at each bereavement follow‐up compared to baseline (von Heymann‐Horan et al. 2018). It also found significantly decreased depressive symptoms in the intervention group at 2 weeks (Cohen's *d* = −0.42, *p* = 0.030) and 2 months post‐loss (Cohen's *d* = −0.37, *p* = 0.045).

##### 3.4.1.2 Communication Interventions During Dying

Three studies investigated communication interventions during dying (Reblin et al. 2019; Veerbeek et al. 2008; Yamaguchi et al. 2017) in institutional settings. End‐of‐life discussions were inversely associated with depression and complicated grief in a high‐quality survey study (Yamaguchi et al. 2017). Families with end‐of‐life discussions reported less frequent depression (17.3% vs. 21.6%, *p* < 0.001) and complicated grief (13.7% vs. 15.9%, *p* = 0.03) compared to those without (Yamaguchi et al. 2017). Use of the Liverpool Care Pathway, a standardised communication pathway, which was assessed with a moderate‐quality pre‐post‐test study, resulted in less difficulty with detachment from the deceased for families in the intervention period compared to the baseline period (*p* = 0.01) (Veerbeek et al. 2008). A moderate‐quality observational study found an independent positive association between hospice nurse supportive communication and positive emotion exchange during home visits with family members' depression during bereavement (Reblin et al. 2019).

#### 3.4.2 Post‐Loss Interventions

##### 3.4.2.1 Individual Bereavement Support Interventions

Seven moderate‐ to high‐quality studies investigated individual bereavement support interventions (Aoun et al. 2017; Goebel et al. 2017; Levesque et al. 2023; Makarem et al. 2018; Petursdottir et al. 2020; Ramstad et al. 2023; Roberts and McGilloway 2008), mainly in hospital and hospice settings (*n* = 6 quantitative, *n* = 1 qualitative).

###### 3.4.2.1.1 Grief Interventions

Family strength‐oriented therapeutic conversations resulted in significantly decreased anxiety (intervention vs. control, *p* = 0.048; over time, *p* = 0.005), while no effect on depressive symptoms up to 6 months post‐loss was found in a post‐test‐only study (Petursdottir et al. 2020). A significant main time effect was found for distress (*p* = 0.034), with the post hoc analysis indicating lower means at 6 months than after three and 5 months (*p* = 0.037 and *p* = 0.025, respectively). The online chatbot‐based text message support program *Grief Coach* was well received and appreciated by subscribers and considered somewhat more helpful by longer subscribers (6 months or longer) (Levesque et al. 2023).

###### 3.4.2.1.2 Bereavement Follow‐Up Contact

Follow‐up calls or contact offered from 3 weeks up to 6 months post‐loss by palliative care nurses and other professionals were examined with five moderate‐quality studies (one qualitative, three cross‐sectional surveys, one cohort) (Aoun et al. 2017; Goebel et al. 2017; Makarem et al. 2018; Ramstad et al. 2023; Roberts and McGilloway 2008). Bereaved family members experienced such follow‐ups as supportive for coping with loss. A prospective cohort study found that lack of follow‐up from the palliative team within 6 months post‐loss was significantly associated with prolonged grief disorder (adjusted OR: 3.2, 95% CI: 1.5–6.9) and depression symptoms (adjusted OR: 2.0, 95% CI: 1.3–3.2) after 3 years (Ramstad et al. 2023). Follow‐ups were not always perceived as necessary and were sometimes seen as a courtesy (Makarem et al. 2018). They were perceived as unhelpful if offered in a generic, impersonalised way (Aoun et al. 2017). In a survey study, almost all recipients experienced bereavement anniversary cards as supportive (Goebel et al. 2017). Family members reported feeling pleased, grateful and comforted. Nonetheless, for a minority, anniversaries come with sadness. While receiving an anniversary card was considered appropriate, families stressed that it needed to be personalised.

##### 3.4.2.2 Group Bereavement Support Interventions

Nine moderate‐ to high‐quality studies provided evidence on group bereavement support interventions (Wu et al. 2022; Aoun, Breen, et al. 2018; Muta et al. 2014; Nappa and Bjorkman‐Randstrom 2020; Nappa et al. 2016; Olsson et al. 2017; Roberts and McGilloway 2008; Supiano et al. 2020; Wittenberg‐Lyles et al. 2015) in settings like hospice, home, acute and community palliative care, or the general population (*n* = 5 quantitative, *n* = 2 qualitative, *n* = 2 multi‐method).

###### 3.4.2.2.1 Online Support Groups

Online grief support groups were evaluated in two studies with promising results (Supiano et al. 2020; Wittenberg‐Lyles et al. 2015). Telehealth group participants reported a decrease in grief (*p* < 0.0034) and complicated grief scores (*p* < 0.0026) in a one‐group pre‐post study (Supiano et al. 2020). Grief severity declined per week (*p* < 0.001) and grief improvement increased per week compared to study enrolment (*p* = 0.000) (Supiano et al. 2020). Facebook group participants reported non‐significant lower anxiety and depression mean scores at study completion compared to before joining the group in a one‐group pre‐post study (Wittenberg‐Lyles et al. 2015).

###### 3.4.2.2.2 In‐Person Support Group

A one‐group pre‐post study found improved psychosocial well‐being (*p* < 0.05), but no improvements in overall well‐being, feelings of loneliness, or ability to concentrate (Olsson et al. 2017). Another pre‐post study could not identify any effects on grief, anxiety and depression at 1 year for participants of support groups and those who were unable to participate (Nappa et al. 2016). Analysis of open‐ended survey questions suggests that families perceive support groups as beneficial for their well‐being and coping (Wu et al. 2022; Aoun, Breen, et al. 2018; Nappa and Bjorkman‐Randstrom 2020). The support groups helped individuals to maintain important relationships, facilitated reflection about themselves and others, and strengthened their self‐determination (Wu et al. 2022; Nappa and Bjorkman‐Randstrom 2020). Participants reported feeling socially integrated with a group sharing similar interests or concerns (Aoun, Breen, et al. 2018), which facilitated their expression of grief (Wu et al. 2022). While they did not resolve feelings of grief, support groups helped in creating meaning and managing different feelings of grief and promoted a more positive self‐perception, sense of understanding, and recognition (Nappa and Bjorkman‐Randstrom 2020).

###### 3.4.2.2.3 Commemorative Events

Bereaved families reported appreciating the reunion and interaction with staff and other bereaved families at memorial services hosted by a palliative care unit in a qualitative study (Muta et al. 2014). In a survey, over a quarter of respondents reported lacking the option of attending non‐religious monthly memorial ceremonies (Roberts and McGilloway 2008), yet most attendees (84%) were satisfied with a bereavement information evening.

#### 3.4.3 Bereavement Support—General Characteristics

In this section, we synthesised findings statements from 10 qualitative and one quantitative, mostly high‐quality studies on bereavement support in specialist palliative care across different settings into three themes (Wu et al. 2022; Agnew et al. 2008; Aoun, Breen, et al. 2018; Hudson 2006; Kirby et al. 2018; Lundberg et al. 2013; Maze et al. 2022; McGinley and Waldrop 2020; McGrath et al. 2010; Muta et al. 2014; Tabler et al. 2015). These studies address the general quality of care requirements for bereavement support.

##### 3.4.3.1 Experiencing Helpfulness

The qualitative findings spoke to the perceived helpfulness of palliative care nurses and other professionals and a general appreciation of available bereavement support in dealing with dying and loss (Wu et al. 2022; McGrath et al. 2010). Compassionate pre‐loss care by experienced professionals seemed to facilitate grieving (McGrath et al. 2010). However, one survey found that professional support (in contrast to family or social network support) had the highest proportion of perceived unhelpfulness (Aoun, Breen, et al. 2018), with qualitative data suggesting that inadequate interactions (e.g., lack of empathy or insensitivity) could explain these perceptions (Aoun, Breen, et al. 2018; Muta et al. 2014). Other families reported that palliative care nurses and other professionals play an important role in helping to deal with the situation (Wu et al. 2022; Agnew et al. 2008; Maze et al. 2022) by creating a therapeutic environment (Maze et al. 2022) or showing understanding (Wu et al. 2022). These interactions were connected to emotional experiences for families (Aoun, Breen, et al. 2018; Lundberg et al. 2013), such as warmth and comfort and a sense of value.

##### 3.4.3.2 Meeting Family Needs

Families express a need for comprehensive bereavement support systems, which start pre‐loss (Muta et al. 2014). Being prepared for death was important to families and supported by receiving information on the prognosis (Hudson 2006). Obstacles, such as little time, a preference to not prepare, or the need to fully focus on the close person, have also been described (Hudson 2006; Tabler et al. 2015). Clear and direct communication seems to be crucial for families across the dying–bereavement trajectory, not only between family, palliative care nurses and other professionals but also between different service providers involved in family care (Hudson 2006; McGinley and Waldrop 2020; Muta et al. 2014). Once bereaved, families need support with practical and organisational tasks, like assistance with funeral arrangements, estate and financial management (Kirby et al. 2018; McGrath et al. 2010). Bereavement support should be offered with a sense of timing to ensure it is accessible to the family when needed (Hudson 2006; Kirby et al. 2018; Tabler et al. 2015).

##### 3.4.3.3 Overcoming Barriers to Access

Several barriers to accessing bereavement support have been identified, such as not understanding bereavement services and doubts about its benefits (Agnew et al. 2008; Kirby et al. 2018). Further, the families described prejudices against bereavement and bereavement support (e.g., bereavement support for the weak, socially isolated, those not coping) (Agnew et al. 2008; Kirby et al. 2018), existing stereotypes (e.g., bereaved people supposed to be “sad”, deceased labelled as “loved ones”) and stigma in accepting professional support (Agnew et al. 2008; Kirby et al. 2018).

## 4 Discussion

This rapid mixed‐methods systematic review synthesises current evidence on family bereavement support in specialist adult palliative care. It reflects *general* and *selective* support that falls within the responsibility of palliative care nurses and other staff (Aoun et al. 2015; Killikelly et al. 2021; National Institute for Clinical Excellence 2004). A broad definition of bereavement support (Keegan et al. 2021) informed this review, encompassing pre‐ and post‐loss family interventions that assessed outcomes in bereavement. Therefore, a wide range of family support interventions in different settings met the inclusion criteria. About 40% of the studies investigated pre‐loss interventions, and about another 40% examined post‐loss interventions. The remaining studies investigated comprehensive, multi‐component palliative and bereavement services, often combining different interventions along the illness‐dying trajectory reflective of tiers one and two of the three‐tiered model of bereavement care (Aoun et al. 2015).

Our findings suggest that families benefit from bereavement support, which is consistent with previous evidence syntheses focusing on bereavement support in palliative care settings (Kustanti et al. 2021), hospital‐based (Boven et al. 2022; Green et al. 2022) and online bereavement support (Finucane et al. 2025; Robinson and Pond 2019) or for those bereaved by advanced illness (Harrop, Morgan, et al. 2020). We found that in specialist palliative care, pre‐loss communication and support (reflecting tier 1), as well as post‐loss follow‐up and support groups (reflecting tier 2), may improve family coping and bereavement outcomes. Included interventions potentially alleviate grief and mitigate adverse mental health outcomes such as depression, anxiety and prolonged grief. Still, the effects of the interventions were not always clearly evident or strong. Qualitative studies suggest that families perceive pre‐ and post‐loss interventions as beneficial and appreciate these services (Boven et al. 2022; Robinson and Pond 2019), expressing a need for comprehensive support starting before death and continuing into bereavement. Our review provides a specific knowledge base for supporting family members of persons dying while being cared for by specialist palliative care staff across the illness and bereavement trajectory. High‐quality and rigorous intervention research is now needed to improve understanding of the clinical effectiveness of bereavement support.

### 4.1 Pre‐Loss Interventions

The included studies yielded moderate‐level evidence on the benefit of pre‐loss interventions, such as family‐focused support and communication on various bereavement outcomes (e.g., depression, grief, anxiety, distress, health, quality of life and needs fulfilment). Our findings align with reviews investigating hospital and home‐based end‐of‐life support, which concluded that high‐level evidence on effectiveness was scarce, but interventions may improve family outcomes (Green et al. 2022; Becque et al. 2019).

Our findings suggest that a comprehensive needs assessment, such as the CSNAT‐Intervention, may lead to better needs fulfilment, benefit family well‐being and alleviate grief early in bereavement. This corroborates the findings of a previous review, which indicated that needs assessment could be one of the most effective components of nursing interventions for improving family outcomes in end‐of‐life home care, such as preparedness, competence, rewards and burden (Becque et al. 2019). However, in our review, the two studies investigating the CSNAT‐Intervention (Aoun, Ewing, et al. 2018; Grande et al. 2017) are inconclusive. Differences in study design, intervention delivery and context may have led to these divergent findings. Nevertheless, a comprehensive needs assessment may lead to better needs fulfilment. Research is needed to improve the understanding of the optimal timing, method and context for family needs assessment along the illness‐dying trajectory.

Other reviews suggest that families require clear, direct and thorough communication during their close persons' end‐of‐life, including honesty, openness and empathy (Engel et al. 2023; Anderson et al. 2019). Our findings indicate that families also require effective communication among the various service providers involved in family care throughout the dying and bereavement trajectory. Moreover, the data suggest that comprehensive and clear communication at the end of life may lead to improved bereavement outcomes, including lower levels of depression, grief and detachment. Communication is reported to play a critical role in preparing for death and alleviating difficult bereavement experiences for family members (Engel et al. 2023; Ryan et al. 2022; Chen et al. 2023; Nagelschmidt et al. 2021). However, emotional and cognitive barriers can hinder effective communication at this stage (Nagelschmidt et al. 2021). Our review highlights challenges such as limited time, a reluctance to prepare and the need to focus entirely on the close person, which health professionals should consider. Therefore, palliative care nurses should place greater emphasis on families, acknowledging them as care recipients whose communication and support needs must be consistently met, and they may consider more training in family care.

Our review underscores the necessity for more robust research to determine the impact of specific pre‐loss interventions on bereavement outcomes, including psychoeducation, needs assessments, comprehensive communication and support structures during the dying process.

### 4.2 Post‐Loss Interventions

Low‐level evidence on post‐loss family support indicates it could mitigate anxiety, distress and grief while enhancing overall well‐being. Still, our results do not allow for a definitive conclusion. Our review suggests that individual formats of bereavement support interventions tend to be more effective than group formats. This contradicts a meta‐analysis of bereavement support in palliative care settings, which found that group interventions were the more effective formats (Kustanti et al. 2021). Hence, we are unable to draw conclusions regarding the delivery format. For example, our qualitative results show that both delivery forms have beneficial impacts, which is consistent with other reviews (Harrop, Morgan, et al. 2020; Robinson and Pond 2019). These have identified a positive impact of both individual and group bereavement support on *loss and grief resolution, sense of mastery and moving ahead*, and *social support* in various settings for people bereaved through advanced illness (Harrop, Morgan, et al. 2020). There are positive attributes of online peer support groups for bereaved persons, for example, emotional support, sharing information and remembrance (Robinson and Pond 2019). Our results highlight these positive impacts on coping with loss and well‐being for families. A recent review noted only marginal effects of in‐person bereavement groups on grief and depressive symptoms (Maass et al. 2022). In contrast, in our review, an experimental, uncontrolled study on a telehealth support group found a significant improvement in grief symptoms (Supiano et al. 2020). We also found that online bereavement groups may be more promising than in‐person groups in mitigating adverse mental health outcomes, but the evidence base in our review is small and insufficient. Recently, Finucane and colleagues (Finucane et al. 2025) examined both online individual and group bereavement support interventions and reported favourable effects, for example, reduced isolation, access to coping advice and normalisation of loss responses.

Given the somewhat contradictory and unclear results, comparative research is needed. Future research should investigate the effectiveness of bereavement support groups according to delivery formats, focusing on outcomes relevant to families and who benefits the most from which delivery mode.

Our findings suggest that individual follow‐up support three to 6 months after bereavement is valued by families and potentially beneficial for them, and one longitudinal study identified adverse effects on mental health when interactions and support from palliative staff were absent following the death of the close other (Ramstad et al. 2023). Not all families require such bereavement support (Makarem et al. 2018) as noted previously (Boven et al. 2022), which emphasised the value of a three‐tiered, needs‐oriented, health‐promoting bereavement support model (Lichtenthal et al. 2024; Aoun et al. 2015). Our results suggest that nurses and other palliative care staff should continuously assess the needs of bereaved families over time and provide individualised, targeted follow‐up within 6 months post‐loss. However, how bereaved families' needs and risks for adverse bereavement outcomes should be assessed in a culturally sensitive manner is still unclear (Hilberdink et al. 2023). Further research is needed to understand the optimal form and timing of both assessment and follow‐up care, as well as who benefits most. Experimental designs (e.g., RCTs) are necessary to evaluate the effectiveness of follow‐up programmes.

### 4.3 Characteristics of Bereavement Support

We have identified several key characteristics that bereavement support services in specialist palliative care settings should consider: the importance of high‐quality, empathic service provision, the necessity of meeting individual needs and the need to remove barriers to access. Similar results on the quality of services and access barriers (e.g., limited or lack of support, discomfort asking for help) have also been found for other settings (Aoun et al. 2015; Green et al. 2022; Harrop et al. 2021).

Our research indicates that there are barriers, such as stigma related to bereavement support and lack of understanding of services, which should be tackled on multiple levels. First, practitioners should recognise these challenges and address them with empathy on an individual basis. Second, they should be addressed at the system level: educating the public about bereavement and support services could help reduce stigma (Lichtenthal et al. 2024; Breen et al. 2022). To promote death and grief literacy (Breen et al. 2022) and accessibility to services, community‐based bereavement support, such as the compassionate community approach (Aoun, Breen, et al. 2018; Breen et al. 2022), has been recommended. Our review shows that families seek personalised and comprehensive support, with very few finding it unnecessary or unhelpful. Families need support with practical tasks (Kirby et al. 2018; McGrath et al. 2010), which has been previously found to be a high burden for them, requiring staff attention. Our findings underpin a transitional, needs‐ and risk‐based approach to bereavement support (Lichtenthal et al. 2024; Killikelly et al. 2021; Boven et al. 2022). The call for risk and needs‐oriented bereavement support is not new (Aoun et al. 2015; National Institute for Clinical Excellence 2004; Breen et al. 2014), and has recently been renewed (Lichtenthal et al. 2024). To tailor bereavement support interventions to families' needs, they should begin before death, continue into bereavement and be embedded in comprehensive family‐focused support services. Additionally, this review identifies barriers to accessing bereavement support in palliative care and adds to existing knowledge in this area (Harrop et al. 2021).

### 4.4 Methodological Considerations

The included studies showed overall heterogeneous quality. Inadequate reporting was often a problem. This could be addressed by strict adherence to available reporting guidelines. Other challenges, such as the risk of non‐response bias and unclear sample representativeness of the target population, reflect common challenges in palliative care and bereavement research involving families (Walshe et al. 2024; Cruz‐Oliver et al. 2023). Future studies should follow evidence‐based recommendations for conducting research with families (Cruz‐Oliver et al. 2023; Hudson et al. 2023; Mitchell et al. 2024).

The studies mainly included assessments of grief and adverse mental health outcomes, such as anxiety, depression and prolonged grief disorder, using various instruments, which makes comparing their results challenging. Coping and well‐being, proposed by Harrop and colleagues (Harrop, Scott, et al. 2020) as core bereavement outcomes, have been less well explored and should be included in future evaluations of bereavement support services. The development of new, valid instruments or the consistent application of existing ones will facilitate the comparison across individual studies in future evidence synthesis work.

Conducting research involving family members in palliative care and bereavement settings poses well‐documented challenges (Walshe et al. 2024; Cruz‐Oliver et al. 2023; Hudson et al. 2023; Payne et al. 2022) due to the vulnerability and burden experienced by families (Breen et al. 2018; Holtslander et al. 2017). Proposed strategies for conducting high‐quality and inclusive research with families include a clear definition of “family carer”, public and patient involvement, commitment to equity and diversity, early staff involvement, attention to study burden, appropriate compensation and telephone recruitment (Cruz‐Oliver et al. 2023; Hudson et al. 2023; Mitchell et al. 2024). Additionally, new research methods should be explored to minimise the burden of research participation on families and to improve outreach to marginalised groups. Exploring decentralised trials (Jean‐Louis and Seixas 2024) that leverage digital elements could be beneficial, especially for engaging marginalised populations and enhancing participant diversity.

Family support interventions are often complex (Danford et al. 2024). They involve multiple components, target a range of behaviours and settings and must be adaptable to different healthcare contexts (Skivington et al. 2021; Jolles et al. 2024). Rigorous programs of research that combine different methodologies are needed to disentangle the mechanisms of impact and generate data on their effectiveness (Minary et al. 2019). Implementation science approaches should be applied to facilitate successful intervention implementation in real‐world settings and to create valuable insights into implementation processes (Breen and Moullin 2022; Kohler et al. 2025). The use of experimental and mixed‐methods designs is of value and will be important to substantiate the evidence base for pre‐ and post‐loss *general* and *selective* family support interventions in specialist palliative care (Walshe et al. 2024). The potential of mixed‐methods design was not fully exploited in the included studies: neither of the two mixed‐methods studies effectively integrated quantitative and qualitative data, nor did they provide a rationale for using mixed‐methods, despite these being considered core quality criteria (Hirose and Creswell 2023; Fàbregues et al. 2024). Future high‐quality mixed‐methods studies need to employ rigorous designs (Creswell and Creswell 2018) and adhere to core quality criteria (Hirose and Creswell 2023), such as integrating qualitative and quantitative data to a sufficient extent (Johnson et al. 2019).

Future bereavement support intervention research should draw on state‐of‐the‐art recommendations and guidance, such as the MRC framework for developing and evaluating complex interventions (Skivington et al. 2021). The development of new interventions should be guided by the three‐tiered public health model (Aoun et al. 2015; Killikelly et al. 2021; National Institute for Clinical Excellence 2004) to design effective interventions for *general* (tier 1) and *selective* (tier 2) support in specialised palliative care. A clear categorisation by tier may enhance the comparability of new interventions, facilitate future evidence syntheses and ensure that interventions are tailored to risks and needs.

### 4.5 Limitations and Strengths

The use of a rigorous mixed‐methods systematic review methodology facilitated the transparency and validity of the findings. However, several limitations exist. First, we undertook a rapid review, which means that some steps, like screening or quality assessment of studies, were not fully completed by two researchers. This could have introduced bias. Second, despite a systematic search in different electronic databases and a systematic screening process, it is possible that eligible studies were overlooked. Our exclusion criteria were such that we could not include grey literature or studies published in languages other than English, French, or German, which may have introduced selection and language bias. Most studies were conducted in Western countries involving informal caregivers and family members (almost one‐fifth focused on families of cancer patients), which limits the transferability of the findings to other cultures and conditions. Third, we employed a broad definition of bereavement support, which resulted in the inclusion of diverse interventions and various study designs. This limits the ability to determine the effectiveness of specific interventions or their components. In addition, many studies have examined multilevel or multi‐component intervention programs, making it difficult to differentiate between *general* and *selective* levels of the three‐tiered model. Fourth, the quality of the studies included was mixed, and we included low‐quality studies, which we accounted for in the interpretation of the results. Nonetheless, the results should be interpreted with caution. Despite these limitations, this review provides important insights into pre‐ and post‐loss family support within specialist adult palliative care services and may inform the redesign or development of bereavement support services for families who have experienced the loss of a close person.

## 5 Conclusion

Research in the field of bereavement support in specialist adult palliative care is characterised by heterogeneity in study design, methodological quality, types of bereavement support and interventions and outcomes assessed. Pre‐ and post‐loss interventions have received similar research attention, with most focusing primarily on grief and adverse mental health outcomes and less on family coping or well‐being outcomes. Our findings indicate that pre‐loss family‐focused support and comprehensive communication address needs and may alleviate grief and adverse mental health outcomes during bereavement. Post‐loss interventions may decrease anxiety, distress and grief while enhancing well‐being. Bereavement support should be tailored to the needs of families and offered to those who wish to receive it. High‐quality, comprehensive, multicentre research programmes that employ diverse, innovative and rigorous methods that investigate a broader range of health, coping and well‐being outcomes are now essential to substantiate the evolving evidence base. Palliative care services should provide comprehensive, tailored, family‐focused support throughout the entire illness‐dying‐bereavement trajectory, ensuring accessibility across health systems. Community‐based support structures should be developed to promote death and grief literacy and to ensure equity and access by removing barriers to bereavement support.

## Author Contributions

T.S. and R.N. designed the work. T.S. acquired the data. T.S., M.R. and R.N. analysed it, and all authors interpreted the data. T.S. and R.N. drafted the manuscript, and M.R. and M.K. revised it critically for intellectual content. All authors approved the final version of this manuscript and have participated sufficiently in the work to take public responsibility for appropriate portions of the content.

## Ethics Statement

The authors have nothing to report.

## Consent

The authors have nothing to report.

## Conflicts of Interest

The authors declare no conflicts of interest.

## Supporting information


**File S1:** Search strings Embase, CINAHL, PsycINFO, Cochrane library.
**File S2:** Endnote screening details.
**File S3:** Modified Cochrane quantitative data collection form.
**File S4:** Excluded full texts with reason.
**File S5:** Quality assessment.

## Data Availability

The data that support the findings of this study are openly available in the public domain. References to the original study publications are available within this manuscript. The full search strategy used in the present review can be found in the Supporting information.
