# Supplementary material for: Family Bereavement Support Interventions in Specialist Adult Palliative Care: A Rapid Mixed‐Methods Systematic Review
Source: J Adv Nurs. 2025 Sep 5;82(5):4682–713. doi: 10.1111/jan.70193 (PMC13069219; doi:10.1111/jan.70193)
Supplement: Supplementary file 1 — File S1: Search strings Embase, CINAHL, PsycINFO, Cochrane library. File S2: Endnote screening details. File S3: Modified Cochrane quantitative data collection form. File S4: Excluded full texts with reason. File S5: Quality assessment. [file JAN-82-4682-s001.docx]

**Supplementary files**

**Family bereavement support interventions in specialist adult palliative care: A rapid mixed-methods systematic review.**

Torsten Schwalbach^1,2^, Marco Riguzzi^1,2^, Myrta Kohler^1,2^, Rahel Naef^1,2^

^1^Institute for Implementation Science in Health Care, University of Zurich Faculty of Medicine, Zurich, Switzerland

^2^Center of Clinical Nursing Science, University Hospital Zurich, Zurich, Switzerland

[**Supplementary file 1: Search strings Embase, CINAHL, PsycINFO, Cochrane Library** 2](#_Toc194051046)

[**1.1 Embase** 2](#_Toc194051047)

[**1.2 CINAHL via EBSCOhost** 3](#_Toc194051048)

[**1.3. PsycINFO via EBSCOhost** 5](#_Toc194051049)

[**1.4. Cochrane Library** 6](#_Toc194051050)

[**Supplementary file 2: Endnote Screening details** 7](#_Toc194051051)

[**Supplementary file 3: Modified Cochrane quantitative data collection form** 8](#_Toc194051052)

[**Supplementary file 4: Excluded full texts with reason** 9](#_Toc194051053)

[**Supplementary file 5: Quality assessment** 30](#_Toc194051054)

[**5.1 Quantitative studies** 30](#_Toc194051055)

[**5.2 Qualitative Studies** 35](#_Toc194051056)

[**5.3 Mixed Methods Studies** 37](#_Toc194051057)

[**5.4 Multi Methods Studies** 39](#_Toc194051058)

# **Supplementary file 1: Search strings Embase, CINAHL, PsycINFO, Cochrane Library**

## **1.1 Embase**

| **No.** | **Query** |
| --- | --- |
| #9 | #1 AND #2 AND #3 NOT ([animals]/lim NOT [humans]/lim) NOT [conference abstract]/lim AND ([english]/lim OR [french]/lim OR [german]/lim) NOT (([child]/lim OR [adolescent]/lim) NOT [adult]/lim) NOT (('pediatric':ti,kw OR 'childhood':ti,kw OR 'children':ti,kw OR 'neonatal':ti,kw OR 'perinatal':ti,kw) NOT ('adult children*':ti,kw OR 'adult child*':ti,kw)) |
| #8 | #1 AND #2 AND #3 NOT ([animals]/lim NOT [humans]/lim) NOT [conference abstract]/lim AND ([english]/lim OR [french]/lim OR [german]/lim) NOT (([child]/lim OR [adolescent]/lim) NOT [adult]/lim) |
| #7 | #1 AND #2 AND #3 NOT ([animals]/lim NOT [humans]/lim) NOT [conference abstract]/lim AND ([english]/lim OR [french]/lim OR [german]/lim) |
| #6 | #1 AND #2 AND #3 NOT ([animals]/lim NOT [humans]/lim) NOT [conference abstract]/lim |
| #5 | #1 AND #2 AND #3 NOT ([animals]/lim NOT [humans]/lim) |
| #4 | #1 AND #2 AND #3 |
| #3 | 'hospice'/exp OR 'palliative therapy'/exp OR 'palliative nursing'/exp OR 'terminal care'/exp OR palliation:ti,ab,kw OR (((palliative OR 'end of life' OR eol OR hospice* OR terminal) NEAR/3 (treatment* OR nursing* OR therap* OR consultation* OR service* OR care OR unit* OR medic* OR situation*)):ti,ab,kw) |
| #2 | 'bereavement'/exp OR 'bereavement support'/exp OR 'bereavement counseling'/exp OR 'bereavement care*':ti,ab,kw OR 'bereavement practi*':ti,ab,kw OR 'bereavement follow-up*':ti,ab,kw OR (((bereavement OR death OR 'end of life' OR eol OR conclusion OR loss OR grief OR postdeath) NEAR/3 (support* OR intervention* OR service* OR program* OR counsel*)):ti,ab,kw) |
| #1 | 'grief'/exp AND (people:ti,ab,kw OR person*:ti,ab,kw OR famil*:ti,ab,kw OR families:ti,ab,kw OR familial:ti,ab,kw OR 'family member*':ti,ab,kw OR carer*:ti,ab,kw OR caregiv*:ti,ab,kw OR relative*:ti,ab,kw OR relation:ti,ab,kw OR relations:ti,ab,kw OR spouse*:ti,ab,kw OR partner*:ti,ab,kw OR husband*:ti,ab,kw OR widower*:ti,ab,kw OR wife:ti,ab,kw OR wives:ti,ab,kw OR widow*:ti,ab,kw OR volunteer*:ti,ab,kw OR 'close other*':ti,ab,kw OR 'next of kin*':ti,ab,kw) OR 'family'/exp OR 'caregiver'/exp OR bereaved:ti,ab,kw OR 'bereaved person*':ti,ab,kw OR (((bereaving OR grief OR grieving OR mourning OR mournful OR coping) NEAR/3 (people OR person* OR family OR families OR familial OR carer* OR caregiv* OR relative* OR relation OR relations OR spouse* OR partner* OR husband* OR widower* OR wife OR wives OR widow* OR volunteer*)):ti,ab,kw) |

## **1.2 CINAHL via EBSCOhost**

| **#** | **Query** | **Limiters/Expanders** |
| --- | --- | --- |
| S7 | S6 NOT (TI ('pediatric' OR 'childhood' OR 'children' OR 'neonatal' OR 'perinatal') NOT TI ('adult children*' OR 'adult child*')) | Limiters - Language: English, French, German |
| S6 | S5 NOT ((MH "child+" OR MH adolescent) NOT MH "adult+") |  |
| S5 | S4 NOT (MH "animals" NOT MH "human") |  |
| S4 | S1 AND S2 AND S3 |  |
| S3 | (MH "Hospice and Palliative Nursing") OR (MH "Hospice Care") OR (MH "Hospices") OR (MH "Palliative Care") OR (MH "Palliative Medicine") OR (MH "Terminal Care") OR TI (palliation OR ((palliative OR 'end of life' OR eol OR hospice* OR terminal) N3 (treatment* OR nursing* OR therap* OR consultation* OR service* OR care OR unit* OR medic* OR situation*))) OR AB (palliation OR ((palliative OR 'end of life' OR eol OR hospice* OR terminal) N3 (treatment* OR nursing* OR therap* OR consultation* OR service* OR care OR unit* OR medic* OR situation*))) |  |
| S2 | ((MH "Bereavement+") AND ((MH "Support, Psychosocial+") OR (MH "Death Counseling") OR (MH "Support, Social+") OR (MH "Psychosocial Intervention") OR (MH "Counseling") OR TI (support* OR intervention* OR service* OR program* OR counsel*) OR AB (support* OR intervention* OR service* OR program* OR counsel*))) OR TI ('bereavement care*' OR 'bereavement practi*' OR 'bereavement follow-up*' OR ((bereavement OR death OR 'end of life' OR eol OR conclusion OR loss OR grief OR postdeath) N3 (support* OR intervention* OR service* OR program* OR counsel*))) OR AB ('bereavement care*' OR 'bereavement practi*' OR 'bereavement follow-up*' OR ((bereavement OR death OR 'end of life' OR eol OR conclusion OR loss OR grief OR postdeath) N3 (support* OR intervention* OR service* OR program* OR counsel*))) |  |
| S1 | ((MH "Grief+") AND (TI (people OR person* OR famil* OR families OR familial OR 'family member*' OR 'family members' OR carer* OR caregiv* OR relative* OR relation OR relations OR spouse* OR partner* OR husband* OR widower* OR wife OR wives OR widow* OR volunteer* OR 'close other*' OR 'next of kin*') OR AB (people OR person* OR famil* OR families OR familial OR 'family member*' OR 'family members' OR carer* OR caregiv* OR relative* OR relation OR relations OR spouse* OR partner* OR husband* OR widower* OR wife OR wives OR widow* OR volunteer* OR 'close other*' OR 'next of kin*'))) OR (MH "Family+") OR (MH "Caregivers") OR TI (bereaved OR 'bereaved person*' OR ((bereaving OR grief OR grieving OR mourning OR mournful OR coping) N3 (people OR person* OR family OR families OR familial OR carer* OR caregiv* OR relative* OR relation OR relations OR spouse* OR partner* OR husband* OR widower* OR wife OR wives OR widow* OR volunteer*))) OR AB (bereaved OR 'bereaved person*' OR ((bereaving OR grief OR grieving OR mourning OR mournful OR coping) N3 (people OR person* OR family OR families OR familial OR carer* OR caregiv* OR relative* OR relation OR relations OR spouse* OR partner* OR husband* OR widower* OR wife OR wives OR widow* OR volunteer*))) |  |

## **1.3. PsycINFO via EBSCOhost**

| **#** | **Query** | **Limiters/Expanders** |
| --- | --- | --- |
| S5 | S4 NOT ( TI ('pediatric' OR 'childhood' OR 'children' OR 'neonatal' OR 'perinatal') NOT TI ('adult children*' OR 'adult child*')) | Limiters - Language: English, French, German |
| S4 | S1 AND S2 AND S3 |  |
| S3 | DE "Hospice" OR DE "Terminally Ill Patients" OR DE "Palliative Care" OR DE "Terminal Cancer" OR TI (palliation OR ((palliative OR 'end of life' OR eol OR hospice* OR terminal) N3 (treatment* OR nursing* OR therap* OR consultation* OR service* OR care OR unit* OR medic* OR situation*))) OR AB (palliation OR ((palliative OR 'end of life' OR eol OR hospice* OR terminal) N3 (treatment* OR nursing* OR therap* OR consultation* OR service* OR care OR unit* OR medic* OR situation*))) |  |
| S2 | DE "Grief Counseling" OR DE "Death Rites" OR ((DE "Bereavement" OR DE "Grief" OR DE "Traumatic Loss" OR DE "Death and Dying" OR DE "Parental Death" OR DE "Partner Death") AND (DE "Social Support" OR DE "Support Groups" OR DE "Family Intervention" OR DE "Group Intervention" OR TI (support* OR intervention* OR service* OR program* OR counsel*) OR AB (support* OR intervention* OR service* OR program* OR counsel*))) OR TI ('bereavement care*' OR 'bereavement practi*' OR 'bereavement follow-up*' OR ((bereavement OR death OR 'end of life' OR eol OR conclusion OR loss OR grief OR postdeath) N3 (support* OR intervention* OR service* OR program* OR counsel*))) OR AB ('bereavement care*' OR 'bereavement practi*' OR 'bereavement follow-up*' OR ((bereavement OR death OR 'end of life' OR eol OR conclusion OR loss OR grief OR postdeath) N3 (support* OR intervention* OR service* OR program* OR counsel*))) |  |
| S1 | ((DE "Grief" OR DE "Prolonged Grief Disorder" OR DE "Traumatic Loss") AND (TI (people OR person* OR famil* OR families OR familial OR 'family member*' OR 'family members' OR carer* OR caregiv* OR relative* OR relation OR relations OR spouse* OR partner* OR husband* OR widower* OR wife OR wives OR widow* OR volunteer* OR 'close other*' OR 'next of kin*') OR AB (people OR person* OR famil* OR families OR familial OR 'family member*' OR 'family members' OR carer* OR caregiv* OR relative* OR relation OR relations OR spouse* OR partner* OR husband* OR widower* OR wife OR wives OR widow* OR volunteer* OR 'close other*' OR 'next of kin*'))) OR DE "Widowers" OR DE "Widows" OR DE "Family Members" OR DE "Adopted Children" OR DE "Adult Offspring" OR DE "Biological Family" OR DE "Cousins" OR DE "Daughters" OR DE "Foster Children" OR DE "Grandchildren" OR DE "Grandparents" OR DE "Inlaws" OR DE "Only Children" OR DE "Orphans" OR DE "Parents" OR DE "Siblings" OR DE "Sons" OR DE "Spouses" OR DE "Stepchildren" OR DE "Caregivers" OR TI (bereaved OR 'bereaved person*' OR ((bereaving OR grief OR grieving OR mourning OR mournful OR coping) N3 (people OR person* OR family OR families OR familial OR carer* OR caregiv* OR relative* OR relation OR relations OR spouse* OR partner* OR husband* OR widower* OR wife OR wives OR widow* OR volunteer*))) OR AB (bereaved OR 'bereaved person*' OR ((bereaving OR grief OR grieving OR mourning OR mournful OR coping) N3 (people OR person* OR family OR families OR familial OR carer* OR caregiv* OR relative* OR relation OR relations OR spouse* OR partner* OR husband* OR widower* OR wife OR wives OR widow* OR volunteer*))) |  |

## **1.4. Cochrane Library**

| **#** | **Query** |
| --- | --- |
| 1 | bereaved:ti,ab,kw OR bereaved NEXT person*:ti,ab,kw OR (((bereaving OR grief OR grieving OR mourning OR mournful OR coping) NEAR/3 (people OR person* OR family OR families OR familial OR carer* OR caregiv* OR relative* OR relation OR relations OR spouse* OR partner* OR husband* OR widower* OR wife OR wives OR widow* OR volunteer*)):ti,ab,kw) |
| 2 | (bereavement NEXT care*):ti,ab,kw OR (bereavement NEXT practi*):ti,ab,kw OR (bereavement NEXT follow-up*):ti,ab,kw OR (((bereavement OR death OR "end of life" OR eol OR conclusion OR loss OR grief OR postdeath) NEAR/3 (support* OR intervention* OR service* OR program* OR counsel*)):ti,ab,kw) |
| 3 | palliation:ti,ab,kw OR (((palliative OR "end of life" OR eol OR hospice* OR terminal) NEAR/3 (treatment* OR nursing* OR therap* OR consultation* OR service* OR care OR unit* OR medic* OR situation*)):ti,ab,kw) |
| 4 | #1 AND #2 AND #3 |
| 5 | #4 NOT (pediatric OR childhood OR children OR neonatal OR perinatal):ti,kw |

# **Supplementary file 2: Endnote Screening details**

| **Advanced Search option** | |
| --- | --- |
| **Field** | **Keyword** |
| Title | Intensive Care Unit |
| Title | ICU |
| Title | Intensive Care |
| Title | Critical Care Nurse |
| Title | Critical Care Nurses |
| Title | Pediatric |
| Title | Pediatrics |
| Title | Child |
| Title | Children |
| Title | Adolescent |
| Title | Adolescents |
| Title | Emergency Department |
| Title | Emergency Departments |
| Title | Emergency Room |
| Title | Emergency |
| Title | Violence |
| Title | Violent |
| Title | Suicide |
| Title | accident |
| Title | disaster |
| Title | psychotherapy |
| Title | grief therapy |
| Title | interpersonal therapy |
| Title | interpersonal therapies |
| Title | cognitive behavior therapy |
| Title | cognitive behavior therapies |
| Title | cognitive behaviour therapy |
| Title | cognitive behaviour therapies |
| Title | review |
| Title | reviews |
| Title | meta analysis |
| Title | meta-analysis |
| Title | synthesis |
| Title | conference |
| Title | conferences |

# **Supplementary file 3: Modified Cochrane quantitative data collection form**

|  | **Study 1** | **Study 2** | **…** |
| --- | --- | --- | --- |
| **Date form completed** |  |  |  |
| **Review ID**  (First Author + Year) |  |  |  |
| **Study ID**  (First Author + Year from main report of the study) |  |  |  |
| **Reference Citation** |  |  |  |
| **Country** |  |  |  |
| **Research Question(s)** |  |  |  |
| **Design**  (RCT, quasi-randomized CT, controlled before-after, interrupted time series, other), Methods used |  |  |  |
| **Setting/Context** |  |  |  |
| **Participants characteristics**  (age [mean, range], sex, race/ethnicity) |  |  |  |
| **Intervention**  (description, components, theoretical foundation, duration of treatment period, timing [e.g. frequency, duration of each episode; session and times], delivery, provider) |  |  |  |
| **Comparison**  (description, duration of treatment period, timing [e.g. frequency, duration of each episode; session and times], delivery, provider) |  |  |  |
| **Outcomes measured**  (time points measured, time points reported, outcome definition, person measuring, unit of measurement, scales: upper and lower limits, outcome/tool validated? |  |  |  |
| **Measured effect size (Unit) and Power**  (e.g., power & sample size calculation, level of power achieved) |  |  |  |
| **Description of main results** |  |  |  |
| **Key Conclusions of Authors** |  |  |  |
| **Notes** |  |  |  |

# **Supplementary file 4: Excluded full texts with reason**

| **Authors** | **Title** | **Published** | **Journal** | **DOI** | **Exclusion reason** |
| --- | --- | --- | --- | --- | --- |
| Abernethy, Amy P.; Currow, David C.; Fazekas, Belinda S.; Luszcz, Mary A.; Wheeler, Jane L.; Kuchibhatla, Maragatha | Specialized palliative care services are associated with improved short- and long-term caregiver outcomes | 2008 | Supportive care in cancer : official journal of the Multinational Association of Supportive Care in Cancer | 10.1007/s00520-007-0342-8 | Other type of intervention |
| Abrahamson, V., Wilson, P., Barclay, S., Brigden, C., Gage, H., Greene, K., Hashem, F., Mikelyte, R., Rees-Roberts, M., Silsbury, G., Goodwin, M., Swash, B., Wee, B., Williams, P., & Butler, C. | Family carer experiences of hospice care at home: Qualitative findings from a mixed methods realist evaluation. | 2023 | Palliat Med | 10.1177/02692163231206027 | Other type of intervention |
| Actrn, | Acceptance and valued-living in palliative care patients, caregivers and significant others | 2014 | https://trialsearch.who.int/Trial2.aspx?TrialID=ACTRN12614000150640 |  | No empirical research study |
| Ahrens, Joann | The positive impact of hospice care on the surviving spouse | 2005 | Home healthcare nurse |  | No empirical research study |
| Akiyama, A., Numata, K., Mikami, H. | Importance of end-of-life support to minimize caregiver's regret during bereavement of the elderly for better subsequent adaptation to bereavement | 2010 | Arch Gerontol Geriatr | 10.1016/j.archger.2009.03.006 | Other type of intervention |
| Allen, Rebecca S.; Harris, Grant M.; Burgio, Louis D.; Azuero, Casey B.; Miller, Leslie A.; Shin, Hae Jung; Eichorst, Morgan K.; Csikai, Ellen L.; DeCoster, Jamie; Dunn, Linda L.; Kvale, Elizabeth; Parmelee, Patricia | Can senior volunteers deliver reminiscence and creative activity interventions? Results of the legacy intervention family enactment randomized controlled trial | 2014 | Journal of Pain & Symptom Management | 10.1016/j.jpainsymman.2013.11.012 | No bereavement outcomes assessed |
| Allen, Rebecca S.; Hilgeman, Michelle M.; Ege, Margaret A.; Shuster, John L., Jr.; Burgio, Louis D. | Legacy activities as interventions approaching the end of life | 2008 | Journal of palliative medicine | 10.1089/jpm.2007.0294 | No bereavement outcomes assessed |
| Ammari, A. B. H.; Hendriksen, C.; Rydahl-Hansen, S. | Results from the family and coping oriented palliative homecare intervention study (FamCope)-A randomized controlled trial | 2018 | J Psychosoc Oncol | 10.1080/07347332.2018.1460003 | Other type of intervention |
| Ando, Michiyo; Morita, Tatsuya; Miyashita, Mitsunori; Sanjo, Makiko; Kira, Haruko; Shima, Yasuo | Effects of bereavement life review on spiritual well-being and depression | 2010 | Journal of pain and symptom management | 10.1016/j.jpainsymman.2009.12.028 | Other type of intervention |
| Ando, Michiyo; Morita, Tatsuya; Miyashita, Mitsunori; Sanjo, Makiko; Kira, Haruko; Shima, Yasuo | Factors that influence the efficacy of bereavement life review therapy for spiritual well-being: a qualitative analysis | 2010 | Supportive care in cancer : official journal of the Multinational Association of Supportive Care in Cancer | 10.1007/s00520-010-1006-7 | Other type of intervention |
| Aoun, S. M.; Abel, J.; Rumbold, B.; Cross, K.; Moore, J.; Skeers, P.; Deliens, L. | The Compassionate Communities Connectors model for end-of-life care: a community and health service partnership in Western Australia | 2020 | Palliat Care Soc Pract | 10.1177/2632352420935130 | No empirical research study |
| Aoun, S. M.; Grande, G.; Howting, D.; Deas, K.; Toye, C.; Troeung, L.; Stajduhar, K.; Ewing, G. | The impact of the carer support needs assessment tool (CSNAT) in community palliative care using a stepped wedge cluster trial | 2015 | PloS one | 10.1371/journal.pone.0123012 | No bereavement outcomes assessed |
| Aoun, S.; Deas, K.; Toye, C.; Ewing, G.; Grande, G.; Stajduhar, K. | Supporting family caregivers to identify their own needs in end-of-life care: Qualitative findings from a stepped wedge cluster trial | 2015 | Palliat Med | 10.1177/0269216314566061 | No bereavement outcomes assessed |
| Aoun, Samar M.; Breen, Lauren J.; Rumbold, Bruce; Howting, Denise | Reported experiences of bereavement support in Western Australia: a pilot study | 2014 | Australian and New Zealand journal of public health | 10.1111/1753-6405.12177 | Other type of intervention |
| Aoun, Samar M.; Connors, Sianne Lee; Priddis, Lynn; Breen, Lauren J.; Colyer, Sue | Motor Neurone Disease family carers' experiences of caring, palliative care and bereavement: an exploratory qualitative study | 2012 | Palliative medicine | 10.1177/0269216311416036 | Other type of intervention |
| Aparicio, M.; Centeno, C.; Carrasco, J. M.; Barbosa, A.; Arantzamendi, M. | What are families most grateful for after receiving palliative care? Content analysis of written documents received: a chance to improve the quality of care | 2017 | BMC Palliat Care | 10.1186/s12904-017-0229-5 | Other type of intervention |
| Ates, G.; Ebenau, A. F.; Busa, C.; Csikos, A.; Hasselaar, J.; Jaspers, B.; Menten, J.; Payne, S.; Van Beek, K.; Varey, S.; Groot, M.; Radbruch, L. | "Never at ease" - family carers within integrated palliative care: a multinational, mixed method study | 2018 | BMC Palliat Care | 10.1186/s12904-018-0291-7 | No bereavement outcomes assessed |
| Bachner, Y. G.; Guldin, M. B.; Nielsen, M. K. | Mortality communication and post-bereavement depression among Danish family caregivers of terminal cancer patients | 2021 | Support Care Cancer | 10.1007/s00520-020-05685-6 | Other type of intervention |
| Bainbridge, D.; Bryant, D.; Seow, H. | Capturing the Palliative Home Care Experience From Bereaved Caregivers Through Qualitative Survey Data: Toward Informing Quality Improvement | 2017 | J Pain Symptom Manage | 10.1016/j.jpainsymman.2016.08.007 | Other type of intervention |
| Bainbridge, D.; Giruparajah, M.; Zou, H.; Seow, H. | The care experiences of patients who die in residential hospice: A qualitative analysis of the last three months of life from the views of bereaved caregivers | 2018 | Palliat Support Care | 10.1017/S147895151700058X | Other type of intervention |
| Baker, Deborah A. | The hospice role in bereavement and loss recovery | 2008 |  |  | Not peer-reviewed |
| Bakitas, M.; Dionne-Odom, J. N.; Pamboukian, S. V.; Tallaj, J.; Kvale, E.; Swetz, K. M.; Frost, J.; Wells, R.; Azuero, A.; Keebler, K.; Akyar, I.; Ejem, D.; Steinhauser, K.; Smith, T.; Durant, R.; Kono, A. T. | Engaging patients and families to create a feasible clinical trial integrating palliative and heart failure care: results of the ENABLE CHF-PC pilot clinical trial | 2017 | BMC Palliat Care | 10.1186/s12904-017-0226-8 | Other type of intervention |
| Barlund, A. S.; Andre, B.; Sand, K.; Brenne, A. T. | A qualitative study of bereaved family caregivers: feeling of security, facilitators and barriers for rural home care and death for persons with advanced cancer | 2021 | BMC Palliat Care | 10.1186/s12904-020-00705-y | Other type of intervention |
| Bauman, J. R.; Schleicher, S. M.; Nipp, R.; El-Jawahri, A.; Pirl, W. F.; Greer, J. A.; Temel, J. S. | Enhancing communication between oncology care providers and patient caregivers during hospice | 2018 | Journal of Community and Supportive Oncology | 10.12788/jcso.0391 | Other setting |
| Bellamy, Gary; Gott, Merryn; Waterworth, Susan; McLean, Christine; Kerse, Ngaire | 'But I do believe you've got to accept that that's what life's about': older adults living in New Zealand talk about their experiences of loss and bereavement support | 2014 | Health & social care in the community | 10.1111/hsc.12069 | Other setting |
| Benkel, I.; Wijk, H.; Molander, U. | Family and friends provide most social support for the bereaved | 2009 | Palliative medicine | 10.1177/0269216308098798 | Other type of intervention |
| Benkel, Inger; Wijk, Helle; Molander, Ulla | Managing grief and relationship roles influence which forms of social support the bereaved needs | 2009 | The American journal of hospice & palliative care | 10.1177/1049909108330034 | Other type of intervention |
| Benson, J. J.; Oliver, D. P.; Washington, K. T.; Rolbiecki, A. J.; Lombardo, C. B.; Garza, J. E.; Demiris, G. | Online social support groups for informal caregivers of hospice patients with cancer | 2020 | Eur J Oncol Nurs | 10.1016/j.ejon.2019.101698 | Other type of intervention |
| Bergman, Elizabeth J.; Haley, William E. | Depressive symptoms, social network, and bereavement service utilization and preferences among spouses of former hospice patients | 2009 | Journal of palliative medicine | 10.1089/jpm.2008.0237 | No bereavement outcomes assessed |
| Bigi, S.; Borelli, E.; Potenza, L.; Gilioli, F.; Artioli, F.; Porzio, G.; Luppi, M.; Bandieri, E. | Early palliative care for solid and blood cancer patients and caregivers: Quantitative and qualitative results of a long-term experience as a case of value-based medicine | 2023 | Front Public Health | 10.3389/fpubh.2023.1092145 | Other type of intervention |
| Bindley, K.; May, A.; Pavlek, W.; Coller, S.; Kalkandis, P.; Dalgleish, T. | Navigating the Liminal Space: Trauma, Transition, and Connection in Bereaved Carers' Experiences of Specialist Palliative Care in Western Sydney | 2018 | J Soc Work End Life Palliat Care | 10.1080/15524256.2018.1495140 | Other type of intervention |
| Blackburn, P.; Bulsara, C. | "You either need help...you feel you don't need help...or you don't feel worthy of asking for it:" Receptivity to bereavement support | 2019 | Palliat Support Care | 10.1017/S1478951517001122 | Other type of intervention |
| Bleidorn, J.; Pahlow, H.; Klindtworth, K.; Schneider, N. | [End-of-life care: experiences and expectations of bereaved relatives] | 2012 | Deutsche medizinische Wochenschrift (1946) | 10.1055/s-0032-1305045 | Other type of intervention |
| Block, Eve M.; Casarett, David J.; Spence, Carol; Gozalo, Pedro; Connor, Stephen R.; Teno, Joan M. | Got volunteers? Association of hospice use of volunteers with bereaved family members' overall rating of the quality of end-of-life care | 2010 | Journal of pain and symptom management | 10.1016/j.jpainsymman.2009.11.310 | Other type of intervention |
| Bolotin, S. M. | Bereavement program develops initiative with universities for win-win partnership: obtains record-breaking community outcomes | 2008 | Caring |  | No empirical research study |
| Breen, L. J.; Aoun, S. M.; Rumbold, B.; McNamara, B.; Howting, D. A.; Mancini, V. | Building Community Capacity in Bereavement Support | 2017 | Am J Hosp Palliat Care | 10.1177/1049909115615568 | Other type of intervention |
| Cagle, John G.; Kovacs, Pamela J. | Informal caregivers of cancer patients: perceptions about preparedness and support during hospice care | 2011 | Journal of gerontological social work | 10.1080/01634372.2010.534547 | No bereavement outcomes assessed |
| Cain, R.; MacLean, M.; Sellick, S. | Giving support and getting help: informal caregivers' experiences with palliative care services | 2004 | Palliat Support Care | 10.1017/s1478951504040350 | Other type of intervention |
| Calik, K. Y.; Kucuk, E.; Halimoglu, B. | The effect of an educational palliative care intervention on the quality of life of patients with incurable cancer and their caregivers | 2022 | Support Care Cancer | 10.1007/s00520-021-06672-1 | Other type of intervention |
| Carlebach, Sarit; Shucksmith, Janet | A review of an out-of-hours telephone support service for palliative care patients and their families | 2010 | International journal of palliative nursing | 10.12968/ijpn.2010.16.9.78647 | Other type of intervention |
| Caserta, M. S.; Lund, D. A.; Utz, R. L.; Tabler, J. L. | "One Size Doesn't Fit All" - Partners in Hospice Care, an Individualized Approach to Bereavement Intervention | 2016 | Omega (Westport) | 10.1177/0030222815575895 | No empirical research study |
| Chan, H. Y. L.; Lee, D. T. F.; Woo, J. | Diagnosing Gaps in the Development of Palliative and End-of-Life Care: A Qualitative Exploratory Study | 2019 | Int J Environ Res Public Health | 10.3390/ijerph17010151 | Other type of intervention |
| Chan, L. M.; Ng, S. C. J. | Prevalence and Correlates of Caregiver Anxiety in Family Caregivers of Patients With Advanced Cancer: a Cross-Sectional Study in a Palliative Care Unit in Hong Kong | 2022 | East Asian Arch Psychiatry | 10.12809/eaap2171 | Other type of intervention |
| Chen, W.; Ma, H.; Wang, X.; Chen, J. | Effects of a Death Education Intervention for Older People with Chronic Disease and Family Caregivers: A Quasi-Experimental Study | 2020 | Asian Nurs Res (Korean Soc Nurs Sci) | 10.1016/j.anr.2020.08.002 | Other setting |
| Cherlin, Emily J.; Barry, Colleen L.; Prigerson, Holly G.; Green, Dena Schulman; Johnson-Hurzeler, Rosemary; Kasl, Stanislav V.; Bradley, Elizabeth H. | Bereavement services for family caregivers: how often used, why, and why not | 2007 | Journal of palliative medicine | 10.1089/jpm.2006.0108 | No bereavement outcomes assessed |
| Ciemins, E. L.; Brant, J.; Kersten, D.; Mullette, E.; Dickerson, D. | A qualitative analysis of patient and family perspectives of palliative care | 2015 | J Palliat Med | 10.1089/jpm.2014.0155 | Other type of intervention |
| Clark, Paul G.; Brethwaite, Drucilla S.; Gnesdiloff, Sabine | Providing support at time of death from cancer: results of a 5-year post-bereavement group study | 2011 | Journal of social work in end-of-life & palliative care | 10.1080/15524256.2011.593156 | Other type of intervention |
| Cloyes, K. G., Reynaga, M., Vega, M., Thomas Hebdon, M. C., Thompson, C., Rosenkranz, S. J., Tay, D., Reblin, M., & Ellington, L. | The Burden of having to Wonder: Hospice Caregiving Experiences of LGBTQ+ Cancer Family Caregivers. Am J Hosp Palliat Care, 41(1), 56-62. | 2024 |  | 10.1177/10499091231159089 | Other type of intervention |
| Cohen-Mansfield, J.; Skornick-Bouchbinder, M.; Cohen, R.; Brill, S. | Treatment and Communication-That Is What Matters: An Analysis of Complaints Regarding End-of-Life Care | 2017 | J Palliat Med | 10.1089/jpm.2017.0002 | Other setting |
| Csikai, E. L. | Bereaved hospice caregivers' perceptions of the end-of-life care communication process and the involvement of health care professionals | 2006 | Journal of palliative medicine | 10.1089/jpm.2006.9.1300 | Other type of intervention |
| Cullen, I., Bailes, M., Shropshire, P., Perry, S., & Karlekar, M. | Connecting Families to Bereavement Resources: A Hospital-Based, Bereavement Follow-Up Pilot During First-Wave COVID-19 [Article in Press]. J Palliat Med. | 2024 |  | 10.1089/jpm.2023.0375 | No bereavement outcomes assessed |
| Demiris, G.; Oliver, D. P.; Washington, K.; Pike, K. | A Problem-Solving Intervention for Hospice Family Caregivers: A Randomized Clinical Trial | 2019 | J Am Geriatr Soc | 10.1111/jgs.15894 | Other type of intervention |
| Dewhurst, F., Tomkow, L., Poole, M., McLellan, E., Kunonga, T. P., Damisa, E., Stowell, M., Todd, C., & Hanratty, B. | Unrepresented, unheard and discriminated against: A qualitative exploration of relatives' and professionals' views of palliative care experiences of people of African and Caribbean descent during the COVID-19 pandemic. Palliat Med, 37(9), 1447-1460. | 2024 |  | 10.1177/02692163231188156 | Other type of intervention |
| Dionne-Odom, J. N.; Azuero, A.; Lyons, K.; Hull, J.; Bakitas, M. | Family Caregiver Grief and Depression Outcomes from the ENABLE III Randomized Controlled Trial | 2016 | Journal of pain and symptom management | 10.1016/j.jpainsymman.2015.12.218 | Not peer-reviewed |
| Dionne-Odom, J. N.; Azuero, A.; Lyons, K.; Hull, J.; Tosteson, T.; Li, Z. Z.; Bakitas, M. | Family caregiver grief and depression outcomes from the ENABLE III randomized controlled trial | 2015 | Journal of Clinical Oncology | 10.1200/jco.2015.33.29_suppl.48 | Not peer-reviewed |
| Dionne-Odom, J. N.; Azuero, A.; Taylor, R. A.; Dosse, C.; Bechthold, A. C.; Currie, E.; Reed, R. D.; Harrell, E. R.; Engler, S.; Ejem, D. B.; Ivankova, N. V.; Martin, M. Y.; Rocque, G. B.; Williams, G. R.; Bakitas, M. A. | A lay navigator-led, early palliative care intervention for African American and rural family caregivers of individuals with advanced cancer (Project Cornerstone): Results of a pilot randomized trial | 2022 | Cancer | 10.1002/cncr.34044 | Other type of intervention |
| Dionne-Odom, J. N.; Ejem, D. B.; Wells, R.; Azuero, A.; Stockdill, M. L.; Keebler, K.; Sockwell, E.; Tims, S.; Engler, S.; Kvale, E.; Durant, R. W.; Tucker, R. O.; Burgio, K. L.; Tallaj, J.; Pamboukian, S. V.; Swetz, K. M.; Bakitas, M. A. | Effects of a Telehealth Early Palliative Care Intervention for Family Caregivers of Persons With Advanced Heart Failure: The ENABLE CHF-PC Randomized Clinical Trial | 2020 | JAMA Netw Open | 10.1001/jamanetworkopen.2020.2583 | Other type of intervention |
| Dobrof, Judith; Ebenstein, Helene; Dodd, Sarah-Jane; Epstein, Irwin; Christ, Grace; Blacker, Susan | Caregivers and Professionals Partnership Caregiver Resource Center: Assessing a Hospital Support Program for Family Caregivers | 2006 | Journal of palliative medicine | 10.1089/jpm.2006.9.196 | Other type of intervention |
| Dosser, Isabel; Kennedy, Catriona | Family carers' experiences of support at the end of life: carers' and health professionals' views | 2012 | International journal of palliative nursing | 10.12968/ijpn.2012.18.10.491 | Other setting |
| Downar, J.; Vanderspank-Wright, B. | Supporting bereaved family members: three steps in the right direction | 2022 | Lancet | 10.1016/S0140-6736(21)02446-6 | Other setting |
| Duke, S.; Campling, N.; May, C. R.; Lund, S.; Lunt, N.; Hospital to Home Co-researcher, group; Richardson, A. | Co-construction of the family-focused support conversation: a participatory learning and action research study to implement support for family members whose relatives are being discharged for end-of-life care at home or in a nursing home | 2020 | BMC Palliat Care | 10.1186/s12904-020-00647-5 | Other type of intervention |
| Dunham, M. | Importance of family counselling for anticipatory grief in cancer care | 2023 | Evid Based Nurs | 10.1136/ebnurs-2022-103577 | No empirical research study |
| Eastman, Peter; Le, Brian; Pharaoh, Amy | The establishment and initial outcomes of a palliative care bereavement service | 2012 | Palliative medicine | 10.1177/0269216312438931 | No empirical research study |
| Egerod, I.; Kaldan, G.; Shaker, S. B.; Guldin, M. B.; Browatski, A.; Marsaa, K.; Overgaard, D. | Spousal bereavement after fibrotic interstitial lung disease: A qualitative study | 2019 | Respir Med | 10.1016/j.rmed.2018.12.008 | Other type of intervention |
| Ekeström, Marie-Louise; Olsson, Mariann; Runesdotter, Sara; Fürst, Carl Johan | Family members' experiences of the impact of the LCP in a palliative care unit and a geriatric ward in Sweden | 2014 | International journal of palliative nursing | 10.12968/ijpn.2014.20.8.381 | No bereavement outcomes assessed |
| El-Jawahri, A.; Greer, J. A.; Pirl, W. F.; Park, E. R.; Jackson, V. A.; Back, A. L.; Kamdar, M.; Jacobsen, J.; Chittenden, E. H.; Rinaldi, S. P.; Gallagher, E. R.; Eusebio, J. R.; Fishman, S.; VanDusen, H.; Li, Z.; Muzikansky, A.; Temel, J. S. | Effects of Early Integrated Palliative Care on Caregivers of Patients with Lung and Gastrointestinal Cancer: A Randomized Clinical Trial | 2017 | Oncologist | 10.1634/theoncologist.2017-0227 | Other type of intervention |
| Emanuel, E. J.; Fairclough, D. L.; Wolfe, P.; Emanuel, L. L. | Talking with terminally ill patients and their caregivers about death, dying, and bereavement: is it stressful? Is it helpful? | 2004 | Arch Intern Med | 10.1001/archinte.164.18.1999 | No bereavement outcomes assessed |
| Empeño, Jessica; Raming, Natasha T. J.; Irwin, Scott A.; Nelesen, Richard A.; Lloyd, Linda S. | The impact of additional support services on caregivers of hospice patients and hospice social workers | 2013 | Omega | 10.2190/OM.67.1-2.f | Other type of intervention |
| Eriksson, Elina; Arve, Seija; Lauri, Sirkka | Informational and emotional support received by relatives before and after the cancer patient's death | 2006 | European journal of oncology nursing : the official journal of European Oncology Nursing Society | 10.1016/j.ejon.2005.04.003 | No bereavement outcomes assessed |
| Ewing, Gail; Brundle, Caroline; Payne, Sheila; Grande, Gunn | The Carer Support Needs Assessment Tool (CSNAT) for use in palliative and end-of-life care at home: a validation study | 2013 | Journal of pain and symptom management | 10.1016/j.jpainsymman.2012.09.008 | Other type of intervention |
| Exley, Catherine; Tyrer, Freya | Bereaved carers' views of a hospice at home service | 2005 | International journal of palliative nursing | 10.12968/ijpn.2005.11.5.242 | No bereavement outcomes assessed |
| Feder, S.; Smith, D.; Griffin, H.; Shreve, S. T.; Kinder, D.; Kutney-Lee, A.; Ersek, M. | "Why Couldn't I Go in To See Him?" Bereaved Families' Perceptions of End-of-Life Communication During COVID-19 | 2021 | J Am Geriatr Soc | 10.1111/jgs.16993 | Other setting |
| Field, David; Payne, Sheila; Relf, Marilyn; Reid, David | Some issues in the provision of adult bereavement support by UK hospices | 2007 | Social science & medicine (1982) | 10.1016/j.socscimed.2006.08.040 | No bereavement outcomes assessed |
| Fisker, Tove; Strandmark, Margaretha | Experiences of surviving spouse of terminally ill spouse: a phenomenological study of an altruistic perspective | 2007 | Scandinavian journal of caring sciences | 10.1111/j.1471-6712.2007.00466.x | Other type of intervention |
| Flanagan-Kaminsky, Donnamarie | Intentional anticipatory mourning, caregiver and bereavement support program for terminally ill veterans, their families & caregivers in the VA Contract Home Hospice Program | 2013 | Omega | 10.2190/OM.67.1-2.h | Other type of intervention |
| Frame, Abbey; Grant, Janie Busby; Layard, Elizabeth; Scholz, Brett; Law, Eleanor; Ranse, Kristen; Mitchell, Imogen; Chapman, Michael | Bereaved caregivers’ satisfaction with end-of-life care | 2021 | Progress in palliative care | 10.1080/09699260.2021.2005756 | Other setting |
| Fromme, Erik K.; Smith, Alexander K.; Hughes, Mark T.; Brokaw, Frances C.; Rosenfeld, Kenneth E.; Arnold, Robert M. | Associations between end-of-life discussions, patient mental health, medical care near death, and caregiver bereavement adjustment | 2010 | American Journal of Hospice & Palliative Medicine | 10.1177/1049909110370745 | Other type of intervention |
| Funk, Laura M.; Allan, Diane E.; Stajduhar, Kelli I. | Palliative family caregivers' accounts of health care experiences: the importance of "security" | 2009 | Palliative & supportive care | 10.1017/S1478951509990447 | Other type of intervention |
| Funk, Laura M.; Stajduhar, Kelli I.; Robin Cohen, S.; Heyland, Daren K.; Williams, Allison | Legitimising and rationalising in talk about satisfaction with formal healthcare among bereaved family members | 2012 | Sociology of health & illness | 10.1111/j.1467-9566.2011.01457.x | Other type of intervention |
| Gomez-Batiste, X.; Mateo-Ortega, D.; Lasmarias, C.; Novellas, A.; Espinosa, J.; Beas, E.; Ela, S.; Barbero, J.; La Caixa, Foundation | Enhancing psychosocial and spiritual palliative care: Four-year results of the program of comprehensive care for people with advanced illnesses and their families in Spain | 2017 | Palliat Support Care | 10.1017/S1478951516000857 | Other population |
| Gómez-Batiste, Xavier; Buisan, Montse; González, M. Pau; Velasco, David; de Pascual, Verónica; Espinosa, Jose; Novellas, Anna; Martínez-Muñoz, Marisa; Simón, Marc; Calle, Candela; Lanaspa, Jaume; Breitbart, William | The "La Caixa" Foundation and WHO Collaborating Center Spanish National Program for enhancing psychosocial and spiritual palliative care for patients with advanced diseases, and their families: preliminary findings | 2011 | Palliative & supportive care | 10.1017/S1478951511000198 | Other population |
| Grande, G. E.; Farquhar, M. C.; Barclay, S. I.; Todd, C. J. | Caregiver bereavement outcome: relationship with hospice at home, satisfaction with care, and home death | 2004 | J Palliat Care |  | Other type of intervention |
| Grande, G. E.; Farquhar, M. C.; Barclay, S. I.; Todd, C. J. | Valued aspects of primary palliative care: content analysis of bereaved carers' descriptions | 2004 | Br J Gen Pract |  | Other setting |
| Greene, Aine; Aranda, Sanchia; Tieman, Jennifer J.; Fazekas, Belinda; Currow, David C. | Can assessing caregiver needs and activating community networks improve caregiver-defined outcomes? A single-blind, quasi-experimental pilot study: community facilitator pilot | 2012 | Palliative medicine | 10.1177/0269216311421834 | Other type of intervention |
| Hannon, B.; Swami, N.; Rodin, G.; Pope, A.; Zimmermann, C. | Experiences of patients and caregivers with early palliative care: A qualitative study | 2017 | Palliat Med | 10.1177/0269216316649126 | Other type of intervention |
| Harris, H.; Lee, C.; Yancey, G. | Cognition in Adult Bereavement: Preliminary Findings From Five Hospice Bereavement Focus Groups | 2015 | J Soc Work End Life Palliat Care | 10.1080/15524256.2015.1115801 | Other type of intervention |
| Hebert, Randy S.; Copeland, Valire C.; Schulz, Richard; Amato, Charlene A.; Arnold, Robert M. | Preparing family caregivers for the death of a loved one: Implications for hospital social workers | 2008 | Journal of social work in end-of-life & palliative care | 10.1080/15524250903081533 | No bereavement outcomes assessed |
| Hebert, Randy S.; Schulz, Richard; Copeland, Valire C.; Arnold, Robert M. | Preparing family caregivers for death and bereavement. Insights from caregivers of terminally ill patients | 2009 | Journal of pain and symptom management | 10.1016/j.jpainsymman.2007.12.010 | No bereavement outcomes assessed |
| Hegarty, M. M.; Abernethy, A. P.; Olver, I.; Currow, D. C. | Former palliative caregivers who identify that additional spiritual support would have been helpful in a population survey | 2011 | Palliative medicine | 10.1177/0269216310389225 | No bereavement outcomes assessed |
| Heyland, Daren K.; Cook, Deborah J.; Rocker, Graeme M.; Dodek, Peter M.; Kutsogiannis, Demetrios J.; Skrobik, Yoanna; Jiang, Xuran; Day, Andrew G.; Cohen, S. Robin | Defining priorities for improving end-of-life care in Canada | 2010 | CMAJ : Canadian Medical Association journal = journal de l'Association medicale canadienne | 10.1503/cmaj.100131 | Other type of intervention |
| Hilliker, Laurel Elizabeth | Who am i now? experiences with formal grief care among Michigan Baby Boomers after spousal loss | 2012 |  |  | Not peer-reviewed |
| Hiratsuka, R.; Aoyama, M.; Masukawa, K.; Shimizu, Y.; Hamano, J.; Sakaguchi, Y.; Watanabe, M.; Morita, T.; Kizawa, Y.; Tsuneto, S.; Shima, Y.; Miyashita, M. | The Association of Family Functioning With Possible Major Depressive Disorders and Complicated Grief Among Bereaved Family Members of Patients With Cancer: Results From the J-HOPE4 Study, a Nationwide Cross-Sectional Follow-Up Survey in Japan | 2021 | J Pain Symptom Manage | 10.1016/j.jpainsymman.2021.06.006 | Other type of intervention |
| Holdsworth, L. M.; Gage, H.; Coulton, S.; King, A.; Butler, C. | A quasi-experimental controlled evaluation of the impact of a hospice rapid response community service for end-of-life care on achievement of preferred place of death | 2015 | Palliat Med | 10.1177/0269216315582124 | Other type of intervention |
| Holland, D. E.; Vanderboom, C. E.; Dose, A. M.; Moore, D.; Robinson, K. V.; Wild, E.; Stiles, C.; Ingram, C.; Mandrekar, J.; Borah, B.; Taylor, E.; Griffin, J. M. | Death and Grieving for Family Caregivers of Loved Ones With Life-Limiting Illnesses in the Era of COVID-19: Considerations for Case Managers | 2021 | Prof Case Manag | 10.1097/NCM.0000000000000485 | No empirical research study |
| Holland, J. M.; Currier, J. M.; Kirkendall, A.; Keene, J. R.; Luna, N. | Sadness, anxiety, and experiences with emotional support among veteran and nonveteran patients and their families at the end of life | 2014 | J Palliat Med | 10.1089/jpm.2013.0485 | Other type of intervention |
| Holland, J. M.; Keene, J. R.; Kirkendall, A.; Luna, N. | Family evaluation of hospice care: Examining direct and indirect associations with overall satisfaction and caregiver confidence | 2015 | Palliat Support Care | 10.1017/S1478951514000595 | Other type of intervention |
| Holm, M.; Arestedt, K.; Carlander, I.; Furst, C. J.; Wengstrom, Y.; Ohlen, J.; Alvariza, A. | Short-term and long-term effects of a psycho-educational group intervention for family caregivers in palliative home care - results from a randomized control trial | 2016 | Psychooncology | 10.1002/pon.4004 | Other type of intervention |
| Holmberg, Lena | 'Words that made a difference': Communication in bereavement | 2007 | Journal of Loss and Trauma | 10.1080/15325020600725778 | Other type of intervention |
| Holtslander, L.; Duggleby, W.; Teucher, U.; Cooper, D.; Bally, J. M.; Solar, J.; Steeves, M. | Developing and pilot-testing a Finding Balance Intervention for older adult bereaved family caregivers: A randomized feasibility trial | 2016 | Eur J Oncol Nurs | 10.1016/j.ejon.2016.01.003 | Other setting |
| Holyoke, P.; Stephenson, B. | Organization-level principles and practices to support spiritual care at the end of life: a qualitative study | 2017 | BMC Palliat Care | 10.1186/s12904-017-0197-9 | Other type of intervention |
| Hotchkiss, J., Ridderman, E., & Buftin, W. | Overall US Hospice Quality According to Decedent Caregivers-Natural Language Processing and Sentiment Analysis of 3389 Online Caregiver Reviews [Article in Press]. Am J Hosp Palliat Care, 10499091231185593. | 2023 |  | 10.1177/10499091231185593 | Other type of intervention |
| Hottensen, Dory | Bereavement: caring for families and friends after a patient dies | 2013 | Omega | 10.2190/OM.67.1-2.n | No empirical research study |
| Houben, C. H. M.; Spruit, M. A.; Luyten, H.; Pennings, H. J.; van den Boogaart, V. E. M.; Creemers, Jphm; Wesseling, G.; Wouters, E. F. M.; Janssen, D. J. A. | Cluster-randomised trial of a nurse-led advance care planning session in patients with COPD and their loved ones | 2019 | Thorax | 10.1136/thoraxjnl-2018-211943 | Other population |
| Hovland, C. | When Death With Dementia Is "A Memory Seared in My Brain": Caregivers' Recommendations to Health Care Professionals | 2020 | J Appl Gerontol | 10.1177/0733464819884267 | No bereavement outcomes assessed |
| Hudson, J.; Reblin, M.; Clayton, M. F.; Ellington, L. | Addressing cancer patient and caregiver role transitions during home hospice nursing care | 2019 | Palliat Support Care | 10.1017/S1478951518000214 | Other type of intervention |
| Hudson, P.; Trauer, T.; Kelly, B.; O'Connor, M.; Thomas, K.; Summers, M.; Zordan, R.; White, V. | Reducing the psychological distress of family caregivers of home-based palliative care patients: Short-term effects from a randomised controlled trial | 2013 | Psycho-oncology | 10.1002/pon.3242 | Other type of intervention |
| Hudson, Peter L.; Lobb, Elizabeth A.; Thomas, Kristina; Zordan, Rachel D.; Trauer, Tom; Quinn, Karen; Williams, Anne; Summers, Michael | Psycho-Educational Group Intervention for Family Caregivers of Hospitalized Palliative Care Patients: Pilot Study | 2012 | Journal of palliative medicine | 10.1089/jpm.2011.0347 | No bereavement outcomes assessed |
| Jack, B. A.; Mitchell, T. K.; Cope, L. C.; O'Brien, M. R. | Supporting older people with cancer and life-limiting conditions dying at home: a qualitative study of patient and family caregiver experiences of Hospice at Home care | 2016 | J Adv Nurs | 10.1111/jan.12983 | Other type of intervention |
| Jack, B. A.; O'Brien, M. R.; Scrutton, J.; Baldry, C. R.; Groves, K. E. | Supporting family carers providing end-of-life home care: a qualitative study on the impact of a hospice at home service | 2015 | J Clin Nurs | 10.1111/jocn.12695 | Other type of intervention |
| Johnston, B.; Bowman, F.; Carduff, E.; Donmez, F.; Lowndes, A.; McKeown, A. | 'Playlist for Life' at the end of life: a mixed-methods feasibility study of a personalised music listening intervention in the hospice setting | 2022 | Pilot Feasibility Stud | 10.1186/s40814-022-00983-8 | Other type of intervention |
| Johnston, B.; Coole, C.; Jay Narayanasamy, M. | An end-of-life care nurse service for people with COPD and heart failure: stakeholders' experiences | 2016 | Int J Palliat Nurs | 10.12968/ijpn.2016.22.11.549 | Other type of intervention |
| Jung, Yeojung; Yeom, Hyun- E.; Lee, Na-Ri | The Effects of Counseling about Death and Dying on Perceptions, Preparedness, and Anxiety Regarding Death among Family Caregivers Caring for Hospice Patients: A Pilot Study | 2021 | The Korean Journal of Hospice and Palliative Care | 10.14475/jhpc.2021.24.1.46 | No bereavement outcomes assessed |
| Kang, Jina; Shin, Dong Wook; Choi, Ji Eun; Sanjo, Makiko; Yoon, Soo Jin; Kim, Hwan Kyun; Oh, Myoung Suk; Kwen, Hyang Suk; Choi, Hae Young; Yoon, Wook Hee | Factors associated with positive consequences of serving as a family caregiver for a terminal cancer patient | 2013 | Psycho-oncology | 10.1002/pon.3033 | Other type of intervention |
| Khan Joad, Anjum S.; Mayamol, T. C.; Chaturvedi, Mohita | What Does the Informal Caregiver of a Terminally III Cancer Patient Need? A Study from a Cancer Centre | 2011 | Indian journal of palliative care | 10.4103/0973-1075.92335 | Other type of intervention |
| Kim, M. | End-of-life concierge service aims to help families cope with details | 2016 | Mod Healthc |  | No empirical research study |
| Kissane, D. W.; Zaider, T. I.; Li, Y.; Hichenberg, S.; Schuler, T.; Lederberg, M.; Lavelle, L.; Loeb, R.; Del Gaudio, F. | Randomized Controlled Trial of Family Therapy in Advanced Cancer Continued Into Bereavement | 2016 | J Clin Oncol | 10.1200/JCO.2015.63.0582 | Other type of intervention |
| Kissane, David; Lichtenthal, Wendy G.; Zaider, Talia | Family care before and after bereavement | 2007 | Omega | 10.2190/om.56.1.c | Other type of intervention |
| Kokou-Kpolou, C. K.; Moukouta, C. S.; Sani, L.; McIntee, S. E.; Cenat, J. M.; Awesso, A.; Bacque, M. F. | A Mixed Methods Approach of End-of-Life Care, Social Rites, and Bereavement Outcomes: A Transnational Perspective | 2020 | Cult Med Psychiatry | 10.1007/s11013-020-09669-3 | Other type of intervention |
| Kutner, J. S. | Applying the evidence base to terminal care | 2005 | Journal of palliative medicine | 10.1089/jpm.2005.8.1040 | No empirical research study |
| Kutney-Lee, A., Rodriguez, K. L., Ersek, M., & Carthon, J. M. B. | "They Did Not Know How to Talk to Us and It Seems That They Didn't Care:" Narratives from Bereaved Family Members of Black Veterans [Article in Press]. J Racial Ethn Health Disparities. | 2023 |  | 10.1007/s40615-023-01790-4 | Other type of intervention |
| Kutney-Lee, A.; Smith, D.; Thorpe, J.; Del Rosario, C.; Ibrahim, S.; Ersek, M. | Race/Ethnicity and End-of-Life Care Among Veterans | 2017 | Med Care | 10.1097/MLR.0000000000000637 | Other type of intervention |
| Kwak, Jung; Kramer, Betty J.; Lang, Josh; Ledger, Maria | Challenges in End-of-Life Care Management for Low-Income Frail Elders: A Case Study of the Wisconsin Family Care Program | 2013 | Research on Aging | 10.1177/0164027512446939 | Other type of intervention |
| Lavalley, S. A. | Caregiver informational support in different patient care settings at end of life | 2018 | Home Health Care Serv Q | 10.1080/01621424.2018.1438951 | Other type of intervention |
| Law, M. C.; Lau, B. H.; Kwok, A. Y. Y.; Lee, J. S. H.; Lui, R. N. Y.; Liu, K. H.; Leung, P. P. Y.; Chan, C. L. W. | Empowering families facing end-stage nonmalignant chronic diseases with a holistic, transdisciplinary, community-based intervention: 3 months outcome of the Life Rainbow Program | 2021 | Palliat Support Care | 10.1017/S1478951520001224 | No bereavement outcomes assessed |
| MacKinnon, C. J.; Smith, N. G.; Henry, M.; Milman, E.; Chochinov, H. M.; Korner, A.; Berish, M.; Farrace, A. J.; Liarikos, N.; Cohen, S. R. | Reconstructing Meaning with Others in Loss: A Feasibility Pilot Randomized Controlled Trial of a Bereavement Group | 2015 | Death Stud | 10.1080/07481187.2014.958628 | Other setting |
| MacLeod, Ann; Skinner, Mark W.; Low, Eleanor | Supporting hospice volunteers and caregivers through community-based participatory research | 2012 | Health & social care in the community | 10.1111/j.1365-2524.2011.01030.x | Other type of intervention |
| Maeda, I.; Miyashita, M.; Yamagishi, A.; Kinoshita, H.; Shirahige, Y.; Izumi, N.; Yamaguchi, T.; Igarashi, M.; Kato, M.; Morita, T. | Changes in Relatives' Perspectives on Quality of Death, Quality of Care, Pain Relief, and Caregiving Burden Before and After a Region-Based Palliative Care Intervention | 2016 | J Pain Symptom Manage | 10.1016/j.jpainsymman.2016.03.022 | Other type of intervention |
| Magill, L. | Caregiver empowerment and music therapy: Through the eyes of bereaved caregivers of advanced cancer patients | 2009 | Journal of palliative care |  | Other type of intervention |
| Maloney, Cristine; Lyons, Kathleen Doyle; Li, Zhongze; Hegel, Mark; Ahles, Tim A.; Bakitas, Marie | Patient perspectives on participation in the ENABLE II randomized controlled trial of a concurrent oncology palliative care intervention: Benefits and burdens | 2013 | Palliative medicine | 10.1177/0269216312445188 | Other type of intervention |
| Marti-Garcia, C.; Fernandez-Alcantara, M.; Suarez Lopez, P.; Romero Ruiz, C.; Munoz Martin, R.; Garcia-Caro, M. P. | Experiences of family caregivers of patients with terminal disease and the quality of end-of-life care received: a mixed methods study | 2020 | PeerJ | 10.7717/peerj.10516 | Other type of intervention |
| Matthys, O.; Dierickx, S.; Deliens, L.; Lapeire, L.; Hudson, P.; Van Audenhove, C.; De Vleminck, A.; Cohen, J. | How are family caregivers of people with a serious illness supported by healthcare professionals in their caregiving tasks? A cross-sectional survey of bereaved family caregivers | 2022 | Palliat Med | 10.1177/02692163211070228 | Other type of intervention |
| McCarthy, Sylvia Ann; Jenn, Ng Chirk; Leng, Caroline Chin Kwong; Hamzah, Ednin | What are the experiences and needs of patients and carers receiving community palliative care in Malaysia? A qualitative study | 2016 | Progress in palliative care | 10.1179/1743291x14y.0000000099 | Other type of intervention |
| McKay, Elizabeth A.; Taylor, Ann E.; Armstrong, Claire | "What she told us made the world of difference": carers' perspectives on a hospice at home service | 2013 | Journal of palliative care |  | Other type of intervention |
| McKinlay, E.; Vaipuna, K.; O'Toole, T.; Golds, H.; Adams, A. | Doing what it takes: a qualitative study of New Zealand carers' experiences of giving home-based palliative care to loved ones | 2021 | N Z Med J |  | Other type of intervention |
| McLaughlin, Dorry; Sullivan, Kate; Hasson, Felicity | Hospice at home service: the carer's perspective | 2007 | Supportive care in cancer : official journal of the Multinational Association of Supportive Care in Cancer | 10.1007/s00520-006-0110-1 | Other type of intervention |
| McMillan, Susan C.; Small, Brent J.; Weitzner, Michael; Schonwetter, Ronald; Tittle, Mary; Moody, Linda; Haley, William E. | Impact of coping skills intervention with family caregivers of hospice patients with cancer: a randomized clinical trial | 2006 | Cancer | 10.1002/cncr.21567 | Other type of intervention |
| McNamara, Beverley; Rosenwax, Lorna | Which carers of family members at the end of life need more support from health services and why? | 2010 | Social science & medicine (1982) | 10.1016/j.socscimed.2009.11.029 | No bereavement outcomes assessed |
| Milberg, A.; Liljeroos, M.; Krevers, B. | Can a single question about family members' sense of security during palliative care predict their well-being during bereavement? A longitudinal study during ongoing care and one year after the patient's death | 2019 | BMC Palliat Care | 10.1186/s12904-019-0446-1 | Other type of intervention |
| Milberg, Anna; Rydstrand, Kristina; Helander, Lena; Friedrichsen, Maria | Participants' experiences of a support group intervention for family members during ongoing palliative home care | 2005 | Journal of palliative care |  | Other type of intervention |
| Miyashita, M.; Aoyama, M.; Yoshida, S.; Yamada, Y.; Abe, M.; Yanagihara, K.; Shirado, A.; Shutoh, M.; Okamoto, Y.; Hamano, J.; Miyamoto, A.; Nakahata, M. | The distress and benefit to bereaved family members of participating in a post-bereavement survey | 2018 | Jpn J Clin Oncol | 10.1093/jjco/hyx177 | Other type of intervention |
| Miyashita, M.; Morita, T.; Hirai, K. | Evaluation of end-of-life cancer care from the perspective of bereaved family members: The Japanese experience | 2008 | Journal of Clinical Oncology | 10.1200/JCO.2007.15.8287 | Other type of intervention |
| Miyashita, M.; Morita, T.; Sato, K.; Tsuneto, S.; Shima, Y. | A Nationwide Survey of Quality of End-of-Life Cancer Care in Designated Cancer Centers, Inpatient Palliative Care Units, and Home Hospices in Japan: The J-HOPE Study | 2015 | J Pain Symptom Manage | 10.1016/j.jpainsymman.2015.01.007 | Other type of intervention |
| Mogan, C.; Harrison Dening, K.; Dowrick, C.; Lloyd-Williams, M. | Health and social care services for people with dementia at home at the end of life: A qualitative study of bereaved informal caregivers' experiences | 2022 | Palliat Med | 10.1177/02692163221092624 | Other type of intervention |
| Moon, H., Journ, S., & Lee, S. | Effect of Laughter Therapy on Mood Disturbances, Pain, and Burnout in Terminally Ill Cancer Patients and Family Caregivers. Cancer Nurs, 47(1), 3-11. | 2024 |  | 10.1097/NCC.0000000000001162 | Other type of intervention |
| Moore, K. J.; Goodison, H.; Sampson, E. L. | The role of the memory service in helping carers to prepare for end of life: A mixed methods study | 2019 | Int J Geriatr Psychiatry | 10.1002/gps.5034 | Other population |
| Morita, Tatsuya; Miyashita, Mitsunori; Yamagishi, Akemi; Akiyama, Miki; Akizuki, Nobuya; Hirai, Kei; Imura, Chizuru; Kato, Masashi; Kizawa, Yoshiyuki; Shirahige, Yutaka; Yamaguchi, Takuhiro; Eguchi, Kenji | Effects of a programme of interventions on regional comprehensive palliative care for patients with cancer: a mixed-methods study | 2013 | The Lancet. Oncology | 10.1016/S1470-2045(13)70127-X | No bereavement outcomes assessed |
| Morris, S. E.; Nayak, M. M.; Block, S. D. | Insights from Bereaved Family Members about End-of-Life Care and Bereavement | 2020 | J Palliat Med | 10.1089/jpm.2019.0467 | Other setting |
| Mossin, Hanne; Landmark, Bjørg Th | Being present in hospital when the patient is dying—A grounded theory study of spouses experiences | 2011 | European Journal of Oncology Nursing | 10.1016/j.ejon.2010.11.005 | Other type of intervention |
| Muders, P.; Zahrt-Omar, C. A.; Bussmann, S.; Haberstroh, J.; Weber, M. | Support for families of patients dying with dementia: a qualitative analysis of bereaved family members' experiences and suggestions | 2015 | Palliat Support Care | 10.1017/S1478951513001107 | No bereavement outcomes assessed |
| Murray, K.; Breiddal, S. | Presenting three education resources: palliative care manual, online program, and bereavement journal group | 2006 | Journal of palliative care |  | No empirical research study |
| Neergaard, Mette Asbjoern; Olesen, Frede; Jensen, Anders Bonde; Sondergaard, Jens | Palliative care for cancer patients in a primary health care setting: Bereaved relatives' experience, a qualitative group interview study | 2008 | BMC palliative care | 10.1186/1472-684X-7-1 | Other type of intervention |
| Ng, A. Y. M.; Wong, F. K. Y. | Effects of a Home-Based Palliative Heart Failure Program on Quality of Life, Symptom Burden, Satisfaction and Caregiver Burden: A Randomized Controlled Trial | 2018 | J Pain Symptom Manage | 10.1016/j.jpainsymman.2017.07.047 | Other type of intervention |
| Nyatanga, B. | Supporting the bereaved in palliative care | 2019 | Br J Community Nurs | 10.12968/bjcn.2019.24.8.399 | No empirical research study |
| O'Connor, M.; Smith, G.; Pantaleo, A.; Haywood, D.; Weaver, R.; Halkett, G. K. | "It's Always Been a Second Class Cancer": An Exploration of the Experiences and Journeys of Bereaved Family Carers of People with Sarcoma | 2021 | Cancers (Basel) | 10.3390/cancers13112670 | Other type of intervention |
| Omoyeni, N.; Soyannwo, O.; Aikomo, O.; Iken, O. | Home-based palliative care for adult cancer patients in Ibadan-a three year review | 2014 | ecancermedicalscience | 10.3332/ecancer.2014.490 | No bereavement outcomes assessed |
| Oosterveld-Vlug, M. G.; Custers, B.; Hofstede, J.; Donker, G. A.; Rijken, P. M.; Korevaar, J. C.; Francke, A. L. | What are essential elements of high-quality palliative care at home? An interview study among patients and relatives faced with advanced cancer | 2019 | BMC Palliat Care | 10.1186/s12904-019-0485-7 | Other type of intervention |
| O'Sullivan, A.; Alvariza, A.; Ohlen, J.; Larsdotter, C. | Support received by family members before, at and after an ill person's death | 2021 | BMC Palliat Care | 10.1186/s12904-021-00800-8 | Other setting |
| Otani, H.; Yoshida, S.; Morita, T.; Aoyama, M.; Kizawa, Y.; Shima, Y.; Tsuneto, S.; Miyashita, M. | Meaningful Communication Before Death, but Not Present at the Time of Death Itself, Is Associated With Better Outcomes on Measures of Depression and Complicated Grief Among Bereaved Family Members of Cancer Patients | 2017 | J Pain Symptom Manage | 10.1016/j.jpainsymman.2017.07.010 | Other type of intervention |
| Parsons, Sheila; Anderson, Colleen | The meaning of Friday afternoon tea for informal caregivers on a palliative care unit | 2009 | International journal of palliative nursing | 10.12968/ijpn.2009.15.2.39804 | No bereavement outcomes assessed |
| Paul, A.; Fernandes, E. | Experiences of Caregivers in a Home-Based Palliative Care Model - A Qualitative Study | 2020 | Indian J Palliat Care | 10.4103/IJPC.IJPC_154_19 | Other type of intervention |
| Pavaskar, Chandragupta | Volunteers in Palliative Care: An Experience in South Australia | 2018 |  | 10.4103/IJPC.IJPC_192_17 | No empirical research study |
| Perner, Andrea; Köhler, Norbert; Brähler, Elmar; Götze, Heide | [Quality of life and satisfaction of family caregivers in palliative care - results of postmortem interviews with bereaved family members] | 2012 | Zeitschrift fur Psychosomatische Medizin und Psychotherapie | 10.13109/zptm.2012.58.3.267 | Other type of intervention |
| Pickersgill, F.; Dean, E. | Groundbreaking bereavement service proves to be a winner | 2011 | Nursing Standard | 10.7748/ns.25.35.10.s14 | No empirical research study |
| Pooler, C.; Richman-Eisenstat, J.; Kalluri, M. | Early integrated palliative approach for idiopathic pulmonary fibrosis: A narrative study of bereaved caregivers' experiences | 2018 | Palliat Med | 10.1177/0269216318789025 | Other type of intervention |
| Poppe, C.; Iseli, L. M.; Verwey, M.; Wangmo, T. | Bereavement and Support Experiences of Informal Caregivers of Persons with Amyotrophic Lateral Sclerosis: A Qualitative Study | 2022 | J Soc Work End Life Palliat Care | 10.1080/15524256.2021.1976352 | Other setting |
| Potvin, Noah | A music therapy model for pre-bereavement resiliency development in informal hospice caregivers: A grounded theory study | 2018 |  |  | Not peer-reviewed |
| Reed, R. V. | Don't forget bereaved siblings | 2011 | BMJ | 10.1136/bmj.d2705 | No empirical research study |
| Rhodes, Ramona L.; Mitchell, Susan L.; Miller, Susan C.; Connor, Stephen R.; Teno, Joan M. | Bereaved family members' evaluation of hospice care: what factors influence overall satisfaction with services? | 2008 | Journal of pain and symptom management | 10.1016/j.jpainsymman.2007.12.004 | Other type of intervention |
| Rickerson, Elizabeth; Harrold, Joan; Kapo, Jennifer; Carroll, Janet T.; Casarett, David | Timing of hospice referral and families' perceptions of services: are earlier hospice referrals better? | 2005 | Journal of the American Geriatrics Society | 10.1111/j.1532-5415.2005.53259.x | Other type of intervention |
| Riggs, J. S.; Woodby, L. L.; Burgio, K. L.; Bailey, F. A.; Williams, B. R. | "Don't get weak in your compassion": bereaved next of kin's suggestions for improving end-of-life care in Veterans Affairs Medical Centers | 2014 | J Am Geriatr Soc | 10.1111/jgs.12764 | Other type of intervention |
| Robinson, J.; Goodwin, H.; Williams, L.; Anderson, N.; Parr, J.; Irwin, R.; Gott, M. | A task service and a talking service: A qualitative exploration of bereaved family perceptions of community nursing care at the end of life | 2022 | Palliat Med | 10.1177/02692163221127168 | Other type of intervention |
| Rogalla, K. B. | Anticipatory Grief, Proactive Coping, Social Support, and Growth: Exploring Positive Experiences of Preparing for Loss | 2020 | Omega (Westport) | 10.1177/0030222818761461 | Other type of intervention |
| Roper, L.; Donnellan, W.; Hanratty, B.; Bennett, K. | Exploring dimensions of social support and resilience when providing care at the end of life: a qualitative study | 2019 | Aging Ment Health | 10.1080/13607863.2018.1484886 | Other type of intervention |
| Sampson, Elizabeth L.; Jones, Louise; Thuné-Boyle, Ingela C. V.; Kukkastenvehmas, Riitta; King, Michael; Leurent, Baptiste; Tookman, Adrian; Blanchard, Martin R. | Palliative assessment and advance care planning in severe dementia: an exploratory randomized controlled trial of a complex intervention | 2011 | Palliative medicine | 10.1177/0269216310391691 | Other type of intervention |
| Sekelja, Natasha; Butow, Phyllis N.; Tattersall, Martin H. N. | Bereaved cancer carers' experience of and preference for palliative care | 2010 | Supportive care in cancer : official journal of the Multinational Association of Supportive Care in Cancer | 10.1007/s00520-009-0752-x | Other type of intervention |
| Small, Neil; Barnes, Sarah; Gott, Merryn; Payne, Sheila; Parker, Chris; Seamark, David; Gariballa, Salah | Dying, death and bereavement: a qualitative study of the views of carers of people with heart failure in the UK | 2009 | BMC palliative care | 10.1186/1472-684X-8-6 | Other setting |
| Smith, Paula | The family caregivers journey in end-of-life care: Recognizing and identifying with the role of carer | 2009 | International Journal on Disability and Human Development | 10.1515/IJDHD.2009.8.1.67 | No empirical research study |
| Spelten, E.; Timmis, J.; Heald, S.; Duijts, S. F. A. | Rural palliative care to support dying at home can be realised; experiences of family members and nurses with a new model of care | 2019 | Aust J Rural Health | 10.1111/ajr.12518 | No bereavement outcomes assessed |
| Stahl, S. T.; Emanuel, J.; Albert, S. M.; Dew, M. A.; Schulz, R.; Robbins-Welty, G.; Reynolds, C. F., 3rd | Design and Rationale for a Technology-based Healthy Lifestyle Intervention in Older Adults Grieving the Loss of a Spouse | 2017 | Contemp Clin Trials Commun | 10.1016/j.conctc.2017.09.002 | Other setting |
| Steinhauser, Karen E.; Voils, Corrine I.; Bosworth, Hayden; Tulsky, James A. | What constitutes quality of family experience at the end of life? Perspectives from family members of patients who died in the hospital | 2015 | Palliative & supportive care | 10.1017/S1478951514000807 | Other setting |
| Stringer, Kathy B. | A qualitative study into the current process of death education for caregivers of terminal patients | 2010 |  |  | Not peer-reviewed |
| Sun, V.; Kim, J. Y.; Irish, T. L.; Borneman, T.; Sidhu, R. K.; Klein, L.; Ferrell, B. | Palliative care and spiritual well-being in lung cancer patients and family caregivers | 2016 | Psychooncology | 10.1002/pon.3987 | No bereavement outcomes assessed |
| Thomas, K.; Hudson, P.; Trauer, T.; Remedios, C.; Clarke, D. | Risk factors for developing prolonged grief during bereavement in family carers of cancer patients in palliative care: a longitudinal study | 2014 | J Pain Symptom Manage | 10.1016/j.jpainsymman.2013.05.022 | Other type of intervention |
| Uchida, T.; Satake, N.; Nakaho, T.; Inoue, A.; Saito, H. | Bereavement risk assessment of family caregivers of patients with cancer: Japanese version of the Bereavement Risk Assessment Tool | 2019 | Palliat Support Care | 10.1017/S1478951518000755 | Other type of intervention |
| Ullrich, A.; Marx, G.; Bergelt, C.; Benze, G.; Zhang, Y.; Wowretzko, F.; Heine, J.; Dickel, L. M.; Nauck, F.; Bokemeyer, C.; Oechsle, K. | Supportive care needs and service use during palliative care in family caregivers of patients with advanced cancer: a prospective longitudinal study | 2021 | Support Care Cancer | 10.1007/s00520-020-05565-z | No bereavement outcomes assessed |
| Vale-Taylor, P. | "We will remember them": a mixed-method study to explore which post-funeral remembrance activities are most significant and important to bereaved people living with loss, and why those particular activities are chosen | 2009 | Palliative medicine | 10.1177/0269216309103803 | No bereavement outcomes assessed |
| van Oosterhout, S. P. C.; Ermers, D. J. M.; Ploos van Amstel, F. K.; van Herpen, C. M. L.; Schoon, Y.; Perry, M.; van Geel, M.; Kuip, E. J. M.; Engels, Y. | Experiences of bereaved family caregivers with shared decision making in palliative cancer treatment: a qualitative interview study | 2021 | BMC Palliat Care | 10.1186/s12904-021-00833-z | Other type of intervention |
| Verkissen, M. N.; Leemans, K.; Van den Block, L.; Deliens, L.; Cohen, J. | Information provision as evaluated by people with cancer and bereaved relatives: A cross-sectional survey of 34 specialist palliative care teams | 2019 | Patient Educ Couns | 10.1016/j.pec.2018.11.012 | No bereavement outcomes assessed |
| Vermorgen, M.; De Vleminck, A.; Leemans, K.; Van den Block, L.; Van Audenhove, C.; Deliens, L.; Cohen, J. | Family carer support in home and hospital: a cross-sectional survey of specialised palliative care | 2020 | BMJ Support Palliat Care | 10.1136/bmjspcare-2019-001795 | No bereavement outcomes assessed |
| Wakenshaw, Clare; Sillence, Elizabeth | From a good death to a better bereavement? The impact of the end of life experience on bereavement adjustment, a thematic analysis | 2018 | Bereavement Care | 10.1080/02682621.2018.1535878 | Other type of intervention |
| Waldrop, Deborah P. | Caregiving systems at the end of life: How informal caregivers and formal providers collaborate | 2006 | Families in Society | 10.1606/1044-3894.3548 | Other type of intervention |
| Walsh, Trish; Foreman, Maeve; Curry, Philip; O'Driscoll, Siobhan; McCormack, Martin | Bereavement support in an acute hospital: an Irish model | 2008 | Death studies | 10.1080/07481180802289788 | Other setting |
| Walshe, C., Mateus, C., Varey, S., Dodd, S., Cockshott, Z., Filipe, L., & Brearley, S. G. | Thank goodness you're here'. Exploring the impact on patients, family carers and staff of enhanced 7-day specialist palliative care services: A mixed methods study. Palliat Med, 37(10), 1484-1497. | 2023 |  | 10.1177/02692163231201486 | Other type of intervention |
| Ware, O. D.; Cagle, J. G. | Informal Caregiving Networks for Hospice Patients With Cancer and Their Impact on Outcomes: A Brief Report | 2019 | Am J Hosp Palliat Care | 10.1177/1049909118792011 | Other type of intervention |
| Warrier, M. G.; Thomas, P. T.; Sadasivan, A.; Balasubramaniam, B.; Vengalil, S.; Nashi, S.; Preethish-Kumar, V.; Polavarapu, K.; Mahajan, N. P.; Chevula, P. C. R.; Nalini, A. | Family Caregivers' Experiences with Dying and Bereavement of Individuals with Motor Neuron Disease in India | 2019 | J Soc Work End Life Palliat Care | 10.1080/15524256.2019.1645081 | Other type of intervention |
| Wei, E.; Segall, J.; Villanueva, Y.; Dang, L. B.; Gasca, V. I.; Gonzalez, M. P.; Roman, M.; Mendez-Justiniano, I.; Cohen, A. G.; Cho, H. J. | Coping With Trauma, Celebrating Life: Reinventing Patient And Staff Support During The COVID-19 Pandemic | 2020 | Health Aff (Millwood) | 10.1377/hlthaff.2020.00929 | No empirical research study |
| Weibull, A.; Olesen, F.; Neergaard, M. A. | Caregivers' active role in palliative home care - To encourage or to dissuade? A qualitative descriptive study | 2008 | BMC palliative care | 10.1186/1472-684X-7-15 | Other type of intervention |
| Wen, F. H.; Chou, W. C.; Hou, M. M.; Su, P. J.; Shen, W. C.; Chen, J. S.; Chang, W. C.; Hsu, M. H.; Tang, S. T. | Associations of death-preparedness states with bereavement outcomes for family caregivers of terminally ill cancer patients | 2022 | Psychooncology | 10.1002/pon.5827 | Other type of intervention |
| Wiese, Christoph H. R.; Morgenthal, Hannah C.; Bartels, Utz E.; Vossen-Wellmann, Andrea; Graf, Bernhard M.; Hanekop, Gerd G. | Post-mortal bereavement of family caregivers in Germany: a prospective interview-based investigation | 2010 | Wiener klinische Wochenschrift | 10.1007/s00508-010-1396-z | Other type of intervention |
| Winslow, Michelle; Smith, Sam | OA19 Can oral history in palliative care influence the well-being of participants and the bereaved? | 2015 | BMJ supportive & palliative care | 10.1136/bmjspcare-2015-000906.19 | No empirical research study |
| Wintermeyer-Pingel, Susan A.; Murphy, Donna; Hammelef, Karen J. | Improving a grief and loss program: caring for patients, families, and staff | 2013 | Omega | 10.2190/OM.67.1-2.z3 | Other population |
| Xiu, D.; Chow, A. Y. M.; Tang, S. | Predictive factors for differential changes in grief symptoms following group bereavement intervention for Chinese widowed older adults | 2020 | Clin Psychol Psychother | 10.1002/cpp.2425 | Other setting |
| Zimmermann, C.; Swami, N.; Krzyzanowska, M.; Leighl, N.; Rydall, A.; Rodin, G.; Tannock, I.; Hannon, B. | Perceptions of palliative care among patients with advanced cancer and their caregivers | 2016 | CMAJ | 10.1503/cmaj.151171 | Other type of intervention |
| Zomerdijk, N.; Panozzo, S.; Mileshkin, L.; Yoong, J.; Nowak, A. K.; Stockler, M. R.; Philip, J. | Palliative care facilitates the preparedness of caregivers for thoracic cancer patients | 2022 | Eur J Cancer Care (Engl) | 10.1111/ecc.13716 | Other type of intervention |

# **Supplementary file 5: Quality assessment**

## **5.1 Quantitative studies**

RCTs

| **Study ID** | **Title** | **Screening: Are there clear research questions?** | **Screening: Are there clear research questions? supporting text** | **Screening: Do the collected data allow to address the research question(s)?** | **Screening: Do the collected data allow to address the research question(s)? supporting text** | **RCT: Is randomization appropriately performed?** | **RCT: Is randomization appropriately performed? supporting text** | **RCT: Are the groups comparable at baseline?** | **RCT: Are the groups comparable at baseline? supporting text** | **RCT: Are there complete outcome data?** | **RCT: Are there complete outcome data? supporting text** | **RCT: Are outcome assessors blinded to the intervention provided?** | **RCT: Are outcome assessors blinded to the intervention provided? supporting text** | **RCT: Did the participants adhere to the assigned intervention?** | **RCT: Did the participants adhere to the assigned intervention? supporting text** | **Overall Quality** |
| --- | --- | --- | --- | --- | --- | --- | --- | --- | --- | --- | --- | --- | --- | --- | --- | --- |
| Dionne-Odom 2015 | Family Caregiver Depressive Symptom and Grief Outcomes From the ENABLE III Randomized Controlled Trial | Yes | Aim stated | Yes | Aim stated | Can't tell | 2016: ...were randomized into early and delayed intervention groups...(p5) | No | Employment status (p=0.05) | No |  | Yes |  | Can't tell |  | 20% |
| Hudson 2015 | Reducing the psychological distress of family caregivers of home based palliative care patients: longer term effects from a randomised controlled trial | Yes | Purpose stated | Yes |  | Can't tell | "Participants were randomised to either the control or intervention groups" | Can't tell | No comprehensive data on that topic provided. Almost statistically significant differences in sex between intervention arms. Mean age of men was sign. higher than woman. | No | 160/298 completed t3 data collection | Yes |  | Yes |  | 40% |
| Grande 2017 | Assessing the impact of a Carer Support Needs Assessment Tool (CSNAT) intervention in palliative home care: a stepped wedge cluster trial | Yes | Aim of study reported | Yes |  | No | Pragmatic constraints meant the sequence of sites beginning the intervention was not fully randomised; the sites most and least ready to fit in a practice change at study start were allocated to begin the intervention first (CSNAT intervention only) and last (control only), respectively, in the implementation sequence. The remaining sites were allocated to begin treatment in a randomised sequence in between these two. | Yes |  | Yes | Missing data in survey response sample 0-11% | Yes | Postbereavement measurement furthermore meant outcome data collection proceeded a step behind, and was managed separately from, practice delivery of the intervention, so assessment was fully blinded and intervention and data collection activities did not interfere with each other. | No | However, the process data indicate that implementation of the CSNAT intervention was limited, both in terms of numbers of carers affected and the extent to which the intervention was delivered as intended. | 60% |

RCTs continued

| **Study ID** | **Title** | **Screening: Are there clear research questions?** | **Screening: Are there clear research questions? supporting text** | **Screening: Do the collected data allow to address the research question(s)?** | **Screening: Do the collected data allow to address the research question(s)? supporting text** | **RCT: Is randomization appropriately performed?** | **RCT: Is randomization appropriately performed? supporting text** | **RCT: Are the groups comparable at baseline?** | **RCT: Are the groups comparable at baseline? supporting text** | **RCT: Are there complete outcome data?** | **RCT: Are there complete outcome data? supporting text** | **RCT: Are outcome assessors blinded to the intervention provided?** | **RCT: Are outcome assessors blinded to the intervention provided? supporting text** | **RCT: Did the participants adhere to the assigned intervention?** | **RCT: Did the participants adhere to the assigned intervention? supporting text** | **Overall Quality** |
| --- | --- | --- | --- | --- | --- | --- | --- | --- | --- | --- | --- | --- | --- | --- | --- | --- |
| Hudson 2005 | A psycho-educational intervention for family caregivers of patients receiving palliative care: a randomized controlled trial | No |  | Yes |  | Yes | via computer-generated software system | Can't tell |  | No | Intervention: baseline n=54, T2 n=40, t3 n=20; comparison: baseline n=52, t2 n=35, t3 n= 25) | Can't tell | Not reported | Can't tell |  | 20% |
| vonHeymann-Horan 2018 | Effect of home-based specialised palliative care and dyadic psychological intervention on caregiver anxiety and depression: a randomised controlled trial | Yes | aim stated | Yes |  | Yes |  | Yes |  | No |  | Can't tell |  | Yes |  | 60% |
| Davis 2020 | Feasibility randomised controlled trial of a self-help acceptance and commitment therapy intervention for grief and psychological distress in carers of palliative care patients | Yes | Aim stated | Yes |  | Yes | Participants were randomised according to  computer-generated random numbers performed by a research assistant blinded to the identity of participants. | Yes |  | No | N randomised baseline/1-month/6-months: Intervention: 53/35/26/20, control: 53/20/18/9 | No |  | Can't tell |  | 40% |
| Walsh 2007 | Reducing emotional distress in people caring for patients receiving specialist palliative care. Randomised trial | Yes | aim stated | Yes |  | Yes |  | Yes |  | No |  | Can't tell |  | Yes |  | 60% |
| Mooney 2024 | Impact of an automated, remote monitoring and coaching intervention in reducing hospice cancer family caregiving burden: A multisite randomized controlled trial | Yes | aim stated | Yes |  | Yes |  | Can't tell |  | Yes |  | No |  | Yes |  | 60% |

Non RCTs

| **Study ID** | **Title** | **Screening: Are there clear research questions?** | **Screening: Are there clear research questions? supporting text** | **Screening: Do the collected data allow to address the research question(s)?** | **Screening: Do the collected data allow to address the research question(s)? supporting text** | **Non RCT: Are the participants representative of the target population?** | **Non RCT: Are the participants representative of the target population? supporting text** | **Non RCT: Are measurements appropriate regarding both the outcome and intervention (or exposure)?** | **Non RCT: Are measurements appropriate regarding both the outcome and intervention (or exposure)? supporting text** | **Non RCT: Are there complete outcome data?** | **Non RCT: Are there complete outcome data? supporting text** | **Non RCT: Are the confounders accounted for in the design and analysis?** | **Non RCT: Are the confounders accounted for in the design and analysis? supporting text** | **Non RCT: During the study period, is the intervention administered (or exposure occurred) as intended?** | **Non RCT: During the study period, is the intervention administered (or exposure occurred) as intended? supporting text** | **Overall Quality** |
| --- | --- | --- | --- | --- | --- | --- | --- | --- | --- | --- | --- | --- | --- | --- | --- | --- |
| Veerbeek 2008 | Using the LCP: bereaved relatives' assessments of communication and bereavement | Yes | aim stated | Yes |  | Yes |  | Yes |  | No | 59 % of all eligible questionnaires filled in | Can't tell |  | Can't tell |  | 40% |
| Petursdottir 2020 | The Impact of Receiving a Family-Oriented Therapeutic Conversation Intervention Before and During Bereavement Among Family Cancer Caregivers: A Nonrandomized Trial | Yes | aim stated | Yes |  | Yes |  | Yes |  | Yes | complete retention; no withdrawal in either group | Can't tell |  | Yes |  | 80% |
| Nappa 2016 | The effect of bereavement groups on grief, anxiety, and depression - a controlled, prospective intervention study | Yes | Aim stated | Yes |  | Yes |  | Yes |  | No | 31-49% response rate | No |  | Yes |  | 60% |

Quantitative descriptive designs

| **Study ID** | **Title** | **Screening: Are there clear research questions?** | **Screening: Are there clear research questions? supporting text** | **Screening: Do the collected data allow to address the research question(s)?** | **Screening: Do the collected data allow to address the research question(s)? supporting text** | **Quantitative descriptive: Is the sampling strategy relevant to address the research question?** | **Quantitative descriptive: Is the sampling strategy relevant to address the research question? supporting text** | **Quantitative descriptive: Is the sample representative of the target population?** | **Quantitative descriptive: Is the sample representative of the target population? supporting text** | **Quantitative descriptive: Are the measurements appropriate?** | **Quantitative descriptive: Are the measurements appropriate? supporting text** | **Quantitative descriptive: Is the risk of nonresponse bias low?** | **Quantitative descriptive: Is the risk of nonresponse bias low? supporting text** | **Quantitative descriptive: Is the statistical analysis appropriate to answer the research question?** | **Quantitative descriptive: Is the statistical analysis appropriate to answer the research question? supporting text** | **Overall Quality** |
| --- | --- | --- | --- | --- | --- | --- | --- | --- | --- | --- | --- | --- | --- | --- | --- | --- |
| Goebel 2017 | Family members of deceased palliative care patients receiving bereavement anniversary cards: a survey on the recipient's reactions and opinions | Yes | Aim stated | Yes |  | Yes |  | Can't tell | Response rate from 35% could be the very satisfied persons. The unsatisfied may have not returned the questionnaire. | Yes |  | No | 35% response rate could have introduced bias. | No | They could have calculated medians and could have reported percentages which would have supported the interpretation of results. | 40% |
| Roberts 2008 | The nature and use of bereavement support services in a hospice setting | Yes | Aim of study reported | Yes |  | Yes |  | Can't tell |  | Yes |  | No | Response rate 47% | Yes |  | 60% |
| Reblin 2019 | Communication of emotion in home hospice cancer care: Implications for spouse caregiver depression into bereavement | Yes | Aim stated | Yes |  | Yes |  | Can't tell |  | Yes |  | Can't tell |  | Yes |  | 60% |
| Yamaguchi 2017 | Effects of End-of-Life Discussions on the Mental Health of Bereaved Family Members and Quality of Patient Death and Care | Yes | aim stated | Yes |  | Yes |  | Yes |  | Yes |  | No | Response rate 67 % | Yes |  | 80% |
| Supiano 2020 | Extending our reach: telehealth delivered grief support groups for rural hospice | Yes | aim stated | Yes |  | Yes |  | Can't tell |  | Yes |  | Yes | 92.86% completers | Yes |  | 80% |
| Aoun 2018a | What sources of bereavement support are perceived helpful by bereaved people and why? Empirical evidence for the compassionate communities approach | Yes | Aim stated, specific objetives stated | Yes |  | Yes |  | Yes |  | Yes |  | No | response rate 21.3 % | Yes |  | 80% |
| Aoun 2017 | Bereavement support for family caregivers: The gap between guidelines and practice in palliative care | Yes | Aim stated | Yes |  | Yes |  | Yes |  | Yes |  | No | Response rate 18.1 % (range 13.3-28.6) | Yes |  | 80% |
| Olsson 2017 | Psychosocial Well-Being of Young People Who Participated in a Support Group Following the Loss of a Parent to Cancer | Yes | Aim stated | Yes |  | Yes |  | Yes |  | Can't tell |  | Can't tell |  | Yes |  | 60% |

Quantitative descriptive continued

| **Study ID** | **Title** | **Screening: Are there clear research questions?** | **Screening: Are there clear research questions? supporting text** | **Screening: Do the collected data allow to address the research question(s)?** | **Screening: Do the collected data allow to address the research question(s)? supporting text** | **Quantitative descriptive: Is the sampling strategy relevant to address the research question?** | **Quantitative descriptive: Is the sampling strategy relevant to address the research question? supporting text** | **Quantitative descriptive: Is the sample representative of the target population?** | **Quantitative descriptive: Is the sample representative of the target population? supporting text** | **Quantitative descriptive: Are the measurements appropriate?** | **Quantitative descriptive: Are the measurements appropriate? supporting text** | **Quantitative descriptive: Is the risk of nonresponse bias low?** | **Quantitative descriptive: Is the risk of nonresponse bias low? supporting text** | **Quantitative descriptive: Is the statistical analysis appropriate to answer the research question?** | **Quantitative descriptive: Is the statistical analysis appropriate to answer the research question? supporting text** | **Overall Quality** |
| --- | --- | --- | --- | --- | --- | --- | --- | --- | --- | --- | --- | --- | --- | --- | --- | --- |
| Levesque 2023 | Grief Coach, a Text-Based Grief Support Intervention: Acceptability Among Hospice Family Members | Yes | Formulated as aim. | Yes |  | Yes | users of the programm | Yes | All users of the programm according the clear criteria | Yes |  | Yes | To my knowledge is a response rate of 65% acceptable | Yes |  | 100% |
| Ramstadt 2023 | Experiencing Missing Contact With Professionals and Long Term Bereavement Outcome | Yes | aim stated | Yes |  | Yes |  | Yes |  | Yes |  | No |  | Yes |  | 80% |
| Aoun 2018b | The Impact of Supporting Family Caregivers Before Bereavement on Outcomes After Bereavement: Adequacy of End-of-Life Support and Achievement of Preferred Place of Death | Yes | Aim described | Yes |  | Yes |  | Yes |  | Yes |  | Yes |  | Yes |  | 100% |

## **5.2 Qualitative Studies**

| **Study ID** | **Title** | **Screening: Are there clear research questions?** | **Screening: Are there clear research questions? supporting text** | **Screening: Do the collected data allow to address the research question(s)?** | **Screening: Do the collected data allow to address the research question(s)? supporting text** | **Qualitative: Is the qualitative approach appropriate to answer the research question?** | **Qualitative: Is the qualitative approach appropriate to answer the research question? supporting text** | **Qualitative: Are the qualitative data collection methods adequate to address the research question?** | **Qualitative: Are the qualitative data collection methods adequate to address the research question? supporting text** | **Qualitative: Are the findings adequately derived from the data?** | **Qualitative: Are the findings adequately derived from the data? supporting text** | **Qualitative: Is the interpretation of results sufficiently substantiated by data?** | **Qualitative: Is the interpretation of results sufficiently substantiated by data? supporting text** | **Qualitative: Is there coherence between qualitative data sources, collection, analysis and interpretation?** | **Qualitative: Is there coherence between qualitative data sources, collection, analysis and interpretation? supporting text** | **Overall quality score** |
| --- | --- | --- | --- | --- | --- | --- | --- | --- | --- | --- | --- | --- | --- | --- | --- | --- |
| Agnew 2008 | User perspective on palliative care services: experiences of middle-aged partners bereaved through cancer known to social work services in Northern Ireland | Yes | Aim stated | Yes |  | Yes |  | Yes | Note: collection methods are adequate but they had a fixed sample size of 10 people and didn't continued data collection until saturation. | Yes | 30% double check | Yes |  | Yes |  | 100% |
| Cronfalk 2009 | Inner power, physical strength and existential well-being in daily life: Relatives' experiences of receiving soft tissue massage in palliative home care | Yes | Aim stated | Yes |  | Yes |  | Yes |  | Can't tell |  | Yes |  | Yes |  | 80% |
| Magill 2009b | The meaning of the music: the role of music in palliative care music therapy as perceived by bereaved caregivers of advanced cancer patients | Yes | Aim stated | Yes |  | Yes |  | Yes |  | Can't tell |  | Can't tell |  | Can't tell |  | 40% |
| Magill 2009a | The spiritual meaning of pre-loss music therapy to bereaved caregivers of advanced cancer patients | Yes | Aim stated | Yes |  | Yes |  | Yes |  | Can't tell |  | Can't tell |  | Yes |  | 60% |
| Cronfalk 2010 | Soft tissue massage: early intervention for relatives whose family members died in palliative cancer care | Yes | Aim stated | Yes |  | Yes |  | Yes |  | Yes |  | Can't tell |  | Yes |  | 80% |
| McGrath 2010 | Surviving spousal bereavement--insights for GPs | Yes | Aim stated | Yes |  | Yes |  | Yes |  | Can't tell |  | Can't tell |  | Yes |  | 60% |
| Lundberg 2013 | The perspectives of bereaved family members on their experiences of support in palliative care | Yes | Aim stated | Yes |  | Yes |  | Yes |  | Yes |  | Yes |  | Yes |  | 100% |
| Muta 2014 | What bereavement follow-up does family members request in Japanese palliative care units? A qualitative study | Yes | Aim stated | Yes |  | Yes |  | Yes |  | Yes |  | Yes |  | Yes |  | 100% |

Qualitative studies continued

| **Study ID** | **Title** | **Screening: Are there clear research questions?** | **Screening: Are there clear research questions? supporting text** | **Screening: Do the collected data allow to address the research question(s)?** | **Screening: Do the collected data allow to address the research question(s)? supporting text** | **Qualitative: Is the qualitative approach appropriate to answer the research question?** | **Qualitative: Is the qualitative approach appropriate to answer the research question? supporting text** | **Qualitative: Are the qualitative data collection methods adequate to address the research question?** | **Qualitative: Are the qualitative data collection methods adequate to address the research question? supporting text** | **Qualitative: Are the findings adequately derived from the data?** | **Qualitative: Are the findings adequately derived from the data? supporting text** | **Qualitative: Is the interpretation of results sufficiently substantiated by data?** | **Qualitative: Is the interpretation of results sufficiently substantiated by data? supporting text** | **Qualitative: Is there coherence between qualitative data sources, collection, analysis and interpretation?** | **Qualitative: Is there coherence between qualitative data sources, collection, analysis and interpretation? supporting text** | **Overall quality score** |
| --- | --- | --- | --- | --- | --- | --- | --- | --- | --- | --- | --- | --- | --- | --- | --- | --- |
| Tabler 2015 | Missed Opportunity: Hospice Care and the Family | Yes | Aim stated | Yes |  | Yes |  | Can't tell |  | Can't tell |  | Yes |  | Can't tell |  | 40% |
| Kirby 2018 | The meaning and experience of bereavement support: A qualitative interview study of bereaved family caregivers | Yes | Aim stated | Yes |  | Yes |  | Yes |  | Yes |  | Yes |  | Yes |  | 100% |
| Makarem 2018 | Experiences and Expectations of Bereavement Contact among Caregivers of Patients with Advanced Cancer | Yes | Aim stated | Yes |  | Yes | Exploration of experiences and opinions | Yes |  | Yes |  | Yes |  | Yes |  | 100% |
| Maze 2022 | Perceptions of bereaved family members of nursing care on an inpatient hospice palliative care unit | Yes |  | Yes |  | Yes |  | Yes |  | Yes |  | Yes |  | Yes |  | 100% |
| Nappa 2020 | Experiences of participation in bereavement groups from significant others' perspectives; a qualitative study | Yes | Aim stated | Yes |  | Yes |  | Can't tell | Interview would have been more appropriate | Yes |  | Yes |  | Yes |  | 80% |

## **5.3 Mixed Methods Studies**

Mixed Methods: qualitative part

| **Study ID** | **Title** | **Screening: Are there clear research questions?** | **Screening: Are there clear research questions? supporting text** | **Screening: Do the collected data allow to address the research question(s)?** | **Screening: Do the collected data allow to address the research question(s)? supporting text** | **Qualitative: Is the qualitative approach appropriate to answer the research question?** | **Qualitative: Is the qualitative approach appropriate to answer the research question? supporting text** | **Qualitative: Are the qualitative data collection methods adequate to address the research question?** | **Qualitative: Are the qualitative data collection methods adequate to address the research question? supporting text** | **Qualitative: Are the findings adequately derived from the data?** | **Qualitative: Are the findings adequately derived from the data? supporting text** | **Qualitative: Is the interpretation of results sufficiently substantiated by data?** | **Qualitative: Is the interpretation of results sufficiently substantiated by data? supporting text** | **Qualitative: Is there coherence between qualitative data sources, collection, analysis and interpretation?** | **Qualitative: Is there coherence between qualitative data sources, collection, analysis and interpretation? supporting text** | **Qualitative Score** |
| --- | --- | --- | --- | --- | --- | --- | --- | --- | --- | --- | --- | --- | --- | --- | --- | --- |
| Wittenberg-Lyles 2015 | "It is the 'starting over' part that is so hard": Using an online group to support hospice bereavement | Yes |  | Can't tell |  | Can't tell |  | Can't tell |  | Yes |  | Yes |  | Yes |  | 60% |
| McGinley 2020 | Navigating the Transition from Advanced Illness to Bereavement: How Provider Communication Informs Family-related Roles and Needs | Yes | aim stated | Yes |  | Yes |  | Yes |  | Yes |  | Yes |  | Yes |  | 100% |

Mixed Methods: quantitative part

| **Study ID** | **Title** | **Quantitative descriptive: Is the sampling strategy relevant to address the research question?** | **Quantitative descriptive: Is the sampling strategy relevant to address the research question? supporting text** | **Quantitative descriptive: Is the sample representative of the target population?** | **Quantitative descriptive: Is the sample representative of the target population? supporting text** | **Quantitative descriptive: Are the measurements appropriate?** | **Quantitative descriptive: Are the measurements appropriate? supporting text** | **Quantitative descriptive: Is the risk of nonresponse bias low?** | **Quantitative descriptive: Is the risk of nonresponse bias low? supporting text** | **Quantitative descriptive: Is the statistical analysis appropriate to answer the research question?** | **Quantitative descriptive: Is the statistical analysis appropriate to answer the research question? supporting text** | **Quantitative Score** |
| --- | --- | --- | --- | --- | --- | --- | --- | --- | --- | --- | --- | --- |
| Wittenberg-Lyles 2015 | "It is the 'starting over' part that is so hard": Using an online group to support hospice bereavement | **Yes** |  | **Can't tell** |  | **Yes** |  | **Can't tell** |  | **Yes** |  | **60%** |
| McGinley 2020 | Navigating the Transition from Advanced Illness to Bereavement: How Provider Communication Informs Family-related Roles and Needs | **Yes** |  | **Can't tell** |  | **Yes** |  | **No** | **108 of 325 bereaved included** | **Yes** |  | **60%** |

Mixed Methods: mixed methods part

| **Study ID** | **Title** | **Mixed methods: Is there an adequate rationale for using a mixed methods design to address the research question?** | **Mixed methods: Is there an adequate rationale for using a mixed methods design to address the research question? supporting text** | **Mixed methods: Are the different components of the study effectively integrated to answer the research question?** | **Mixed methods: Are the different components of the study effectively integrated to answer the research question? supporting text** | **Mixed methods: Are the outputs of the integration of qualitative and quantitative components adequately interpreted?** | **Mixed methods: Are the outputs of the integration of qualitative and quantitative components adequately interpreted? supporting text** | **Mixed methods: Are divergences and inconsistencies between quantitative and qualitative results adequately addressed?** | **Mixed methods: Are divergences and inconsistencies between quantitative and qualitative results adequately addressed? supporting text** | **Mixed methods: Do the different components of the study adhere to the quality criteria of each tradition of the methods involved?** | **Mixed methods: Do the different components of the study adhere to the quality criteria of each tradition of the methods involved? supporting text** | **MiMe Score** | **Overall score** |
| --- | --- | --- | --- | --- | --- | --- | --- | --- | --- | --- | --- | --- | --- |
| Wittenberg-Lyles 2015 | "It is the 'starting over' part that is so hard": Using an online group to support hospice bereavement | No |  | No |  | Yes |  | No |  | Can't tell |  | 20% | 20% |
| McGinley 2020 | Navigating the Transition from Advanced Illness to Bereavement: How Provider Communication Informs Family-related Roles and Needs | No |  | Can't tell |  | Can't tell |  | No |  | Can't tell |  | 0% | 0% |

## **5.4 Multi Methods Studies**

Multi Method: qualitative part

| **Study ID** | **Title** | **Screening: Are there clear research questions?** | **Screening: Are there clear research questions? supporting text** | **Screening: Do the collected data allow to address the research question(s)?** | **Screening: Do the collected data allow to address the research question(s)? supporting text** | **Qualitative: Is the qualitative approach appropriate to answer the research question?** | **Qualitative: Is the qualitative approach appropriate to answer the research question? supporting text** | **Qualitative: Are the qualitative data collection methods adequate to address the research question?** | **Qualitative: Are the qualitative data collection methods adequate to address the research question? supporting text** | **Qualitative: Are the findings adequately derived from the data?** | **Qualitative: Are the findings adequately derived from the data? supporting text** | **Qualitative: Is the interpretation of results sufficiently substantiated by data?** | **Qualitative: Is the interpretation of results sufficiently substantiated by data? supporting text** | **Qualitative: Is there coherence between qualitative data sources, collection, analysis and interpretation?** | **Qualitative: Is there coherence between qualitative data sources, collection, analysis and interpretation? supporting text** | **Qualitative Score** |
| --- | --- | --- | --- | --- | --- | --- | --- | --- | --- | --- | --- | --- | --- | --- | --- | --- |
| Hudson 2006 | How well do family caregivers cope after caring for a relative with advanced disease and how can health professionals enhance their support? | Yes | What were the circumstances surrounding the patient’s death?; 2. How well were caregivers coping?; 3. What factors aided or inhibited the bereavement experience?; 4. How might health professionals improve support for family caregivers? | Yes | See section Results 697-700 Seven percent of caregivers were confronted by traumatic grief; most caregivers perceived they were coping reasonably well and could identify positive outcomes related to their experience. Caregivers noted the significant benefits of receiving comprehensive information to prepare them for the future and expressed appreciation for the support provided by specialist palliative care services. | Yes |  | Yes |  | Can't tell |  | Can't tell |  | Yes |  | 60% |
| Wu 2022 | Prolonged Grief and Bereavement Supports Within a Caregiver Population Who Transition Through a Palliative Care Program in British Columbia, Canada | Yes | aim stated | Yes |  | Can't tell |  | No | Open ended survey questions. Interviews would have been more appropriate. | Can't tell |  | Yes |  | Yes |  | 40% |

Multi Method: Quantitative part

| **Study ID** | **Title** | **Quantitative descriptive: Is the sampling strategy relevant to address the research question?** | **Quantitative descriptive: Is the sampling strategy relevant to address the research question? supporting text** | **Quantitative descriptive: Is the sample representative of the target population?** | **Quantitative descriptive: Is the sample representative of the target population? supporting text** | **Quantitative descriptive: Are the measurements appropriate?** | **Quantitative descriptive: Are the measurements appropriate? supporting text** | **Quantitative descriptive: Is the risk of nonresponse bias low?** | **Quantitative descriptive: Is the risk of nonresponse bias low? supporting text** | **Quantitative descriptive: Is the statistical analysis appropriate to answer the research question?** | **Quantitative descriptive: Is the statistical analysis appropriate to answer the research question? supporting text** | **Quantitative Score** | **Non RCT: Are the participants representative of the target population?** | **Non RCT: Are the participants representative of the target population? supporting text** | **Non RCT: Are measurements appropriate regarding both the outcome and intervention (or exposure)?** | **Non RCT: Are measurements appropriate regarding both the outcome and intervention (or exposure)? supporting text** | **Non RCT: Are there complete outcome data?** | **Non RCT: Are there complete outcome data? supporting text** | **Non RCT: Are the confounders accounted for in the design and analysis?** | **Non RCT: Are the confounders accounted for in the design and analysis? supporting text** | **Non RCT: During the study period, is the intervention administered (or exposure occurred) as intended?** | **Non RCT: During the study period, is the intervention administered (or exposure occurred) as intended? supporting text** | **Overall Quality** | **Mean** |
| --- | --- | --- | --- | --- | --- | --- | --- | --- | --- | --- | --- | --- | --- | --- | --- | --- | --- | --- | --- | --- | --- | --- | --- | --- |
| Hudson 2006 | How well do family caregivers cope after caring for a relative with advanced disease and how can health professionals enhance their support? | Yes |  | Can't tell |  | Yes |  | No |  | Yes |  | 60% |  |  |  |  |  |  |  |  |  |  |  | 60% |
| Wu 2022 | Prolonged Grief and Bereavement Supports Within a Caregiver Population Who Transition Through a Palliative Care Program in British Columbia, Canada | Yes |  | Can't tell |  | Yes |  | No | response rate 52.5% | Yes |  | 60% |  |  |  |  |  |  |  |  |  |  |  | 50% |
